# Suicide risk on Christmas Eve, Christmas Day, New Year's Day, and Valentine's Day: a systematic review and meta-analysis

**DOI:** 10.3389/fpubh.2025.1668476

**Published:** 2025-09-30

**Authors:** Ta-Chuan Yeh, Tien-Wei Hsu, Yu-Chen Kao, Trevor Thompson, Brendon Stubbs, Andre F. Carvalho, Fu-Chi Yang, Ping-Tao Tseng, Chih-Wei Hsu, Chia-Ling Yu, Yu-Kang Tu, Chih-Sung Liang

**Affiliations:** ^1^Department of Psychiatry, Tri-Service General Hospital, National Defense Medical Centre, Taipei, Taiwan; ^2^Department of Chemical Engineering and Biotechnology, National Taipei University of Technology, Taipei, Taiwan; ^3^Department of Psychiatry, Zuoying Armed Forces General Hospital, Kaohsiung, Taiwan; ^4^Department of Psychiatry, E-DA Dachang Hospital, I-Shou University, Kaohsiung, Taiwan; ^5^School of Medicine, College of Medicine, I-Shou University, Kaohsiung, Taiwan; ^6^Graduate Institute of Clinical Medicine, College of Medicine, Kaohsiung Medical University, Kaohsiung, Taiwan; ^7^Department of Psychiatry, National Defense Medical Centre, Taipei, Taiwan; ^8^Department of Psychiatry, Beitou Branch, Tri-Service General Hospital, Taipei, Taiwan; ^9^Centre for Chronic Illness and Ageing, University of Greenwich, London, United Kingdom; ^10^Institute of Psychiatry, Psychology and Neuroscience, King's College London, London, United Kingdom; ^11^IMPACT (Innovation in Mental and Physical Health and Clinical Treatment) Strategic Research Centre, School of Medicine, Barwon Health, Deakin University, Geelong, VIC, Australia; ^12^Department of Neurology, Tri-Service General Hospital, National Defense Medical Centre, Taipei, Taiwan; ^13^Institute of Biomedical Sciences, National Sun Yat-sen University, Kaohsiung, Taiwan; ^14^Department of Psychology, College of Medical and Health Science, Asia University, Taichung, Taiwan; ^15^Prospect Clinic for Otorhinolaryngology & Neurology, Kaohsiung, Taiwan; ^16^Institute of Precision Medicine, National Sun Yat-sen University, Kaohsiung, Taiwan; ^17^Department of Psychiatry, Kaohsiung Chang Gung Memorial Hospital and Chang Gung University College of Medicine, Kaohsiung, Taiwan; ^18^Department of Pharmacy, Chang Gung Memorial Hospital Linkou, Taoyuan, Taiwan; ^19^Institute of Health Data Analytics & Statistics, College of Public Health, National Taiwan University, Taipei, Taiwan; ^20^Department of Dentistry, National Taiwan University Hospital, Taipei, Taiwan

**Keywords:** suicide, self-harm and suicide related behavior, Christmas, New Year's Day, Valentine's Day

## Abstract

**Objective:**

Holidays are times of celebration of family and loved ones which can be difficult for some people. This study assessed the risk of suicide on Christmas Eve, Christmas Day, New Year's Day, and Valentine's Day.

**Methods:**

We searched four major electronic databases. The primary outcome was suicide deaths, and the secondary outcome was self-harm and suicide-related behaviors (SHSB). For each holiday, we calculated the risk ratio (RR) compared to regular days and the proportion of annual suicides.

**Results:**

We included 28 studies (*n* = 2,186,094). The proportion of annual suicides was 0.23% [95% confidence interval, 0.17%, 0.28%; number of studies (k) = 11] on Christmas Eve, 0.24% (0.19%, 0.29%; k = 17) on Christmas Day, 0.39% (0.31%, 048%; k = 16) on New Year's Days, and 0.27% (0.24%, 0.30%; k = 5) on Valentine's Day. Compared to regular days, suicide risk was 17% lower (RR = 0.83; 0.72, 0.96) on Christmas Day and 33% higher on New Year's Day (RR = 1.33; 1.06, 1.65) with no significant difference for Christmas Eve or Valentine's Day. This pattern of lower suicide risk on Christmas and higher risk on New Year's Day was consistent across countries. Regarding SHSB, the proportions were 0.19% on Christmas Eve, 0.21% on Christmas Day, 0.29% on New Year's Day, and 0.23% on Valentine's Day, corresponding to a lower risk on Christmas Eve (RR = 0.74; 0.57, 0.96; k = 5) and a higher risk on New Year's Day (RR = 1.17; 1.03, 1.34; k = 6), but no significant difference on Christmas Day or Valentine's Day.

**Conclusion:**

Our study suggests that only New Year's Day appears to be a temporal hotspot for suicide across most countries.

**Systematic review registration:**

Open Science Framework (osf.io/7zx3d).

## 1 Introduction

According to the World Health Organization (WHO), approximately 800,000 people die by suicide (i.e., suicide or suicide deaths) every year, which is about one person every 40 seconds (1). Suicides account for 1.4% of premature deaths worldwide (2). However, the incidence of suicide varies by region. The lower rates are often found in the Eastern Mediterranean and the Europe tend to have higher rates (1). In contrast, the prevalence of suicide deaths is about 5% in suicide attempters (1, 3). Among individuals who engaged in non-fatal self-harm, the rate of suicide deaths within 1 year is 37.2 times higher than that of the matched general population cohort, specifically 439.1 per 100,000 person-years (4). The epidemiology of suicide attempt differs from suicide deaths. The incidence rate of suicide is generally higher among males than females. Men are more likely to die by suicide, while women are more likely to attempt suicide (5). Several risk factors of suicide have been studied and identified. One of the strongest predisposing factors is neuropsychiatric diseases, such as depressive disorder, bipolar disorder, schizophrenia-spectrum disorders, and personality disorders (1, 6). Other predisposing factors are family history of suicidal behavior, adverse childhood experience, previous suicide attempts, and socioeconomic deprivation (1, 7–10). For precipitating factors, evidence suggests drug and alcohol misuse, access to lethal means, and new diagnosis of terminal or chronic physical illness (1, 11, 12).

It is a common myth that the incidence of suicide peaks during major holidays, like Christmas. The psychosocial broken-promise effect supports this myth, referring to the phenomenon where heightened expectations of social connection and support during holiday seasons can lead to significant distress if those expectations are unmet. The resulting frustration and disappointment may trigger suicidal reactions (13, 14). However, Christmas is also a holiday that can evoke feelings of hope and emotional upliftment in individuals, which may exert a protective effect against suicide. Many studies indicate that during Christmas, the rates of suicide deaths, suicide attempt, psychiatric hospitalization, and usage of emergency rooms are lower compared with those on regular days (15–17). On the other hand, New Year's Day is another major holiday and often viewed with hope and optimism, because it represents a fresh start and an opportunity for new beginnings. This day also brings a sense of relief and closure as individuals leave behind the challenges of the past year. However, the societal emphasis on achieving resolutions can also lead to feelings of anxiety and pressure for some individuals. Several studies have showed an increased suicide rates in England (18) and Sweden (19), but not in South Korea (20), or Switzerland (21). Likewise, Valentine's Day is often associated with feelings of love, affection, and romantic gestures, as people celebrate their relationships and express their emotions to loved ones. However, for some, Valentines can be a time of rejection and isolation whether the risk of suicide is higher or lower on Valentine's Day remains inconclusive (22–24).

Beyond static risk factors, emerging research underscores the importance of temporal patterns in suicide risk. Identifying high-risk time windows—whether daily, weekly, or seasonal—enables targeted interventions when individuals are most vulnerable (25). For instance, diurnal studies reveal that suicidal ideation often follows circadian rhythms, with attempts peaking in afternoon/evening hours when isolation and emotional dysregulation intensify (25). Similarly, holidays may disrupt social routines, amplifying or mitigating risk depending on cultural context. Critically, mapping these temporal trends allows healthcare systems to allocate resources (e.g., crisis hotlines, clinician availability) efficiently, challenge stigma by framing suicidality as time-dependent, and ultimately reduce the transition from ideation to action (25). This meta-analysis extends this temporal lens to major holidays, testing whether celebratory periods paradoxically elevate risk through unmet social expectations or protective effects via increased connectivity.

To the best of our knowledge, there are few comprehensive assessments of the risks of suicide deaths and suicide attempt on major holidays. A recent study reported that suicide risk was increased on New Year's Day, but the risk varied on Christmas by countries (26). However, that paper is a non-systematic review, whereas we are using a systematic review approach to address this question (26). We conducted the systematic review and meta-analysis to investigate the proportion and the risk of suicide deaths and suicide attempts on holidays. Our study focused on four major holidays that are widely celebrated across many countries and regions: Christmas, Christmas Eve, New Year's Day, and Valentine's Day. Our study aimed to provide a more nuanced understanding of the association between suicide risk and these holidays, potentially guiding better prevention strategies.

## 2 Methods

### 2.1 Transparency and openness

The study protocol was registered with Open Science Framework (osf.io/7zx3d). We report our review using the Preferred Reporting Items for Systematic Reviews and Meta-Analyses guidance (PRISMA; see online Appendix 1). The study did not deviate from the pre-registered protocol.

### 2.2 Literature search

We searched four major databases, including PubMed, Embase, PsycInfo, and Cochrane Central Register of Controlled Trials (CENTRAL), with no limits applied (date, language), from database inception to 22 July 2024. We also searched the gray literature and reviewed reference lists of the included studies and related systematic reviews. The literature review was developed based on a preliminary review of the existing literature and consultation with topic and information specialists.

### 2.3 Study selection and outcomes

All articles with a potentially relevant title, abstract, or ones for which the relevance was unclear, were evaluated independently by two authors to determine inclusion eligibility. Discrepancies were resolved by deliberation between the two reviewers or with input from a third author. We excluded conference abstracts, case reports, case series, meta-analyses, and studies with duplicate data. Appendix 2 shows the complete search strategies, and Appendix 3 presents the reasons for exclusion. We defined the eligibility criteria for study inclusion based on the PICOS framework: Population (P): We included studies that reported on suicide deaths or self-harm/suicidal behaviors (SHSB) in the general population. No restrictions were applied regarding age, gender, or geographic location. Intervention/Exposure (I): The primary exposure of interest was the occurrence of specific calendar holidays, namely Christmas Eve, Christmas Day, New Year's Day, and Valentine's Day. Comparator (C): Comparator days were defined as non-holiday (regular) days, serving as a baseline reference for evaluating changes in suicide and SHSB rates. Outcomes (O): The primary outcomes were: (1) the proportion of suicide deaths on each holiday (defined as the number of suicides on the holiday divided by the total number of suicides in that year); (2) the risk of suicide (risk ratio or risk difference compared to regular days); and (3) the proportion and risk of SHSB on the specified holidays. SHSB was broadly defined to include suicide attempts, deliberate self-harm, and parasuicide, acknowledging diagnostic and definitional overlap in the literature. Study Design (S): We included observational studies (e.g., time-series, retrospective cohort, cross-sectional designs) that provided quantitative data on suicide or SHSB during at least one of the specified holidays and on non-holiday comparison days.

### 2.4 Data extraction and quality assessment

Two authors independently and in duplicate extracted data from selected articles. WebPlot Digitizer (https://automeris.io/WebPlotDigitizer/) was used to extract numerical data from the figures. We extracted the total number of suicides over the entire study duration, the average annual number of suicides, the average daily number of suicides, and the number of suicides on each of the four holidays. We also extracted the proportion of annual suicides that occur on each of the four holidays. The quality of the included studies was assessed using an outcome specific modified Newcastle-Ottawa scale. The Newcastle-Ottawa scale was selected for its ability to distinguish variations in quality based on number of confounders and covariates and its reliability compared with the ROBINS-I (Risk Of Bias In Non-randomized Studies—of Interventions) tool, The detailed data extraction is showed in Appendix 4.

### 2.5 Statistical analyses

All analyses were conducted in the statistical software R, version 4.2.0 (R core team, R Foundation for Statical Computing, Vienna, Austria) using the *metafor, ggpolt2, dplyr*, and *robumeta* R packages.

The proportion of annual suicides occurring on each of the four holidays was calculated as the number of suicides on that holiday divided by the total number of suicides in that year. We also calculated the risk ratio (RR) and risk difference (RD) for each of the four holidays compared to regular days (i.e., the average daily number of suicides over 365 days). Additionally, we calculated the proportion of annual SHSBs occurring on each of the four holidays, as well as the RR and RD of the four holidays compared to regular days.

For the proportion-based meta-analyses, the Freeman-Tukey double arcsine transformation was applied to stabilize variance. The pooled proportion with 95% confidence interval was calculated using the restricted maximum likelihood random-effects model with the inverse-variance weighting method. Cumulative proportion-based meta-analyses ordered by study year were also performed for RD and RR. We assessed inter-study heterogeneity with the Cochran Q test and quantified it by using the *I*^2^ statistic. For the Q statistic, we considered a *P*-value < 0.1 to be a statistically significant indicator of heterogeneity. *I*^2^ values considered representative of low, moderate, and high heterogeneity were < 25%, 26–50%, and >50%, respectively. We assessed publication bias by using funnel plot analysis, with evaluation of asymmetry by visual inspection followed by Egger's test. Tests were 2-tailed, and *P*-value < 0.05 was set as statistically significant.

### 2.6 Meta-regression and subgroup analysis

Where at least 10 studies were included in a meta-analysis, meta-regression explored heterogeneity by the following characteristics identified a priori: study design (national vs. local), continent, country, study duration, and study year (not publication year). Regarding holidays that did not conform to the expected proportions, subgroup analysis by country was conducted and presented the results using a world map.

### 2.7 Publication bias and sensitivity analyses

Publication bias or small study effects were examined using funnel plots and the Egger's test when 10 or more studies were included in a meta-analysis. The sensitivity analysis is to perform meta-analysis of non-affirmative studies and generalized linear mixed model with logit transformation for the primary outcome. The meta-analysis of non-affirmative studies assesses the robustness of our estimates and determines whether the pooled estimate would remain in the same direction even under the worst case publication bias.

## 3 Results

### 3.1 Study selection

After searching the databases and excluding duplicate records, we identified 586 unique, potentially eligible articles; 465 were excluded after title and abstract screening, and 93 were excluded after assessment of the full text (Supplementary material 3). Ultimately, 28 studies (*n* = 2,186,094) were included in our analysis, 18 for suicide deaths and 10 for SHSB (Supplementary Table 1). A PRSIMA flowchart depicting our search strategy is presented in Supplementary Figure 1. The follow-up duration of these studies were from 1 (27) to 35 (21) years. The earliest of study year is 1969 (21) and the most recent is 2019 (28), spanning a range of 50 years. There were 14 studies from Europe, 10 from the North America, two from Asia, one from South America, and one from Pacific.

### 3.2 Methodological quality of included studies

The quality assessment of the included studies were assessed with the Newcastle-Ottawa score (Supplementary Table 2) (29). Scores of 1, 2, and 3 indicate a high risk of bias, scores of 4, 5, and 6 indicate a moderate risk of bias, and scores of 7, 8, and 9 indicate a low risk of bias. There were 6 of 28 studies scoring 6 points (19, 23, 30–33), 8 studies scoring 7 points (17, 24, 27, 34–38), 12 studies scoring 8 points (18, 20–22, 28, 39–45), and 2 studies scoring 9 points (46, 47).

### 3.3 Suicide risk and proportion on Christmas Eve, Christmas Day, and New Year's Day

**Figure 1:**
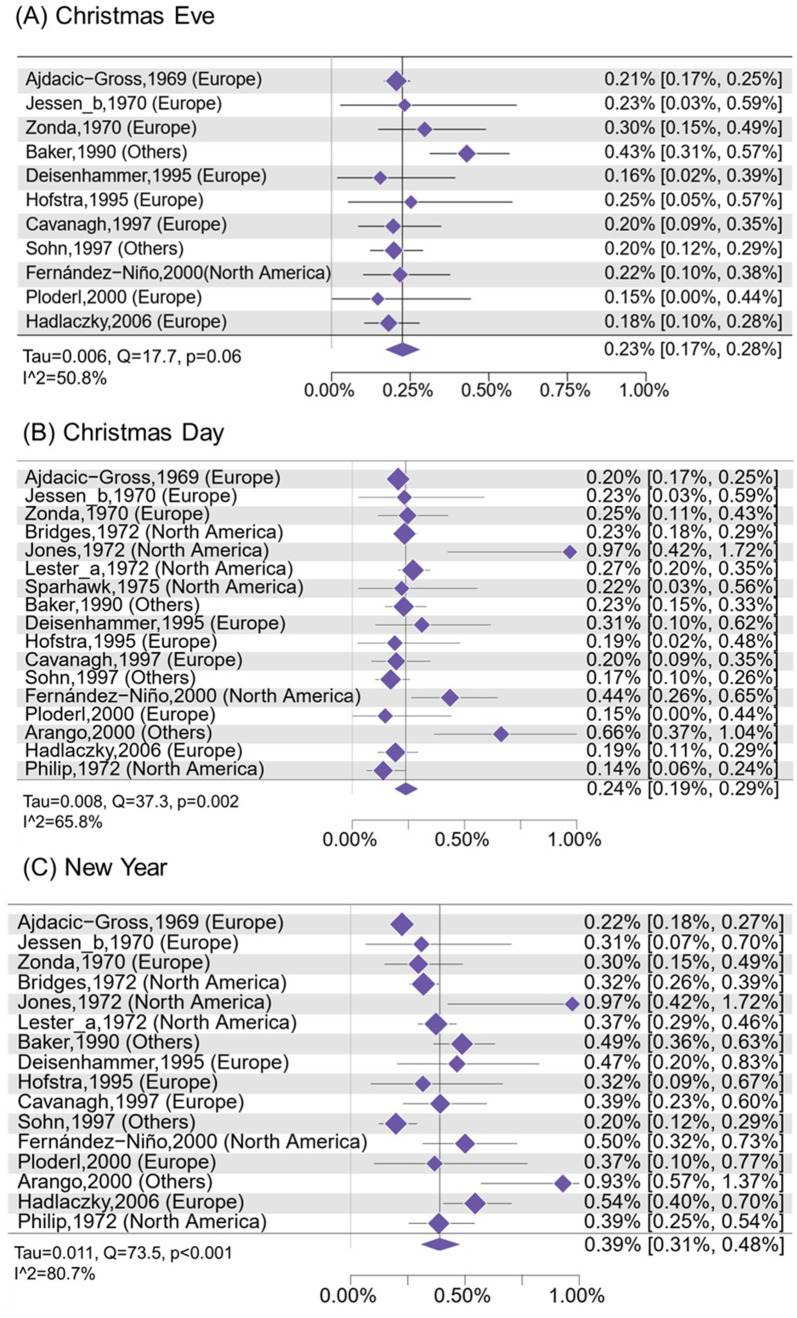
Proportion of annual suicides occurring on **(A)** Christmas Eve, **(B)** Christmas Day, and **(C)** New Year's Day.

**Figure 2:**
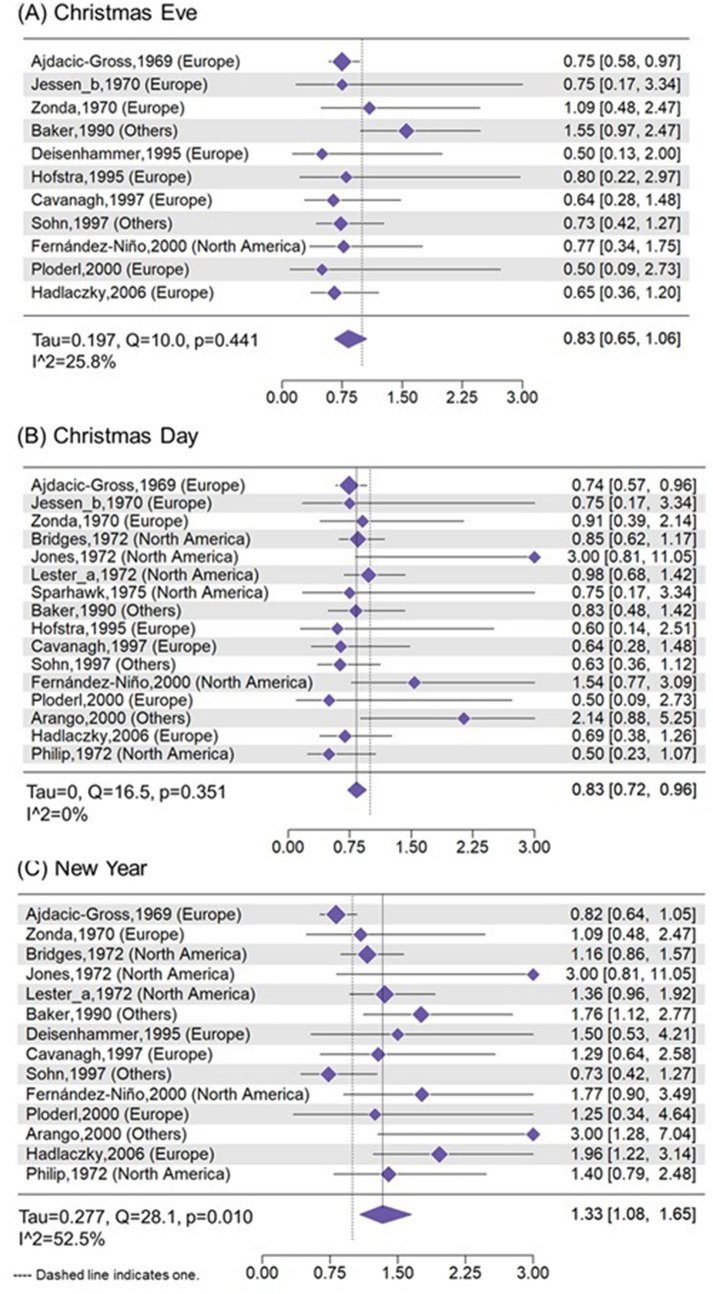
Risk ratio of suicides on **(A)** Christmas Eve, **(B)** Christmas Day, and **(C)** New Year's Day compared with that of regular day.

The proportion of annual suicides (Figure 1) was 0.23% [0.17%, 0.28%; number of studies (k) = 11; *I*^2^ = 50.8%] on Christmas Eve, 0.24% (0.19%, 0.29%; k = 17; *I*^2^ = 65.8%) on Christmas Day, and 0.39% (0.31%, 0.48%; k = 16; *I*^2^ = 80.7%) on New Year's Days, while it is 0.26% (Supplementary Figure 2, 0.24%, 0.29%; k = 17; *I*^2^ = 0%) on regular days (i.e., the average daily number of suicides over 365 days) (Figure 2). Compared with regular days, the risk was 17% lower on Christmas Day (RR = 0.83; 0.72, 0.96), and it was 33% higher (RR = 1.33; 1.08, 1.65) on New Year's Day. Additionally, the RD (Supplementary Figure 3) was significantly lower on Christmas Eve (−0.06%; −0.10%, −0.01%; k = 10; *I*^2^ = 0%), and it was significantly higher on New Year's Day (0.10%; 0.02%, 0.17%; k = 14; *I*^2^ = 60.7%) when compared with regular days. The proportion difference between these three holidays and regular days showed similar findings on meta-regression analysis (Supplementary Table 3).

**Figure 3:**
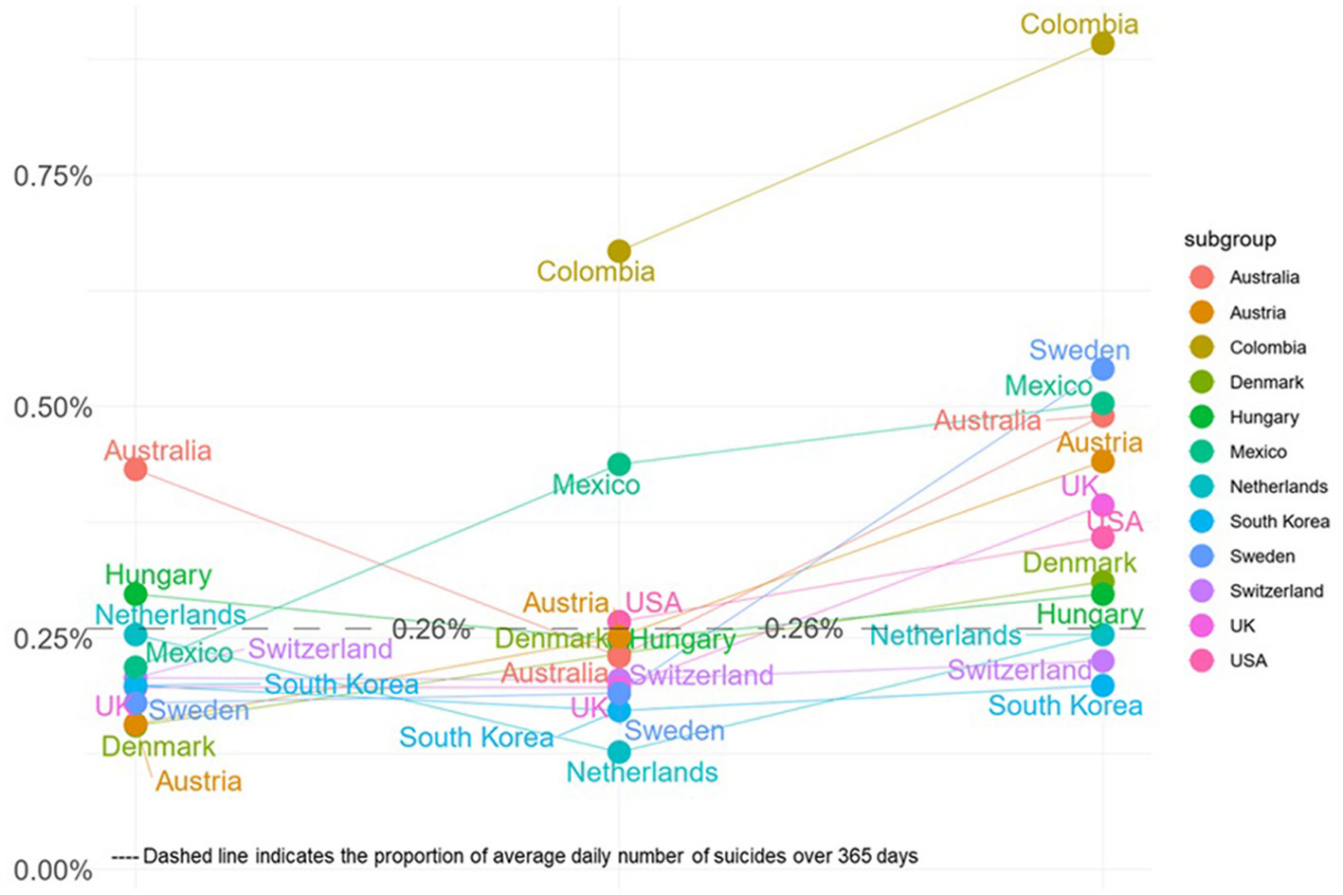
Temporal trend of suicide from Christmas Eve to New Year's Day by country.

The temporal trend of the proportion of annual suicides occurring from Christmas Eve to New Year Day are showed in Figure 3. Generally, most countries show a decreased or similar proportion of suicide deaths from Christmas Eve to Christmas Day, except for Mexico, and the largest decrease in the suicide proportion is seen in the Australia and Netherlands. However, most countries show an increased proportion of suicide after Christmas Day, leading up to New Year's Day.

The cumulative meta-analyses are in the Supplementary Data (Supplementary Figures 4–6). For Christmas Eve, the initial studies from 1969 to 1970 showed a lower proportion of suicide; however, after that, there was a sudden increase, but it gradually declined in the end. This temporal trend was similar on the Christmas Day. Importantly, the proportion of suicide on New Year's Day (Supplementary Figure 6) was low between 1969 and 1970, and after that, it is gradually increased, with more recent studies contributing to progressively higher estimates.

The meta-regression analyses (Supplementary Table 4) showed that the proportion of annual suicides occurring on Christmas Eve was lower on studies using national dataset (*p* < 0.001) and varied with different countries (*p* = 0.039). The proportion of annual suicides occurring on New Year's Day also varies with different countries (*p* < 0.001) and was lower on studies with longer duration (*p* = 0.009). The RD and RR of suicide on Christmas Eve and on New Year's Day compared with regular days showed similar findings with those of proportion of suicide (Supplementary Tables 5, 6). Besides, the RR of suicide on Christmas Day compared with regular days varies with different countries (Supplementary Table 5; *p* = 0.002).

### 3.4 Proportion of suicide, risk ratio, and risk difference on Valentine's Day

The proportion of annual suicides occurring on Valentine's Day (Supplementary Figure 7) was 0.27% (0.24%, 0.30%; k = 5; *I*^2^ = 0%), and the RR (Supplementary Figure 8) and RD (Supplementary Figure 9) were not significant compared with that on regular day. The cumulative meta-analysis did not show any temporal relationship (Supplementary Figure 10).

### 3.5 Proportion of suicide on Christmas Eve, Christmas Day, and New Year's Day by country

**Figure 4:**
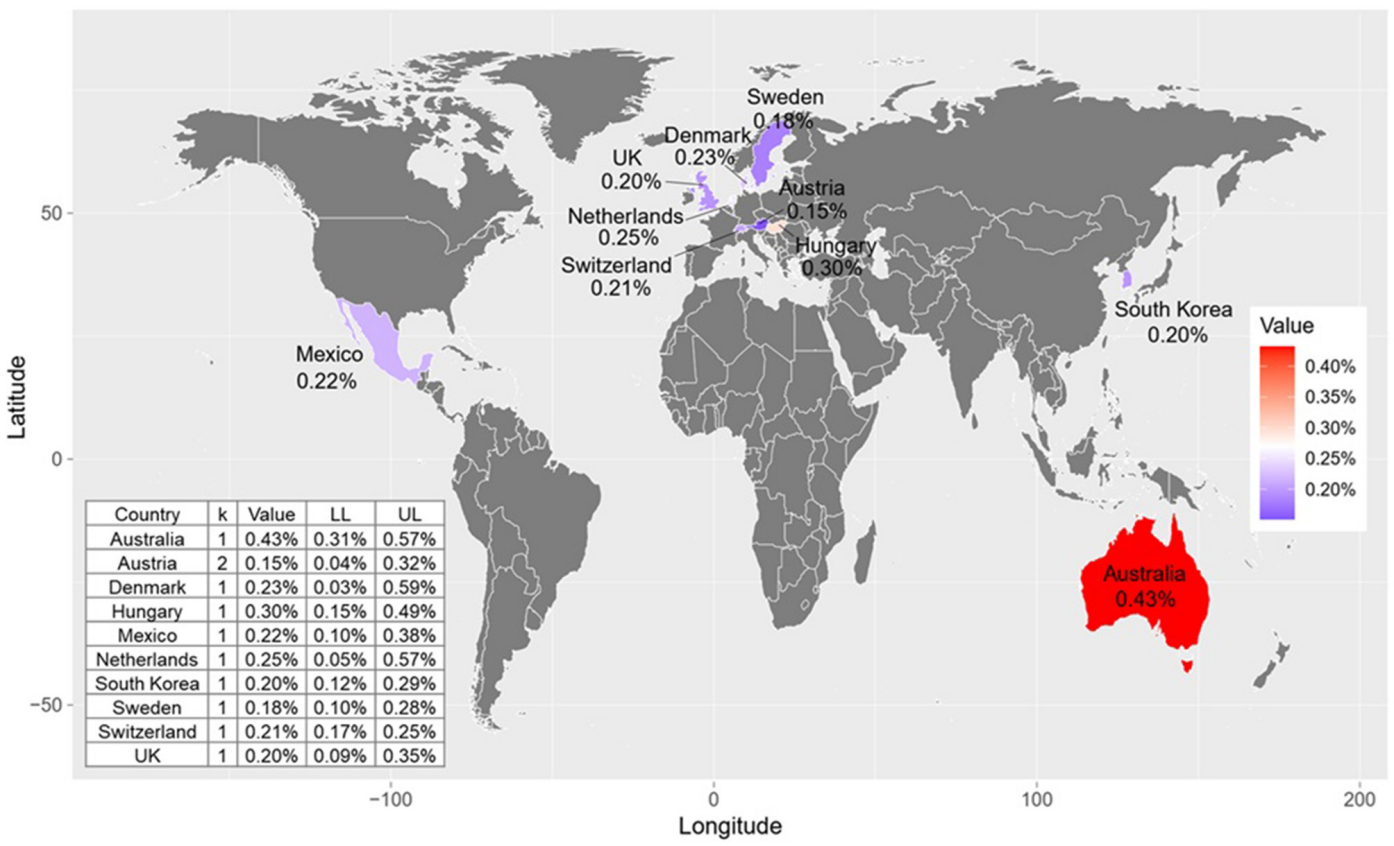
Proportion of annual suicides occurring on Christmas Eve by country.

**Figure 5:**
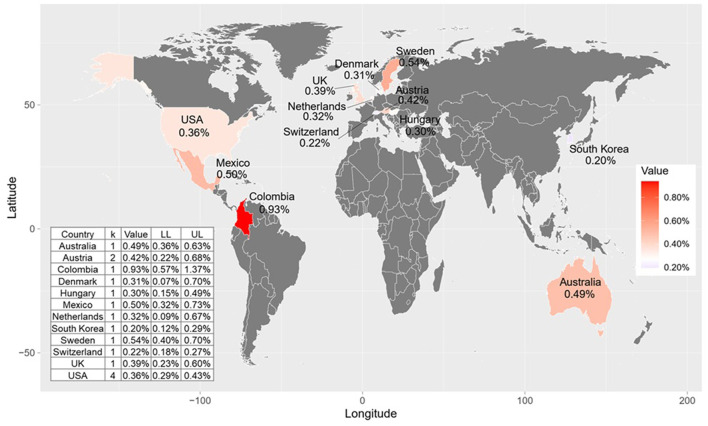
Proportion of annual suicides occurring on New Year's Day by country.

Stratified by country (Figure 4), most countries showed lower proportion of annual suicides occurring on Christmas Eve, except for Australia (0.43%; 0.31%, 0.57%) and Hungary (0.30%; 0.15%, 0.49%). Additionally, most countries showed lower proportion of annual suicides occurring on Christmas Day (Supplementary Figure 11), except for Colombia (0.66%; 0.37%, 1.04%) and Mexico (0.44%; 0.26%, 0.65%). For New Year's Day (Figure 5), the top three countries with the highest proportion of annual suicides are Columbia (0.93%; 0.57%, 1.37%), Sweden (0.54%; 0.40%, 0.70%), and Mexico (0.50%; 0.32%, 0.73%).

### 3.6 Proportion of suicide, risk ratio, and risk difference of SHSB on the four holidays

The proportion of annual SHSB occurring on Christmas Eve, Christmas Day, New Year's Day, and Valentine's Day are showed in the supplement (Supplementary Figures 12, 13). Compared with regular days, the risk of SHSB was 26% lower on Christmas Eve (RR = 0.74; 0.57, 0.96; Supplementary Figure 14), and it was 17% higher on New Year's Day (RR = 1.17; 1.03, 1.34). However, the RR of SHSB was not significant on Valentine's day compared with regular days (Supplementary Figure 15). The RD of SHSB (Supplementary Figures 16, 17) showed similar findings as those of suicide deaths.

### 3.7 Publication bias

Visual inspection of the funnel plots for the primary outcomes did not show significant publication bias (Supplementary Figures 18–29). However, the Egger test was significant for proportion of annual suicides occurring on New Year's Day (Supplementary Figure 20; *p* = 0.011) and RD of suicide (Supplementary Figure 24; *p* = 0.004) on New Year's Day compared with regular days. We also conducted trim-and-fill analysis for potential publication bias (Supplementary Figures 30–33). For Christmas' eve (Supplementary Figure 30) and Valentine's day (Supplementary Figure 33), the trim-and-fill analysis identified two potentially missing studies for each analysis, but the adjusted overall effect size remained similar to the original estimate (PFT = 0.05, 95% CI: 0.04–0.05) and (PFT = 0.05, 95% CI: 0.05–0.05). The funnel plot showed slight asymmetry, suggesting minimal publication bias without materially altering the pooled results. For Christmas day (Supplementary Figure 31) and New Year's Day (Supplementary Figure 32), the pooled analysis yielded an overall proportion of 0.05 (95% CI: 0.05–0.06) and 0.06 (95% CI: 0.06–0.07) across all studies. The funnel plots revealed a largely symmetrical distribution, indicating minimal risk of publication bias. No additional studies were imputed by the trim-and-fill procedure, suggesting the robustness of the pooled estimate.

### 3.8 Meta-analysis of non-affirmative studies and sensitivity analysis

When including non-affirmative studies (Supplementary Table 7), the direction of pooled estimates of RR and RD of suicide deaths showed consistent direction with those of original analyses on the four holidays. The generalized linear mixed model with logit transformation (Supplementary Table 8) showed similar findings on the proportion of suicide on the four holidays.

### 3.9 Leave-one-out analyses

Supplementary Tables 9–12 demonstrated leave-one-out analyses for proportion of annual suicides occurring on Christmas Eve, Christmas Day, New Year's Day, and Valentine's day. For Christmas Eve, after removing Baker et al. (22), the I^2^ would decline from 50.8% to 0%, indicating this study was the source of heterogeneity. For Christmas Day, after removing Jones et al. (36), Fernández-Niño et al. (34), and Arango et al. (40), the I^2^ would decline from 65.8% to 47.0%, 46.7%, and 32.7%, suggesting these three studies contributed slightly more to heterogeneity. For New Year's Day, leave-one-out sensitivity analyses showed that removing any single study produced minimal changes in the pooled estimates (0.0022–0.0025), with all results remaining statistically significant (p < 0.001). Moderate heterogeneity (*I*^2^ = 60–70%) persisted across all scenarios, and no single study substantially reduced it. For Valentine's day, removing any single study kept the *I*^2^ at 0, indicating high homogeneity.

## 4 Discussion

The association between major holidays and suicide is not entirely consistent; some holidays are positively associated with increased suicidal, some are negatively associated, and others show no significant difference compared to regular days. In our study, we found that Christmas Eve and Christmas Day were associated with a lower risk of suicide than that on regular day. However, just1week after Christmas, the risk of suicide was sharply increased on New Year's Day. The temporal trend of suicide risk from Christmas to New Year's Day was observed across countries. Indeed, we found that country is a significant moderator for the risk of suicide on Christmas Eve, Christmas Day, and New Year's Day, with the degree of change varying across different nations. For the risk of SHSB, we also found a lower risk on Christmas Eve and Christmas Day and a higher risk on New Year's Day compared with that on regular day. Finally, no statistically significant difference in the risk of suicide and SHSB was observed between Valentine's Day and regular days. In brief, although both Christmas and New Year's Day are major holidays that emphasize family and social connections, only New Year's Day act as a temporal hotspot of suicide across most countries.

There could be both positive and negative effects on the association between major holiday and suicide risk. For instance, Christmas and New Year's Day can generate feelings of hope and optimism; however, they may also bring about psychosocial broken-promise effect (13, 14), leading to anxiety, distress, depression, and even hopelessness and helplessness. In the current study, we found a lower risk of suicide on Christmas Eve and Christmas Day compared with that on regular day, with few heterogeneities. This finding is consistent with those of previous studies (15, 48). The lower suicide rates observed during Christmas Eve and Christmas Day might be attributed to the positive emotional expectations linked with the holiday season, such as increased feelings of hope. Additionally, enhanced social connectedness and support during this period is also considered (43, 48). However, the tendency for individuals contemplating suicide to delay their actions until after Christmas could be another important factor (43, 48). Such delay in suicide tendency might result in a peak of suicide on New Year's Day. Indeed, among the included 12 countries, we found that almost all countries showed an increased risk of suicide on New Year's Day, except for South Korea and Switzerland. Besides, the cumulative meta-analysis showed more recent studies contributing to progressively higher estimates of suicide risk on New Year's Day. This temporal trend may reflect broader societal changes, such as increasing economic pressures, heightened social expectations, or evolving patterns of holiday-related alcohol consumption, which could exacerbate vulnerability during this period (49–51). The review conducted by Plöderl et al. also reported an increase in the suicide rate after Christmas, but on New Year's Day, it only returned to the yearly average (48). Another point needs to be considered is a delay between the time of death and its confirmation. Since many hospitals are closed on holidays, this may affect how quickly a death is confirmed, and the record of death might be delayed (52).

In sensitivity analyses, we found that four studies, including Baker et al. (22). (Christmas Eve), Jones et al. (36) (Christmas Day), Fernández-Niño et al. (34) (Christmas Day), and Arango et al. (40) (Christmas Day), might contribute to heterogeneity, and all of them reported relatively higher suicide rates. The relatively higher suicide rates reported by Fernández-Niño et al. (34) (Mexico) and Arango et al. (40) (Colombia) during the Christmas period may reflect unique cultural and contextual factors in Latin American countries. Christmas in many Latin American cultures is a highly family-centered holiday with strong religious and social expectations. For individuals experiencing social isolation, economic hardship, or family conflict, the contrast between festive norms and personal circumstances may exacerbate feelings of loneliness or distress. Both Jones et al. (36) and Baker et al. (22) used local suicide registers, which often report higher suicide rates than national registries. This discrepancy may arise because local databases typically have more complete case ascertainment and less underreporting, whereas national registries may miss cases due to variations in reporting standards or data collection systems.

For Valentine's day, there were five studies reporting suicide deaths and five reporting SHSB. The five studies reported that the suicide risk on Valentine's Day did not differ from than that on regular day with few heterogeneities. However, when estimating SHSB, there was high heterogeneity across the five studies. Three studies indicated a higher risk of SHSB on Valentine's Day (23, 32, 33). Valentine's Day is a holiday particularly valued by adolescents and young adults, and it is typically a celebration of romantic relationships. Valentines' day can also be a time of mourning loss or the absence of a romantic partner. This age group is more likely to engage in parasuicidal behavior when facing relationship problems (53). The study showing the highest risk of SHSB, conducted by Davenport et al., reported that the median age of individuals who did parasuicide on Valentine's Day was 21 years (23). However, as only five studies analyzed suicide on Valentine's Day, the statistical power to detect small effects may be limited. Therefore, the absence of a statistically significant difference should be interpreted with caution, and further research is warranted to draw more definitive conclusions.

An important question is whether there are positive emotional expectations before major holidays that reduce the suicide risk in the days leading up to these holidays. Indeed, several studies have assessed the differences in suicide risk on holidays, before holidays, and after holidays (19, 22, 33, 45). However, when looking at the continuous trend of suicide rates before and after holidays, the peak (whether high or low) still occurred on the holiday itself. Although the number of studies we included is insufficient for analyzing the 3 days before and after Christmas or the week before and after, we can at least observe a trend of decreasing suicide risk from Christmas Eve to Christmas.

### 4.1 Strengths and limitations

Our study has several strengths. We calculated the annual mean proportion of the number of suicide on the major holiday as primary outcomes. In this way, we can avoid the fluctuations in suicide rates throughout the year and overcome the different suicide tendency in different country/study by comparing only with the annual average suicide proportion. Besides, we also calculated RD and RR to represent both absolute and relative risk difference between major holidays and regular day. Previous reviews (15, 48) investigating suicide risks during Christmas/New Year have only conducted systematic reviews due to the heterogeneity in study designs or humanities. We conducted the first meta-analysis and utilized sensitivity tests (including subgroup analysis, meta-regression, and meta-analysis of non-affirmative studies) to further validate our findings.

Our study has several limitations. First, most of the included studies originate from developed countries and predominantly Christian populations, which limits the ability to generalize the findings to developing and least developed countries. In different cultures and regions, the importance of Christmas and New Year to people may vary. In countries where Christianity is not the predominant religion, the suicide risk during Christmas may be similar to that on regular days; however, certain holidays or cultural events in other communities may also be associated with decreased suicidal tendencies, warranting further investigation. Second, we overcame some difficulties by using the annual mean proportion of the number of suicides to calculate the suicide proportion and risk. However, the changes in suicide rates over the years might not be considered, particularly a very long follow-up period. We employed meta-regression to examine whether the study year and the study duration are important moderators, showing no or trivial effect. Third, the included studies span from the 1970s to the 2010s. Over these 40–50 years, environmental factors and suicide mortality rates have also changed in different countries. Therefore, in addition to meta-regression, we also conducted cumulative meta-analyses ordered by study year. We found that the risk of suicide on New Year's Day gradually increased, with more recent studies contributing to progressively higher estimates. Fourth, in the epidemiology of suicide, factors such as age, gender, socioeconomic status, substance use, prior suicidal behavior, and alcohol use are important. However, few included studies provided data on their association with suicide on major holidays, and most also lacked detailed information on suicide methods, limiting further analysis of these risk factors. Fifth, when calculating SHSB, it is not possible to distinguish between suicide attempts accompanied by some deaths or self-harm/parasuicide without death. Therefore, there is a certain degree of heterogeneity in the results of SHSB. Sixth, we found small study effects on the risk of suicide on New Year's Day. However, the meta-analyses of non-affirmative studies suggest the robustness of our study findings. Finally, in South Korea (20), Taiwan, and China, Lunar New Year might be considered a more important new year compared to Solar New Year. However, the data on Lunar New Year's Day were not available.

## 5 Conclusions

Although socio-cultural factors and datasets vary among the studies, most countries show a relatively lower risk of suicide on Christmas Eve and Christmas Day. However, just 1 week after Christmas, the risk of suicide was significantly increased on New Year's Day. In many countries, Christmas and New Year's Day often constitute a continuous holiday period during which healthcare, psychological, emergency, and social workers are often understaffed. Our findings underscore the importance of strengthening suicide prevention efforts during these critical periods. Governments and local health authorities should not only enhance emergency department preparedness but also implement comprehensive suicide prevention programs, ensuring adequate deployment of mental health professionals, crisis intervention services, and preventive warning signs and resource in advance. to mitigate the heightened suicide risk around New Year's Day.

## Data Availability

The original contributions presented in the study are included in the article/Supplementary material, further inquiries can be directed to the corresponding authors.
