# Supplementary material for: Suicide risk on Christmas Eve, Christmas Day, New Year's Day, and Valentine's Day: a systematic review and meta-analysis
Source: Front Public Health. 2025 Sep 30;13:1668476. doi: 10.3389/fpubh.2025.1668476 (PMC12518309; doi:10.3389/fpubh.2025.1668476)
Supplement: Supplementary file 1 [file Table_1.docx]

**Suicide Risk on Christmas Eve, Christmas Day, New Year’s Day, and Valentine’s Day: A Systematic Review and Meta-analysis**

**eFigure 1.** PRISMA 2020 flow diagram for new systematic reviews which included searches of databases and registers only.

**eFigure 2.** Proportion of Average Daily Number of Suicides over 365 Days

**eFigure 3.** Risk Difference of Death by Suicide on Christmas Eve, Christmas Day, and New Year’s Day compare with regular days

**eFigure 4.** Cumulative Meta-analysis of Proportion of Annual Suicides Occurring on Christmas Eve

**eFigure 5**. Cumulative Meta-analysis of Proportion of Annual Suicides Occurring on Christmas Day

**eFigure 6.** Cumulative Meta-analysis of Proportion of Annual Suicides Occurring on New Year’s Day

**eFigure 7.** Proportion of Annual Suicides Occurring on Valentine’s Day

**eFigure 8.** Risk Ratio of Death by Suicide on Valentine’s Day Compared with Regular Days

**eFigure 9.** Risk Difference of Death by Suicide on Valentine’s Day Compared with Regular days

**eFigure 10.** Cumulative Meta-analysis of Proportion of Annual Suicides Occurring on Valentine’s Day

**eFigure 11.** Proportion of Annual Suicides Occurring on Christmas Day by Country

**eFigure 12.** Proportion of Annual Self-Harm and Suicidal Behaviors Occurring on Christmas Eve, Christmas Day, and New Year’s Day

**eFigure 13.** Proportion of Self-Harm and Suicidal Behaviors on Valentine’s Day over a One-Year Period

**eFigure 14.** Risk Ratio of Self-Harm and Suicidal Behaviors on Christmas Eve, Christmas Day, and New Year’s Day Compared with Regular Day

**eFigure 15.** Risk Ratio of Self-Harm and Suicidal Behaviors on Valentine’s Day Compared with Regular Day

**eFigure 16.** Risk Difference of Self-Harm and Suicidal Behaviors on Christmas Eve, Christmas Day, and New Year’s Day Compared with Regular Day

**eFigure 17.** Risk Difference of Self-Harm and Suicidal Behaviors on Valentine’s Day Compared with Regular Day

**eFigure 18.** Funnel Plot with Egger Test for the Proportion of Suicide on Christmas Even over a One-Year Period

**eFigure 19.** Funnel Plot with Egger Test for the Proportion of Suicide on Christmas Day over a One-Year Period

**eFigure 20.** Funnel Plot with Egger Test for the Proportion of Suicide on New Year’s Day over a One-Year Period

**eFigure 21.** Funnel Plot with Egger Test for the Proportion of Suicide on Valentine’s Day over a One-Year Period

**eFigure 22.** Funnel Plot with Egger Test for the Risk Difference of Suicide on Christmas Eve vs Regular Day

**eFigure 23.** Funnel Plot with Egger Test for the Risk Difference of Suicide on Christmas Day vs Regular Day

**eFigure 24.** Funnel Plot with Egger Test for the Risk Difference of Suicide on New Year’s Day vs Regular Day

**eFigure 25.** Funnel Plot with Egger Test for the Risk Difference of Suicide on Valentine’s Day vs Regular Day

**eFigure 26.** Funnel Plot with Egger Test for the Risk Ratio of Suicide on Christmas Eve vs Regular Day

**eFigure 27.** Funnel Plot with Egger Test for the Risk Ratio of Suicide on Christmas Day vs Regular Day

**eFigure 28.** Funnel Plot with Egger Test for the Risk Ratio of Suicide on New Year’s Day vs Regular Day

**eFigure 29.** Funnel Plot with Egger Test for the Risk Ratio of Suicide on Valentine’s Day vs Regular Day

**eFigure 30**. Trim-and-Fill sensitivity analysis for the Christmas eve

**eFigure 31.** Trim-and-Fill sensitivity analysis for the Christmas day

**eFigure 32.** Trim-and-Fill sensitivity analysis for the New Year

**eFigure 33.** Trim-and-Fill sensitivity analysis for the Valentine day

**eTable 1.** The characteristics and demographics of the included studies.

**eTable 2.** Risk of bias of included reports from studies as assessed with the Newcastle-Ottawa Scale

**eTable 3.** Meta-regression of Proportion of Annual Suicides Occurring on Christmas Eve, Christmas Day, and NewYear's Day, and Valentine’s Day Compared with Regular Days

**eTable 4.** Meta-regression of Proportion of Annual Suicides Occurring on Holiday

**eTable 5.** Meta-regression of Risk Ratio (Compared with Regular Days)

**eTable 6.** Meta-regression of Risk Difference (Compared with Regular Days)

**eTable 7.** Meta-analysis of Non-affirmative Studies

**eTable 8.** Meta-analysis using Generalised Linear Mixed Model with Logit Transformation

**eTable 9.** Leave-one-out sensitivity analysis for the Christmas eve

**eTable 10**. Leave-one-out sensitivity analysis for the Christmas day

**eTable 11.** Leave-one-out sensitivity analysis for the New year

**eTable 12.** Leave-one-out sensitivity analysis for the Valentine day

**Appendix 1.** Checklist of PRISMA guideline

**Appendix 2.** Search Strategy

**Appendix 3.** Reasons for Exclusion

**Appendix 4.** Details of Data Extraction on the Number of Suicides During Holidays and Regular Days

eFigure 1. PRISMA 2020 flow diagram for new systematic reviews which included searches of databases and registers only.

**Identification of studies via databases and registers**

Records identified from

Embase (n= 345)

PubMed (n= 163)

CENTRAL (n= 110)

PsycINFO (n= 94):

Databases (n =712)

Records removed *before screening*: Duplicate records removed (n = 126)

**Identification**

Records excluded title and abstract (n = 465)

Records screened

(n = 586)

Reports not retrieved

(n = 0)

Reports sought for retrieval

(n = 121)

**Screening**

**Reports excluded:**

Conference abstract and review (n =11)

Duplicated database (n =2)

No outcome of interest (n =74)

No available full-text (n=6)

Reports assessed for eligibility

(n = 121)

Studies included in review

(n =28)

Reports of included studies

(n =28)

**Included**

*From:*  Page MJ, McKenzie JE, Bossuyt PM, Boutron I, Hoffmann TC, Mulrow CD, et al. The PRISMA 2020 statement: an updated guideline for reporting systematic reviews. BMJ 2021;372:n71. doi: 10.1136/bmj.n71

eFigure 2. Proportion of Average Daily Number of Suicides over 365 Days
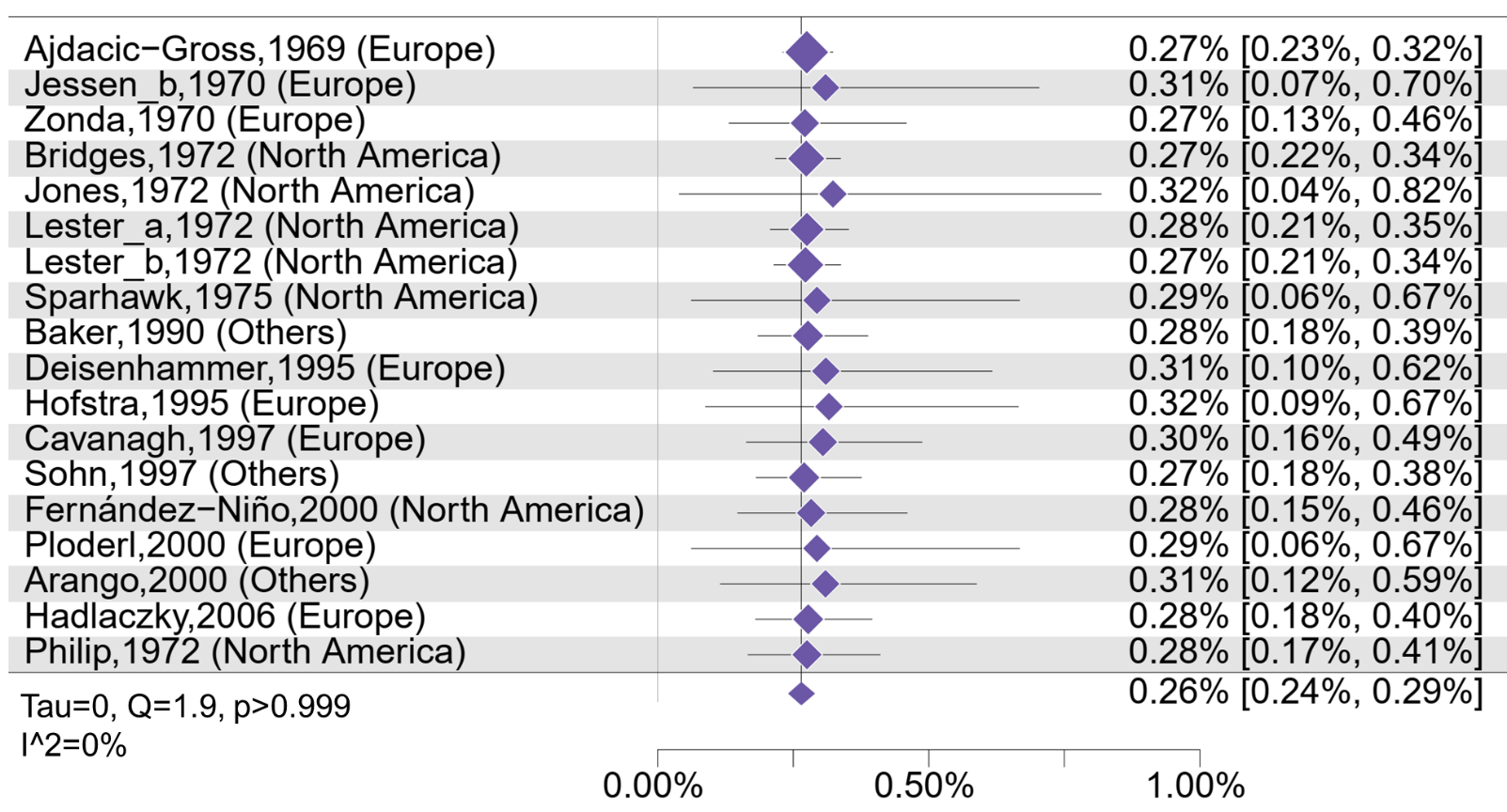


eFigure 3. Risk Difference of Death by Suicide on Christmas Eve, Christmas Day, and New Year’s Day compare with regular days


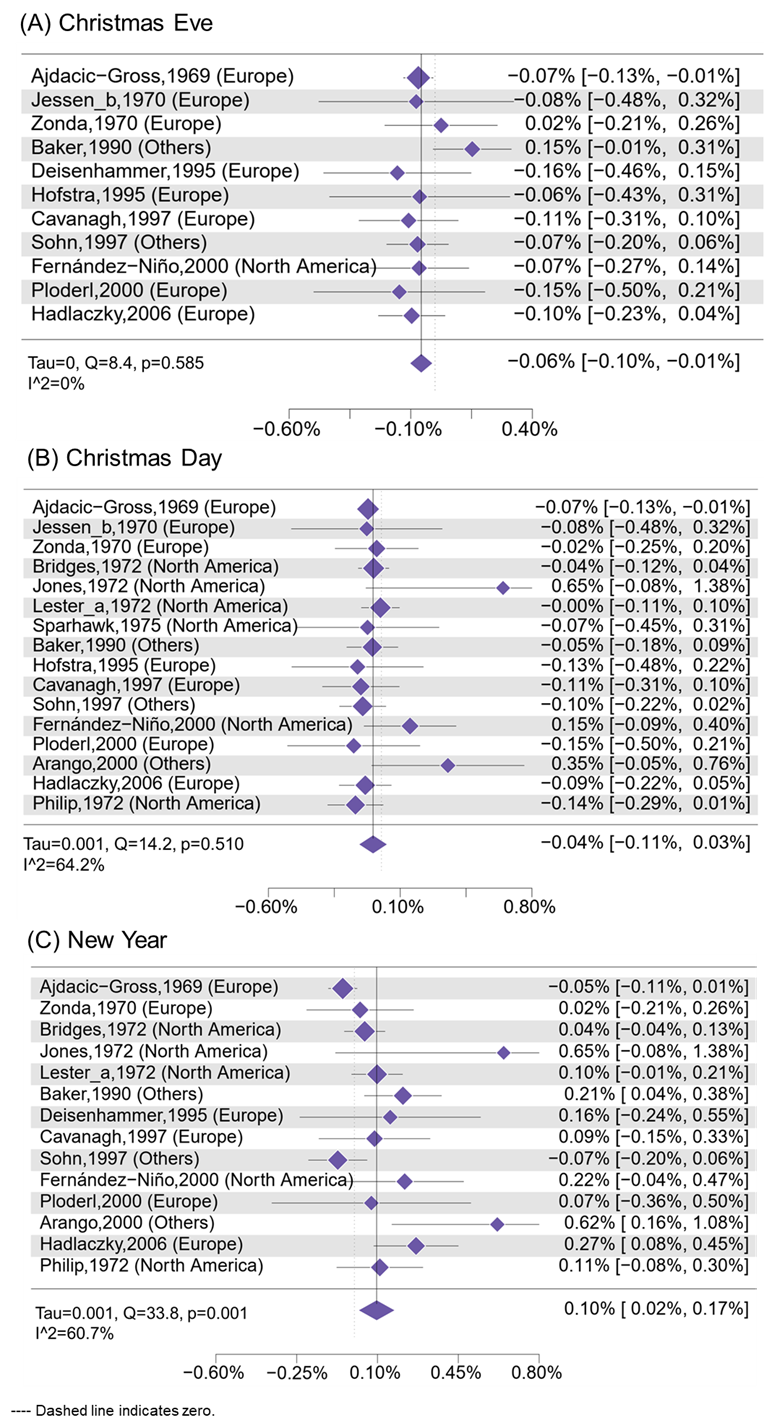


eFigure 4. Cumulative Meta-analysis of Proportion of Annual Suicides Occurring on Christmas Eve


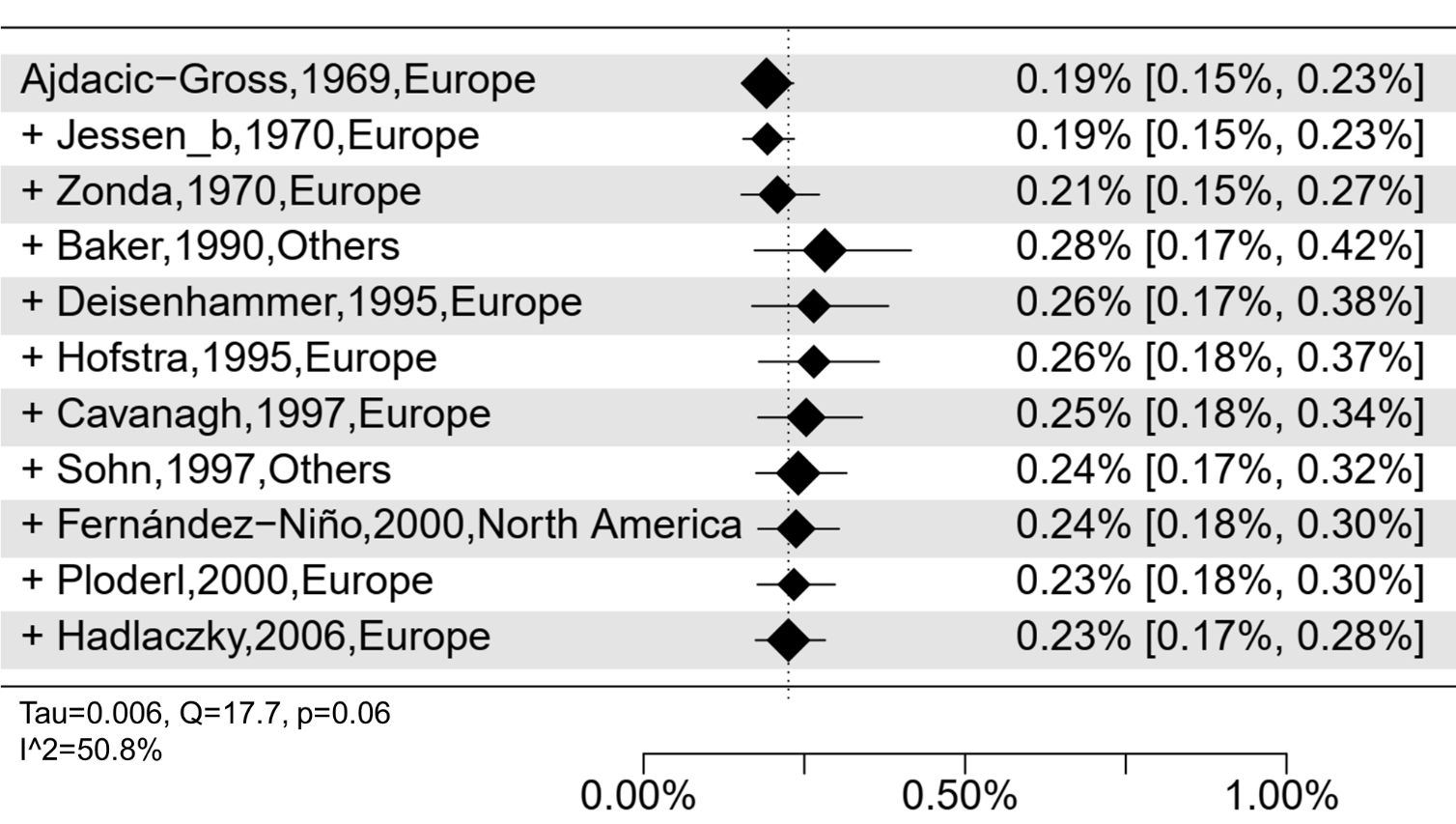


eFigure 5. Cumulative Meta-analysis of Proportion of Annual Suicides Occurring on Christmas Day


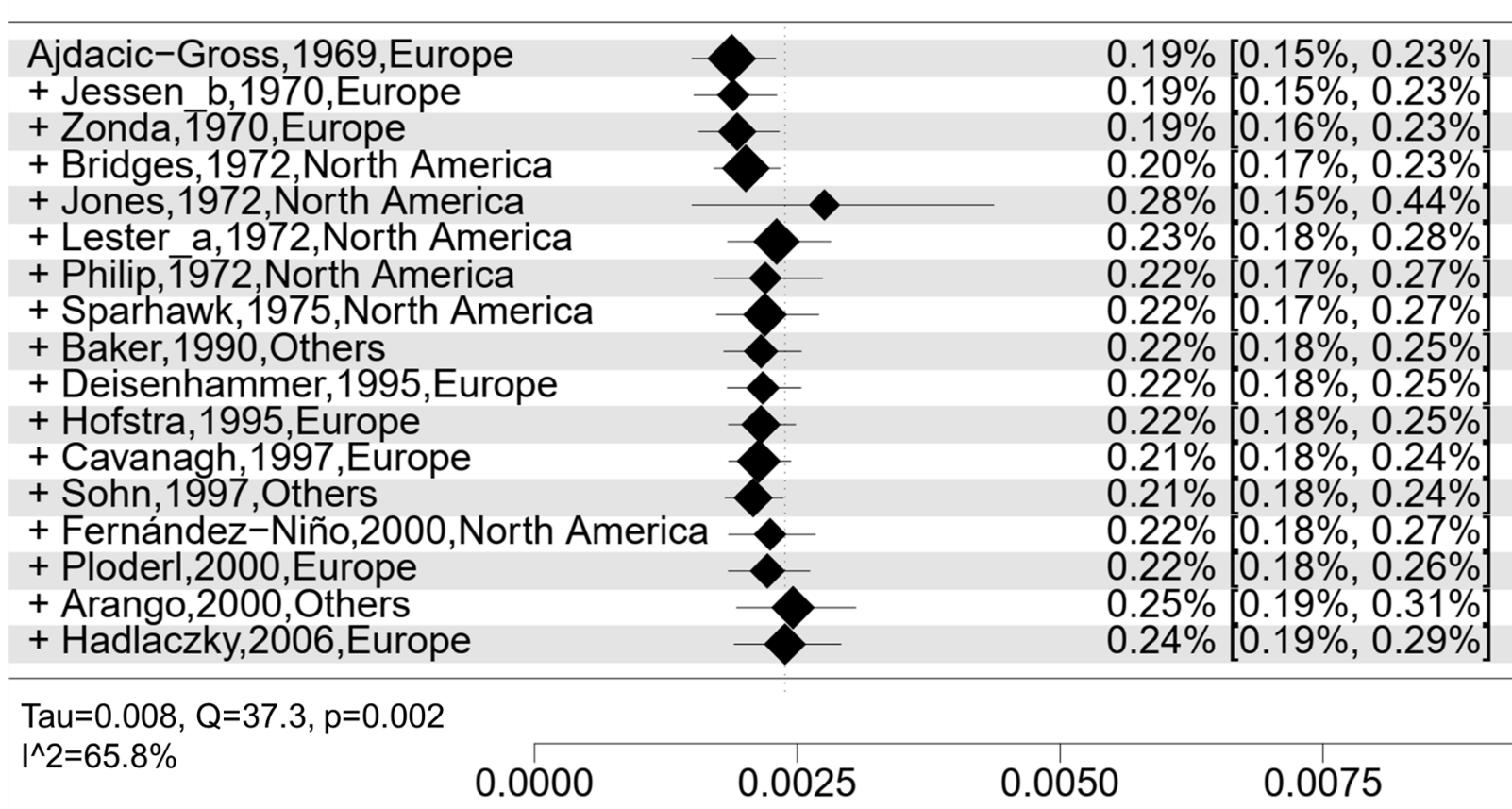


eFigure 6. Cumulative Meta-analysis of Proportion of Annual Suicides Occurring on New Year’s Day


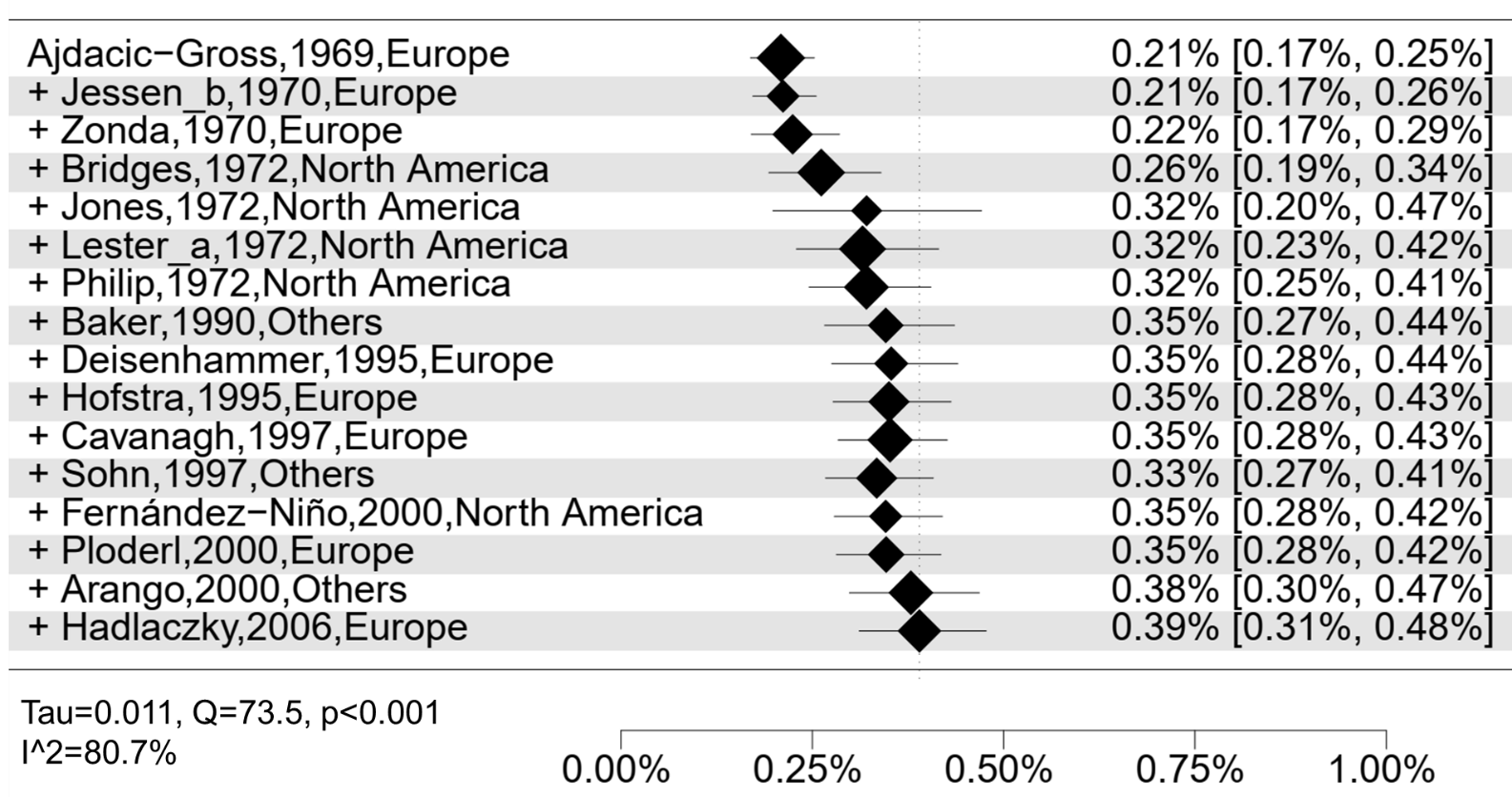


eFigure 7. Proportion of Annual Suicides Occurring on Valentine’s Day


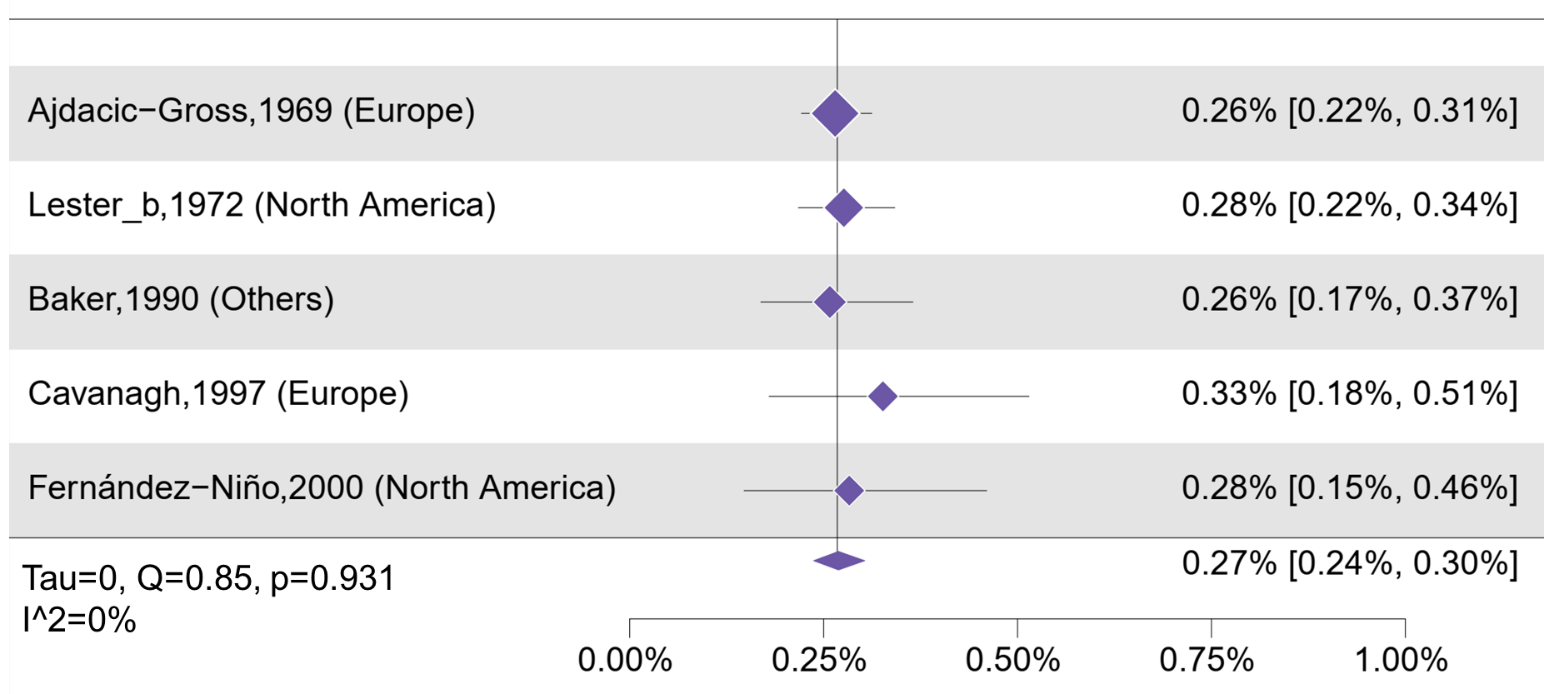


eFigure 8. Risk Ratio of Death by Suicide on Valentine’s Day Compared with Regular Days


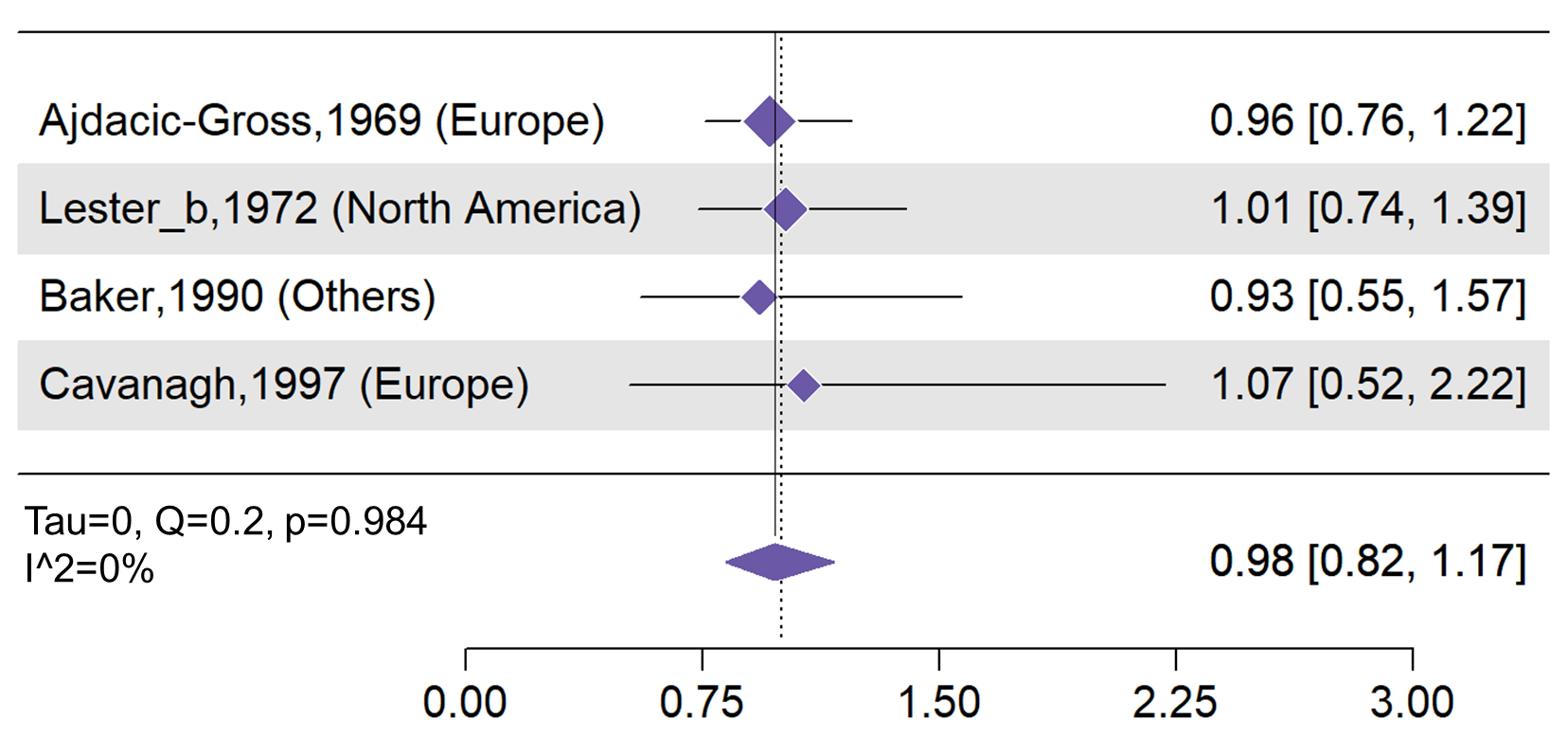


eFigure 9. Risk Difference of Death by Suicide on Valentine’s Day Compared with Regular days


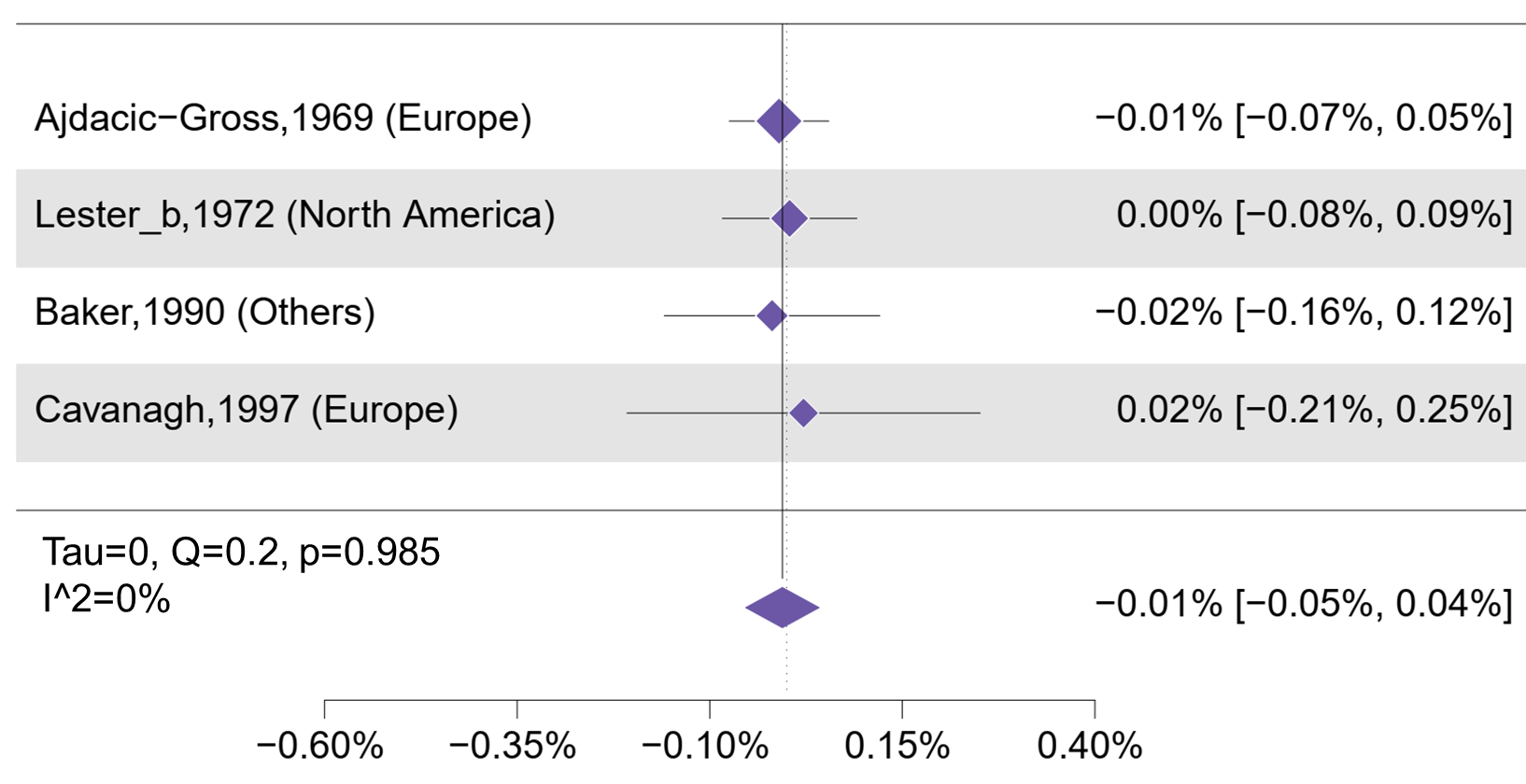


eFigure 10. Cumulative Meta-analysis of Proportion of Annual Suicides Occurring on Valentine’s Day


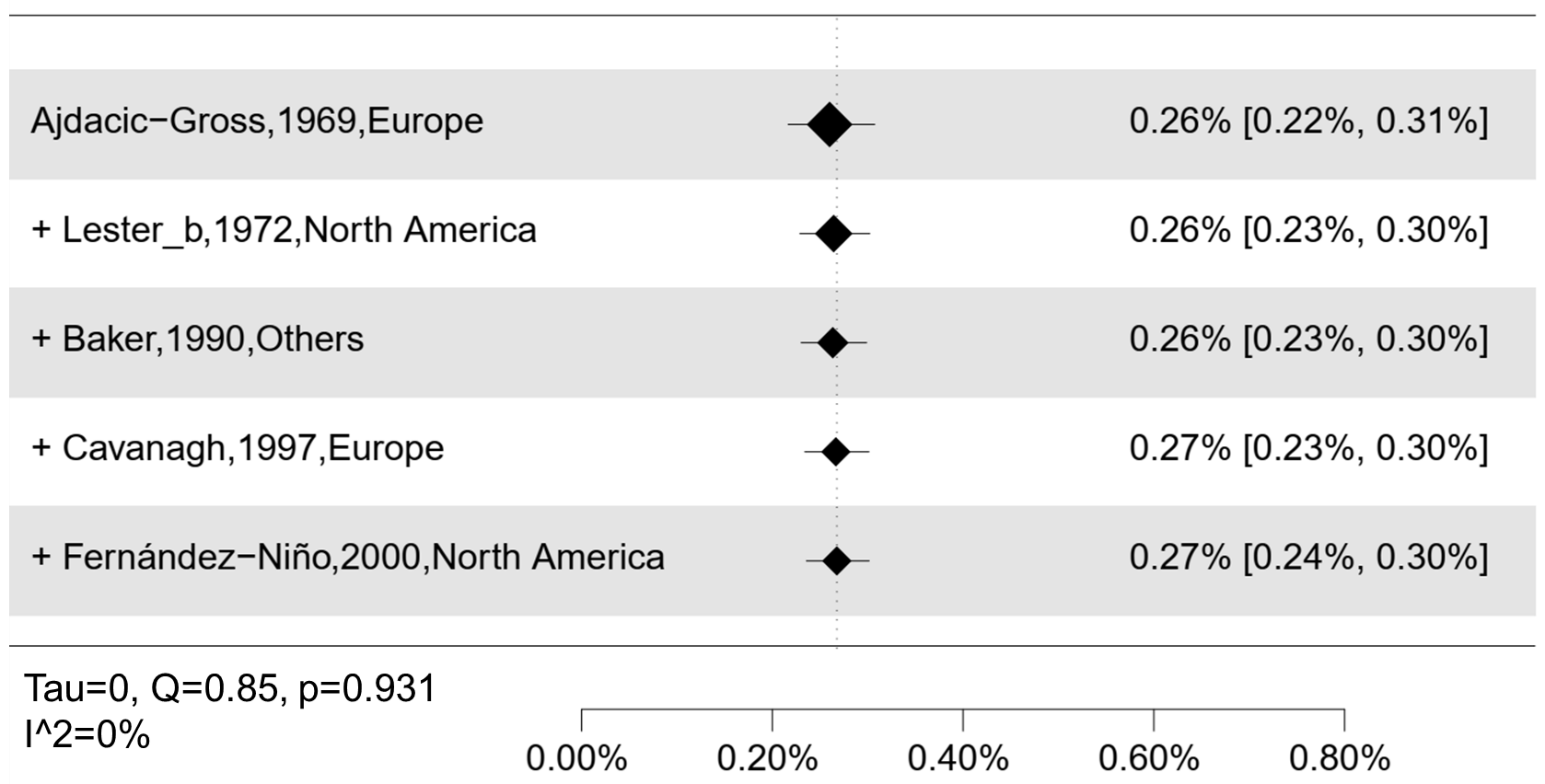


eFigure 11. Proportion of Annual Suicides Occurring on Christmas Day by Country


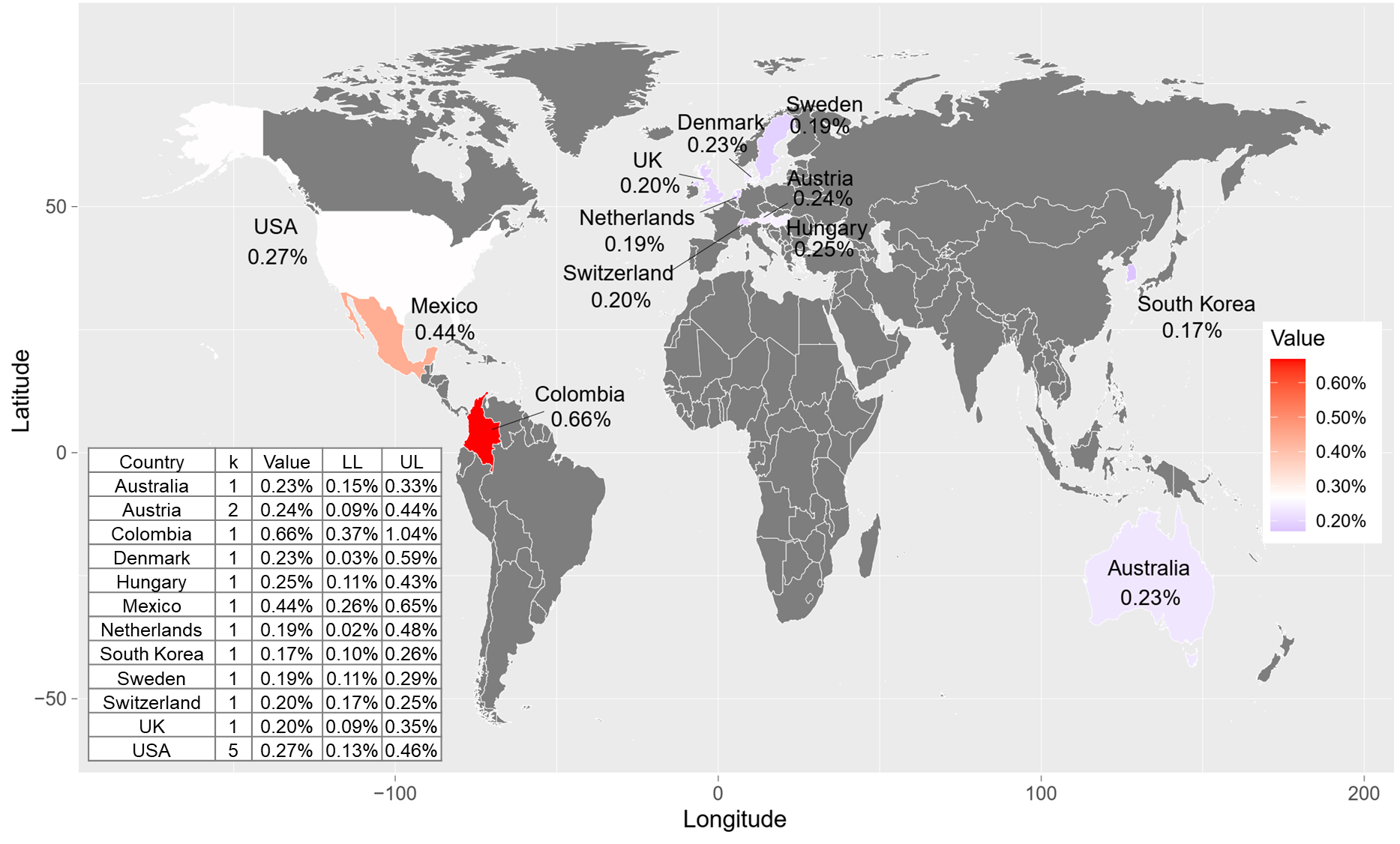


eFigure 12. Proportion of Annual Self-Harm and Suicidal Behaviors Occurring on Christmas Eve, Christmas Day, and New Year’s Day


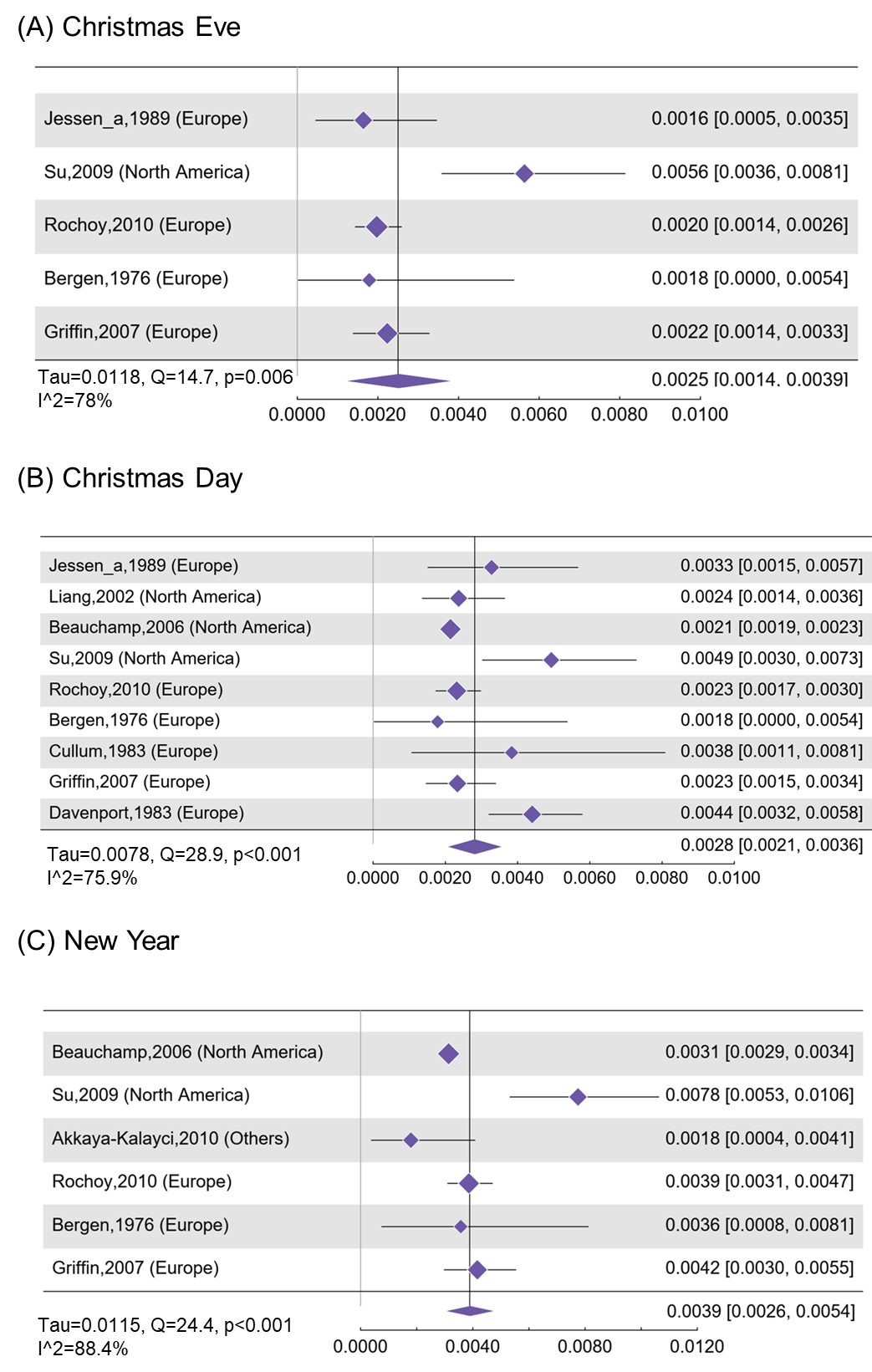


eFigure 13. Proportion of Self-Harm and Suicidal Behaviors on Valentine’s Day over a One-Year Period


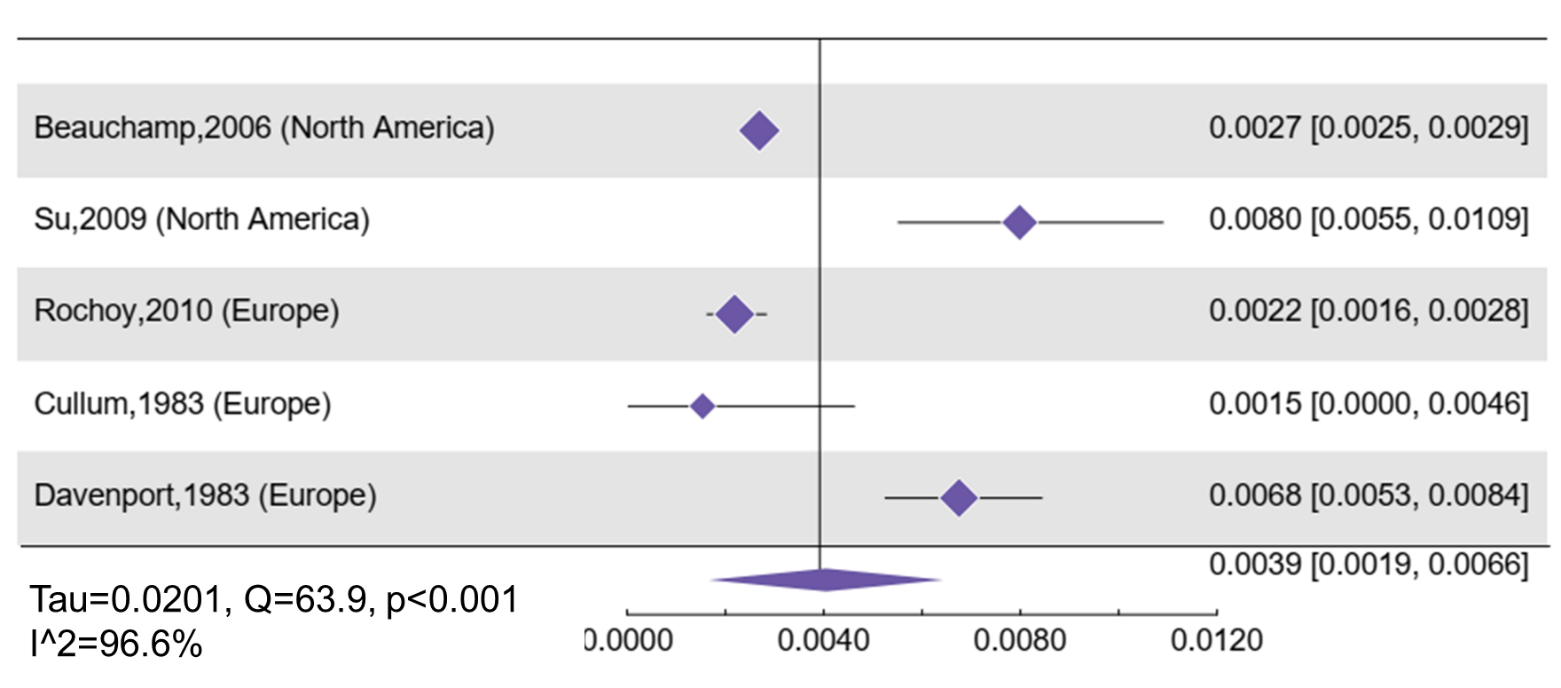


eFigure 14. Risk Ratio of Self-Harm and Suicidal Behaviors on Christmas Eve, Christmas Day, and New Year’s Day Compared with Regular Day


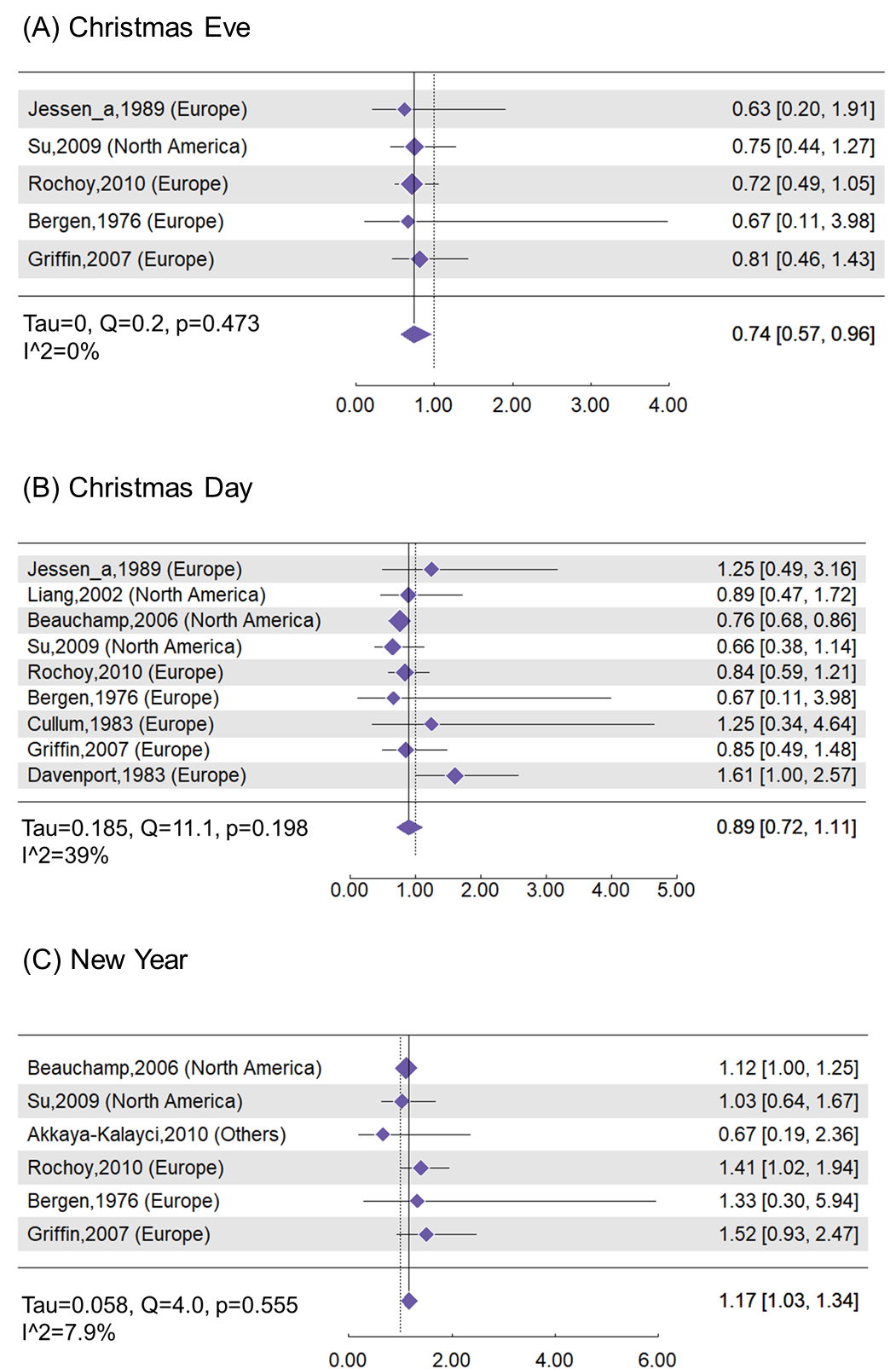


eFigure 15. Risk Ratio of Self-Harm and Suicidal Behaviors on Valentine’s Day Compared with Regular Day


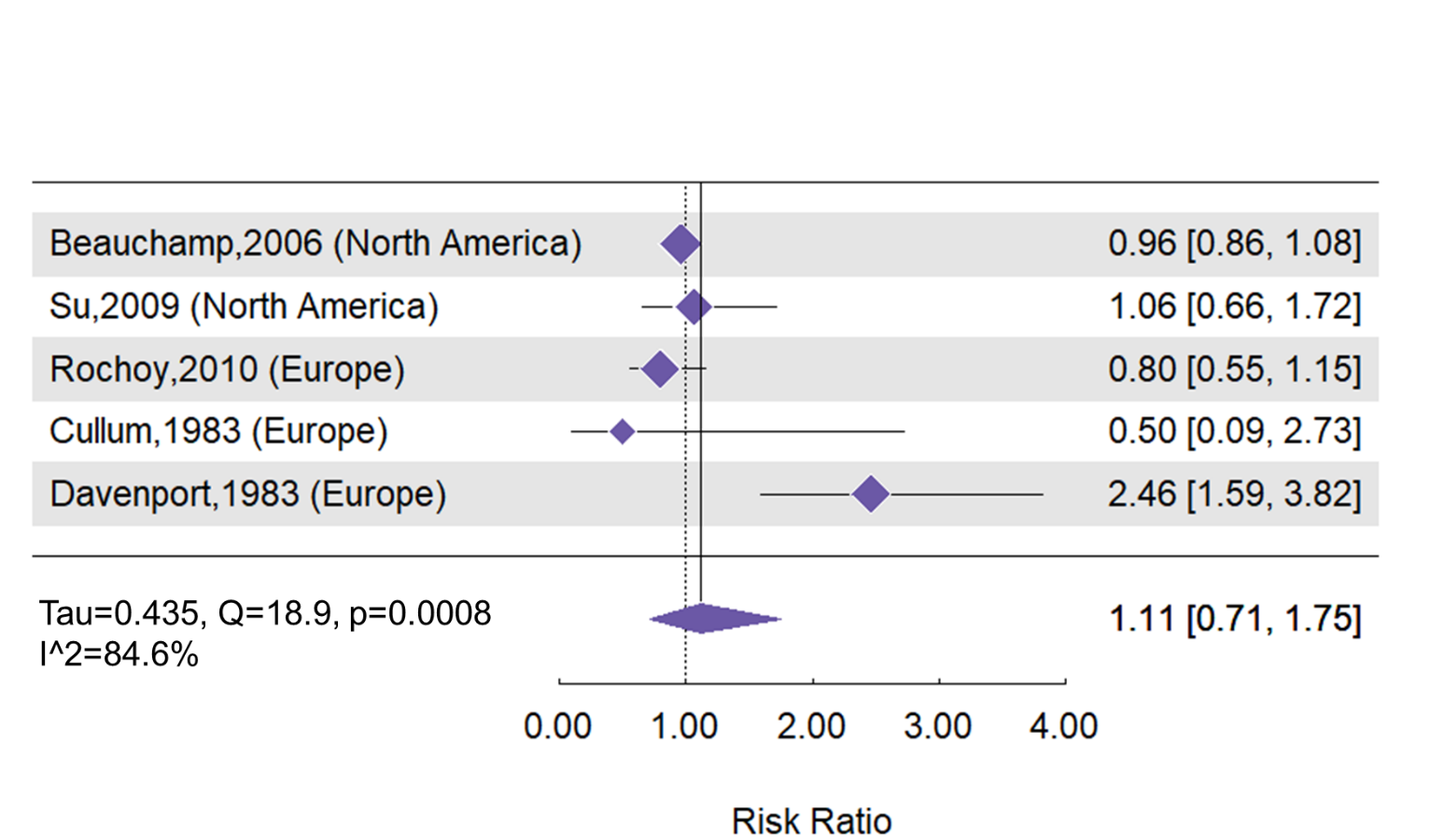


eFigure 16. Risk Difference of Self-Harm and Suicidal Behaviors on Christmas Eve, Christmas Day, and New Year’s Day Compared with Regular Day


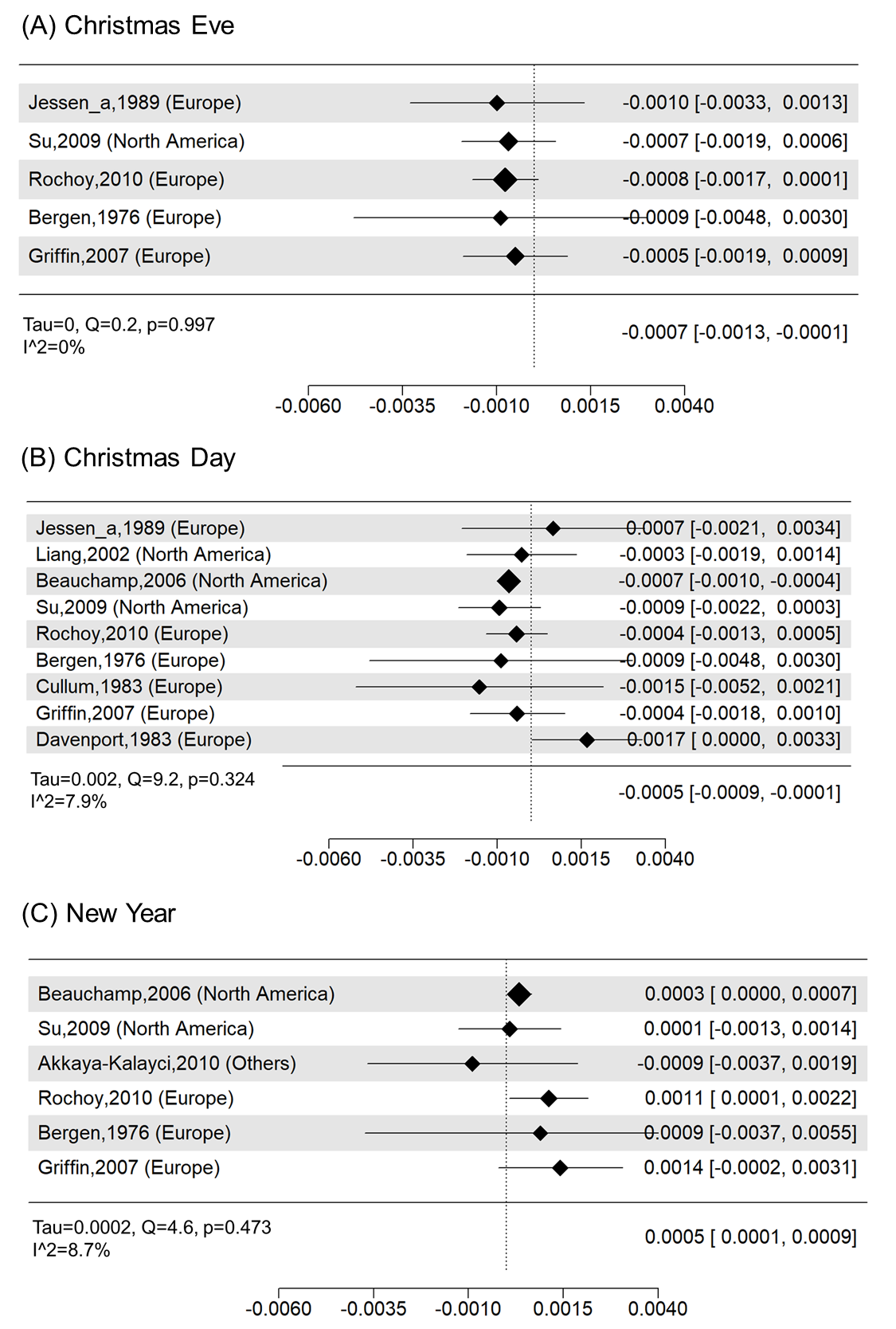


eFigure 17. Risk Difference of Self-Harm and Suicidal Behaviors on Valentine’s Day Compared with Regular Day


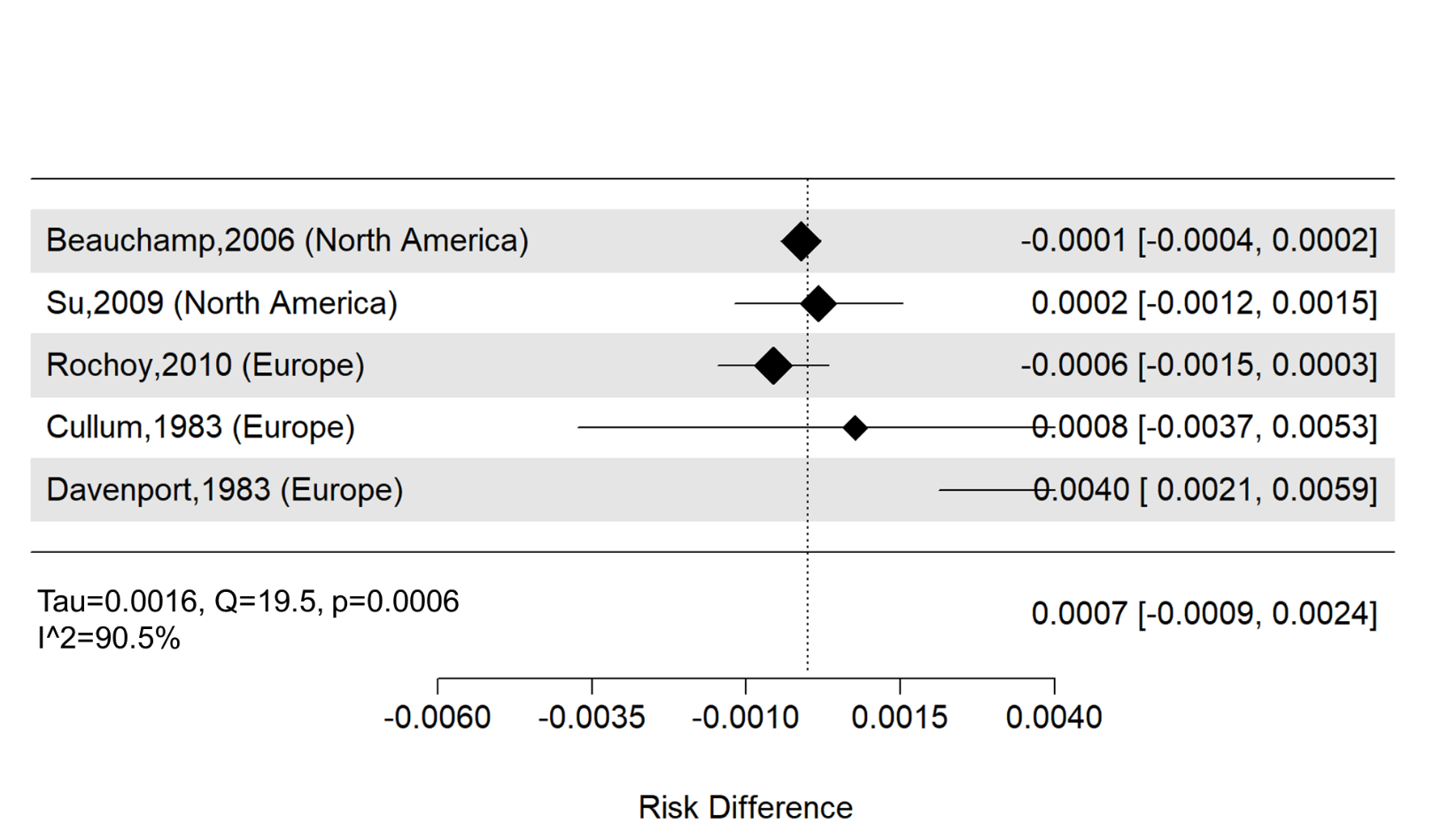


eFigure 18. Funnel Plot with Egger Test for the Proportion of Suicide on Christmas Even over a One-Year Period


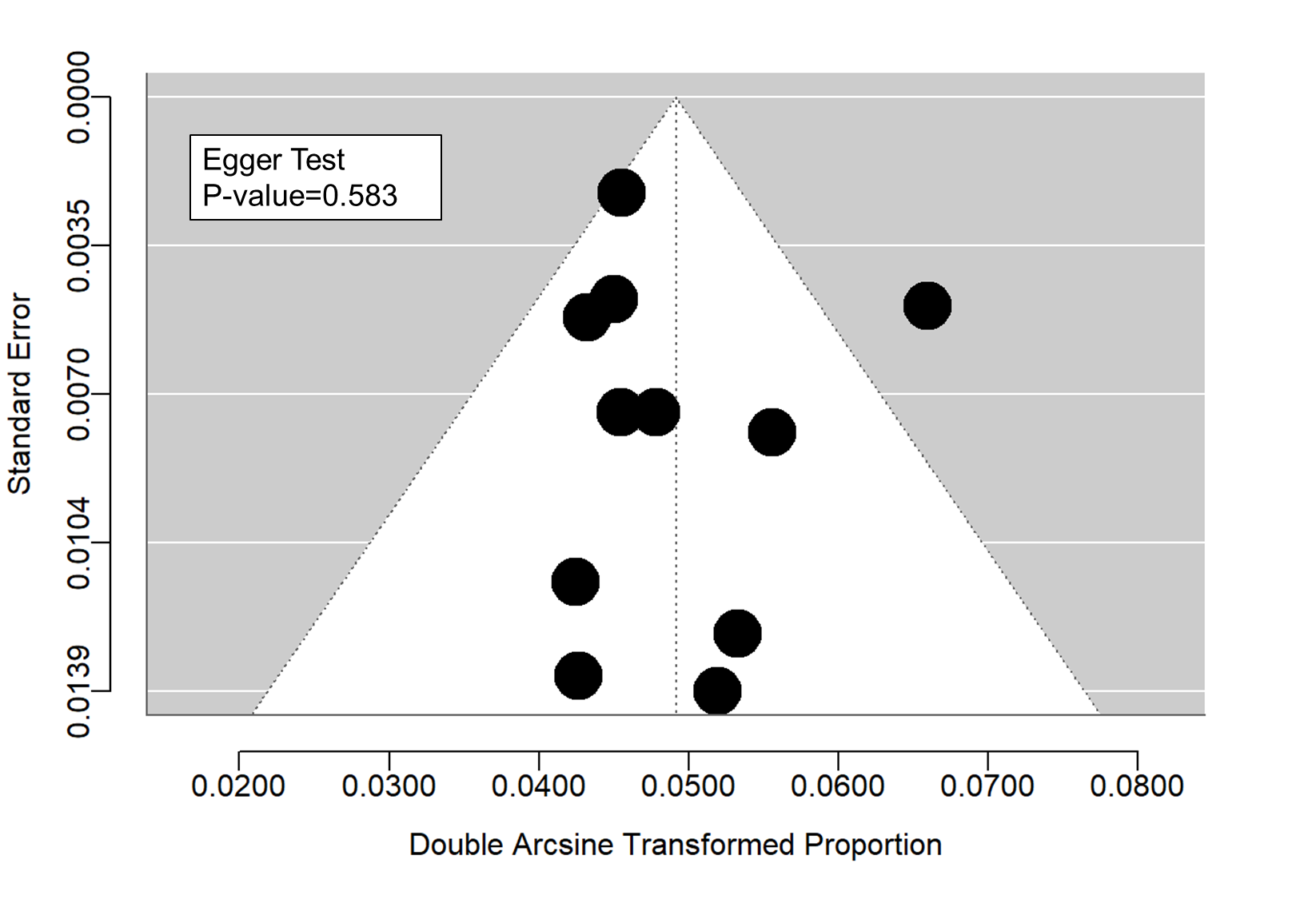


eFigure 19. Funnel Plot with Egger Test for the Proportion of Suicide on Christmas Day over a One-Year Period


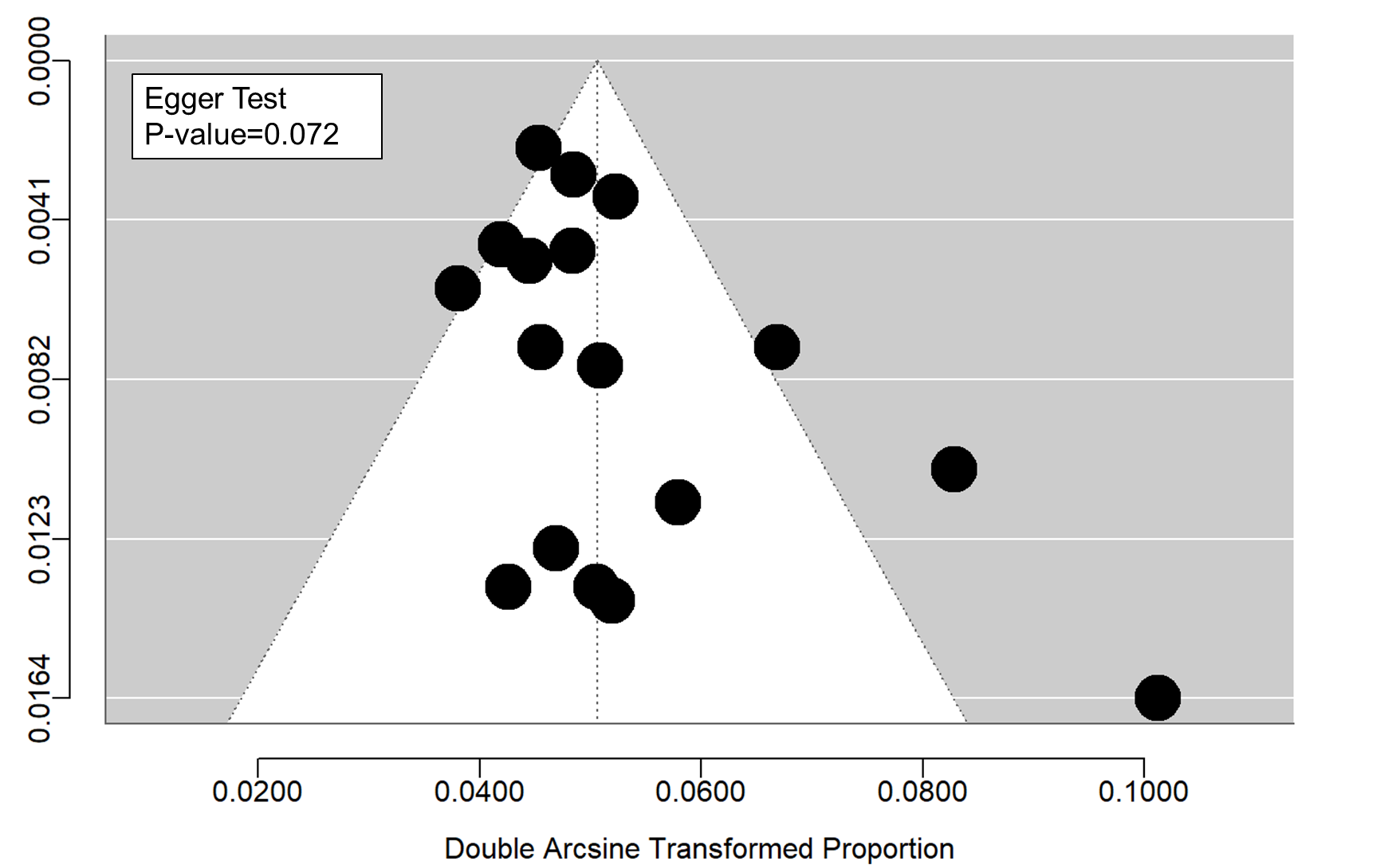


eFigure 20. Funnel Plot with Egger Test for the Proportion of Suicide on New Year’s Day over a One-Year Period


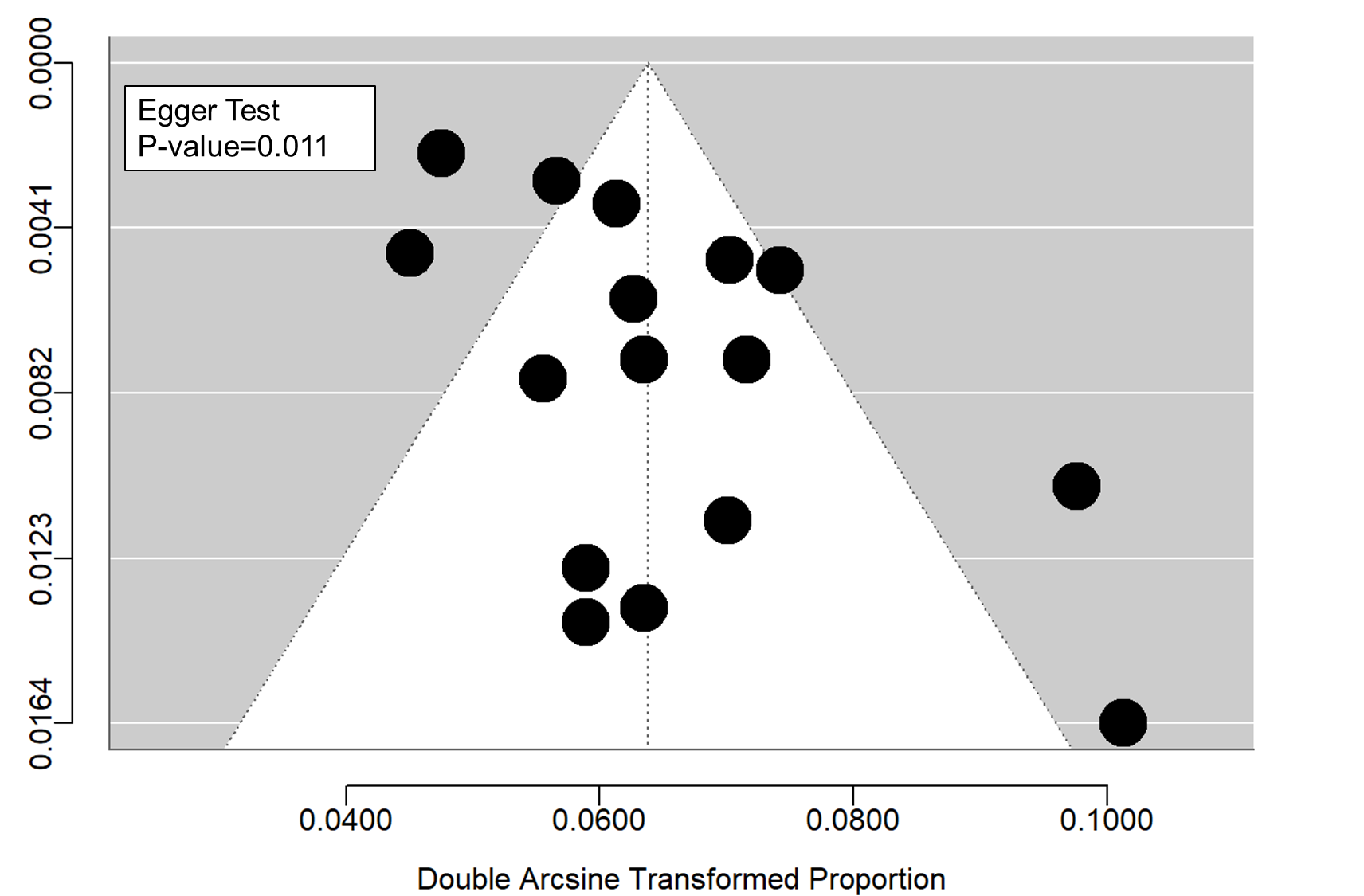


eFigure 21. Funnel Plot with Egger Test for the Proportion of Suicide on Valentine’s Day over a One-Year Period


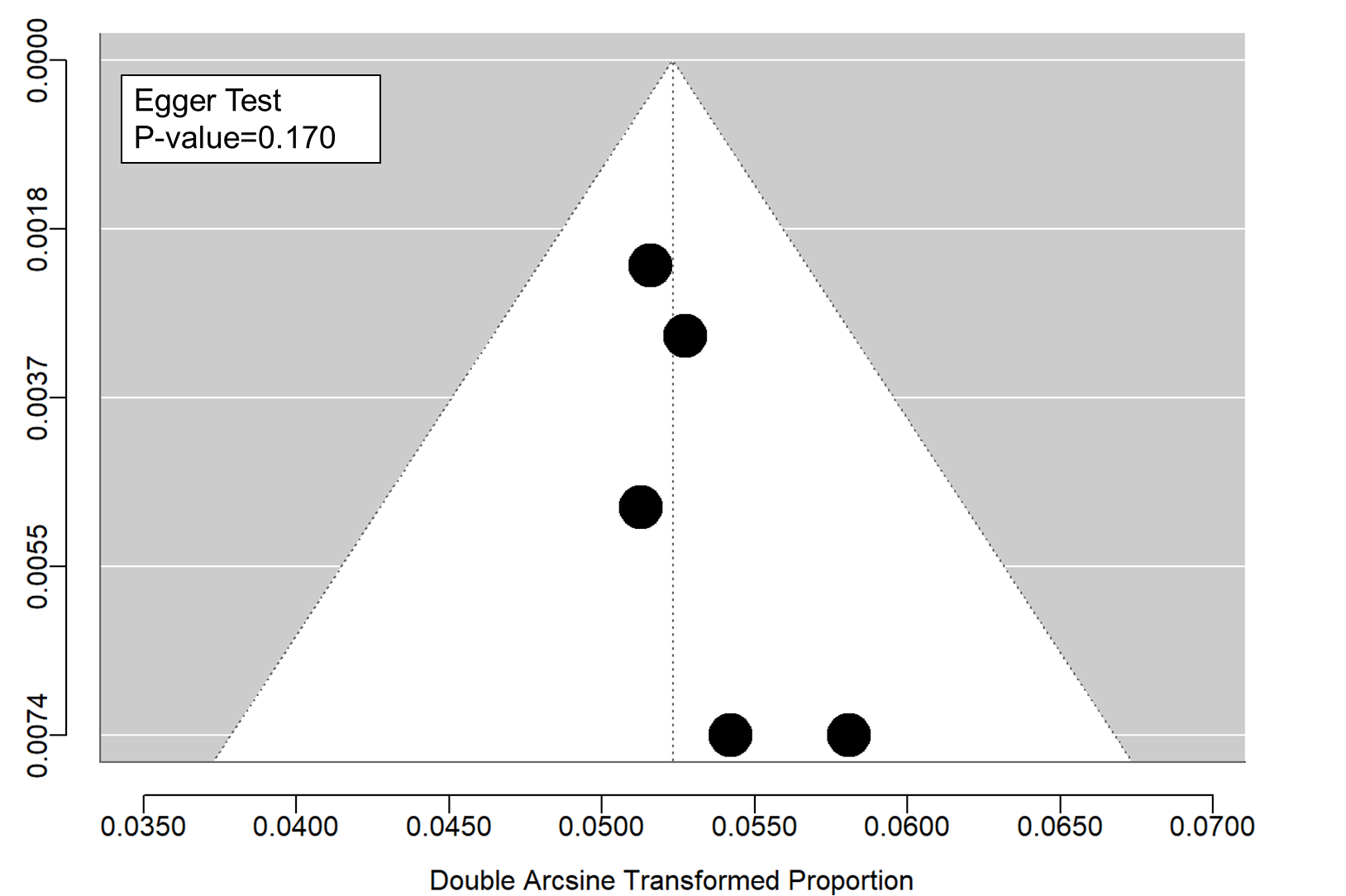


eFigure 22. Funnel Plot with Egger Test for the Risk Difference of Suicide on Christmas Eve vs Regular Day


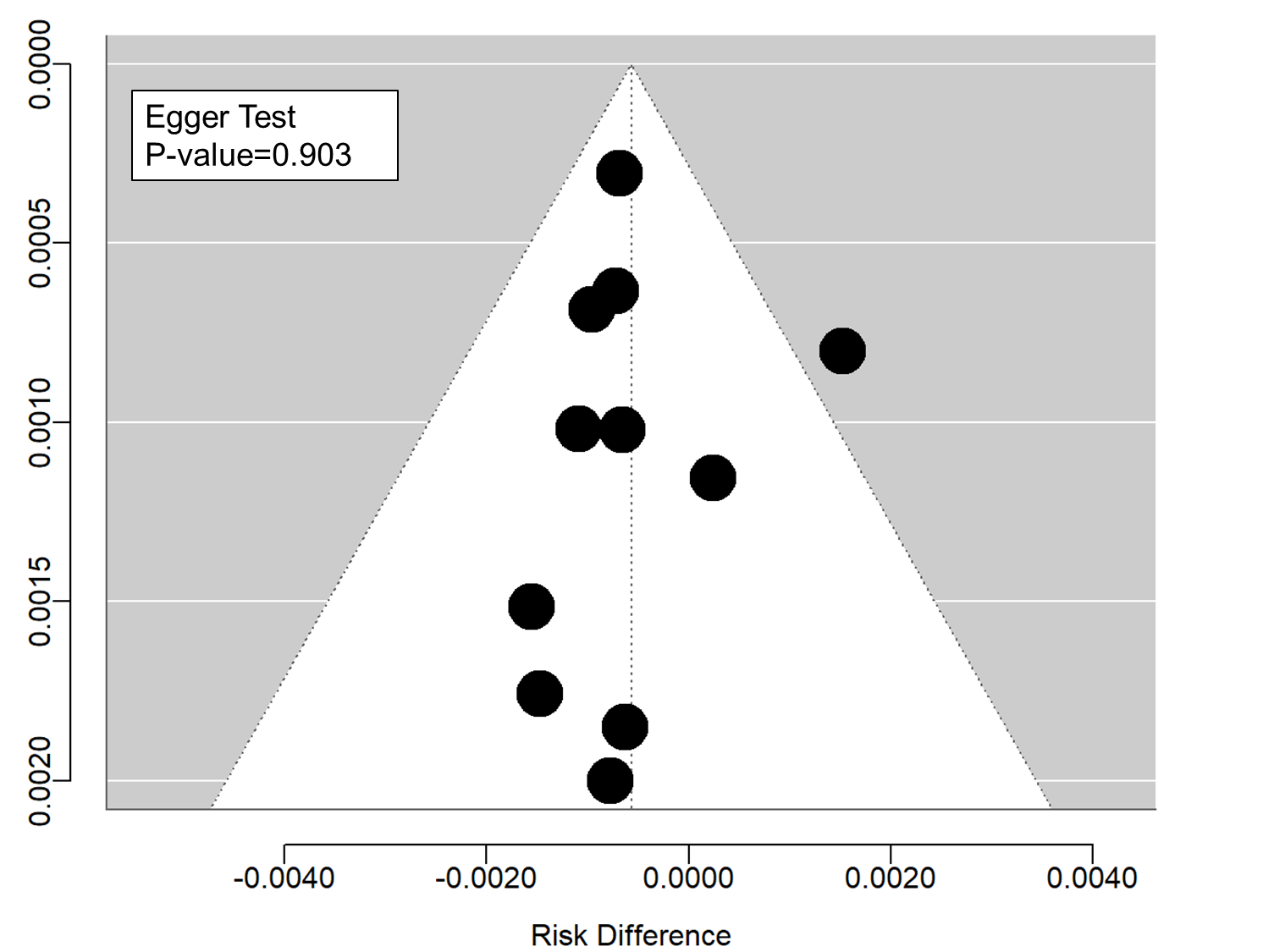


eFigure 23. Funnel Plot with Egger Test for the Risk Difference of Suicide on Christmas Day vs Regular Day


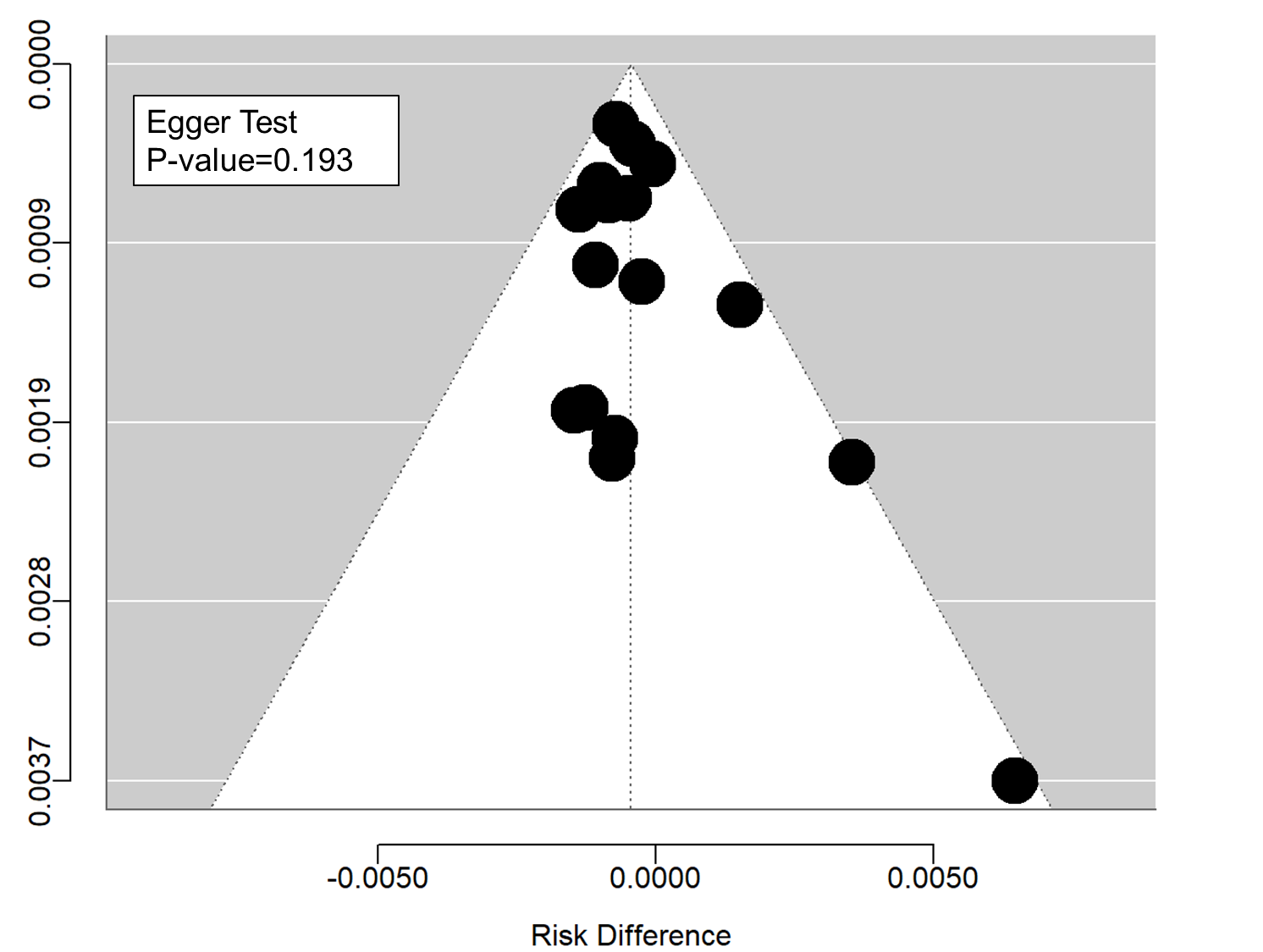


eFigure 24. Funnel Plot with Egger Test for the Risk Difference of Suicide on New Year’s Day vs Regular Day


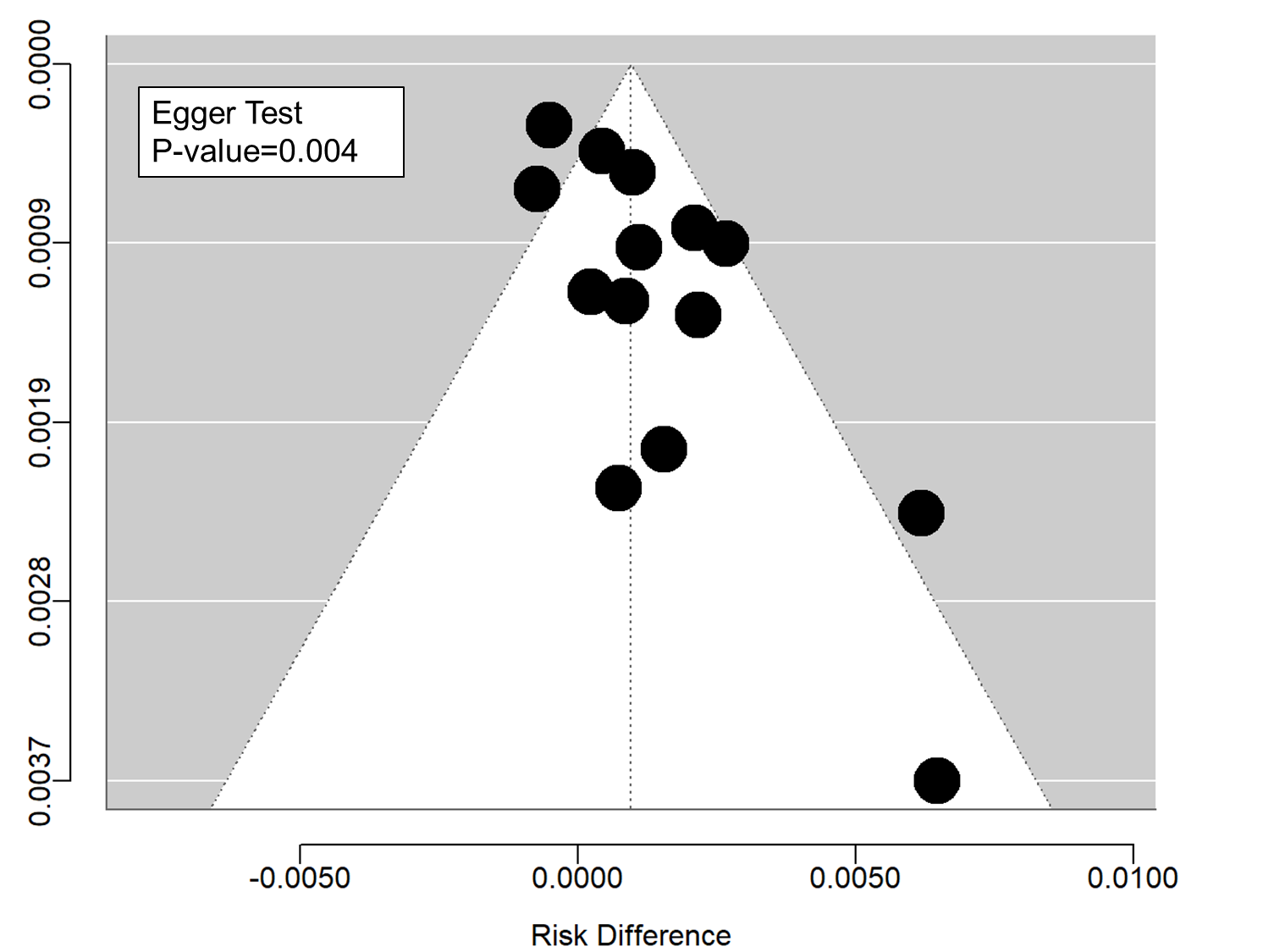


eFigure 25. Funnel Plot with Egger Test for the Risk Difference of Suicide on Valentine’s Day vs Regular Day


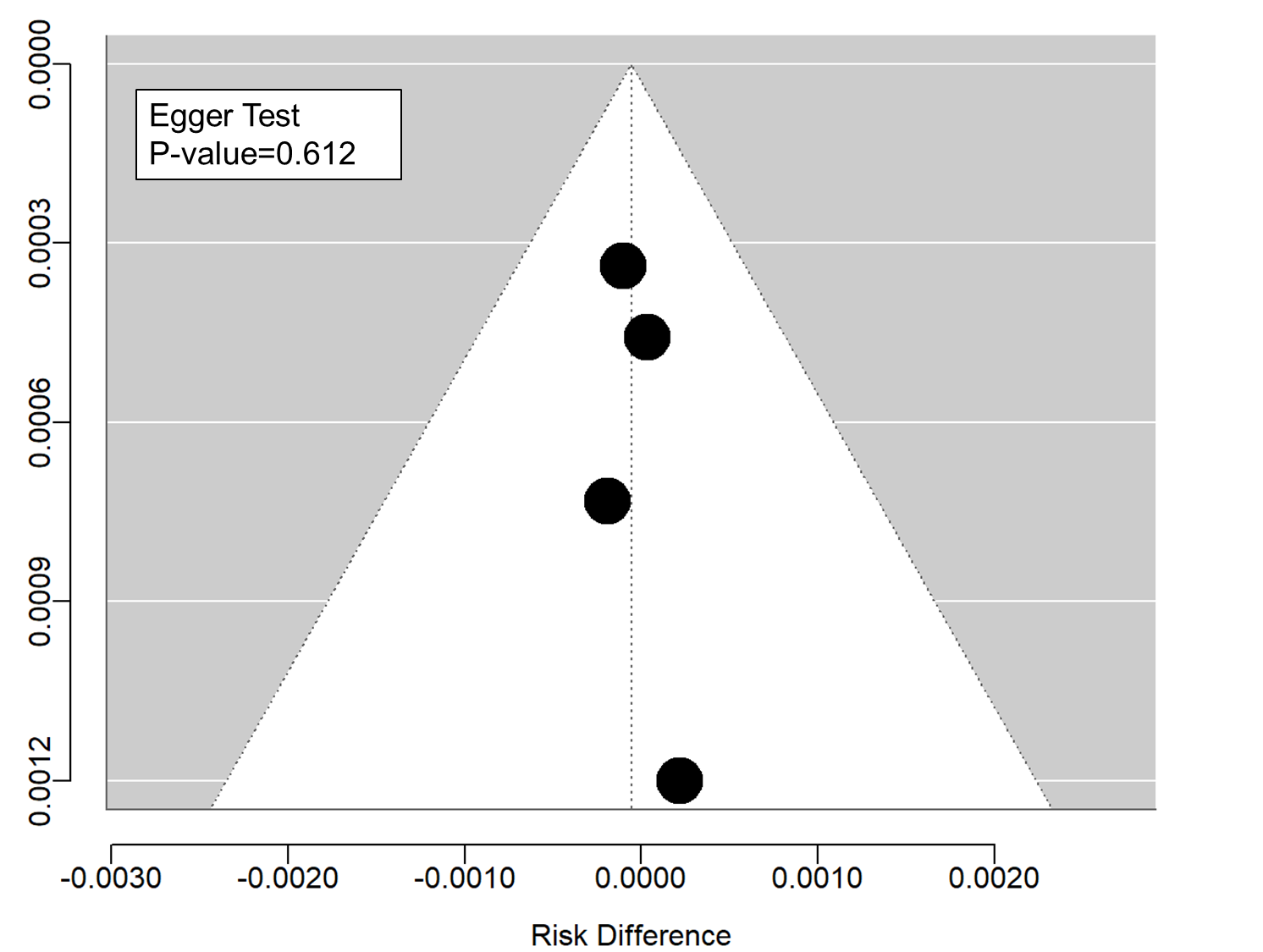


eFigure 26. Funnel Plot with Egger Test for the Risk Ratio of Suicide on Christmas Eve vs Regular Day


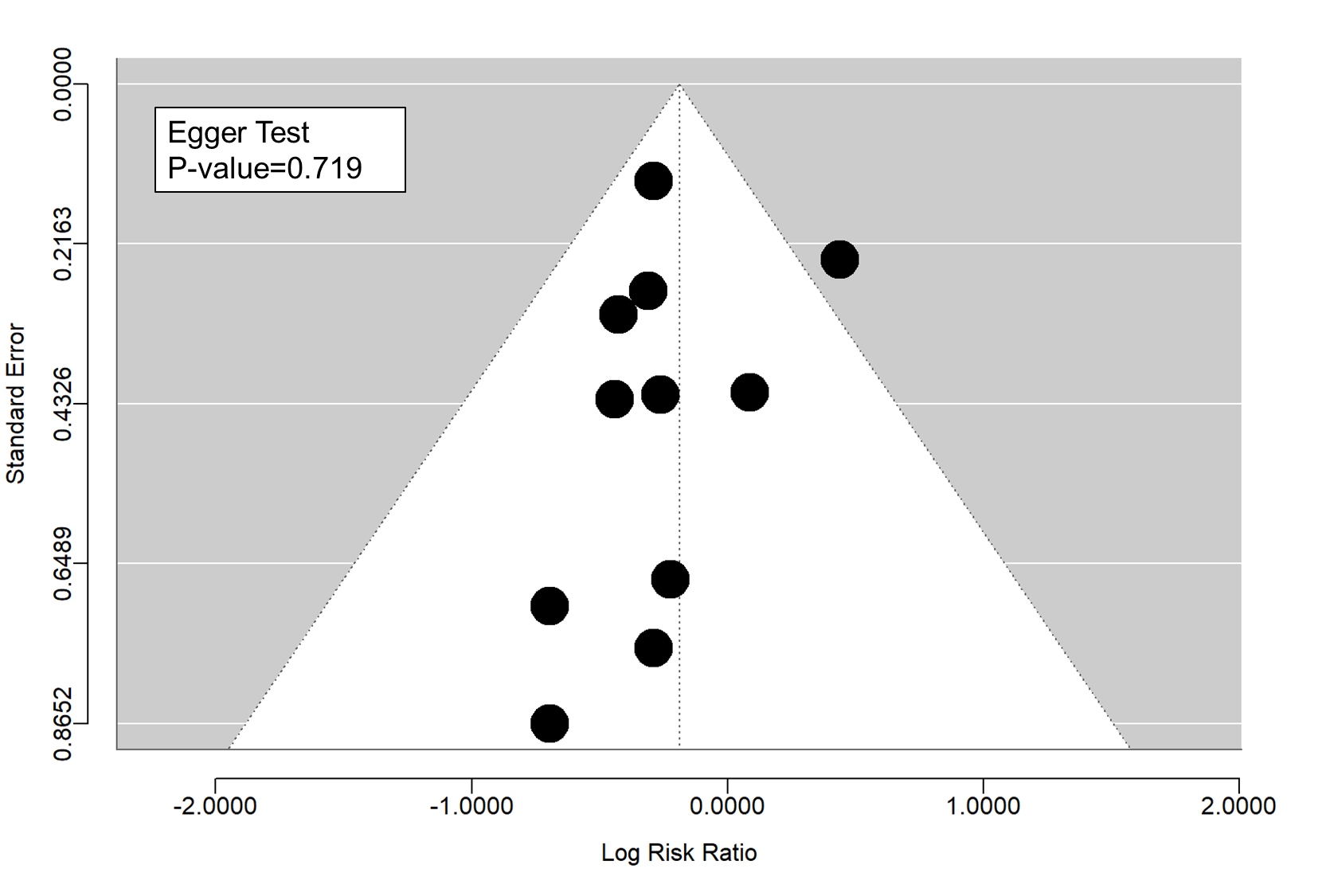


eFigure 27. Funnel Plot with Egger Test for the Risk Ratio of Suicide on Christmas Day vs Regular Day


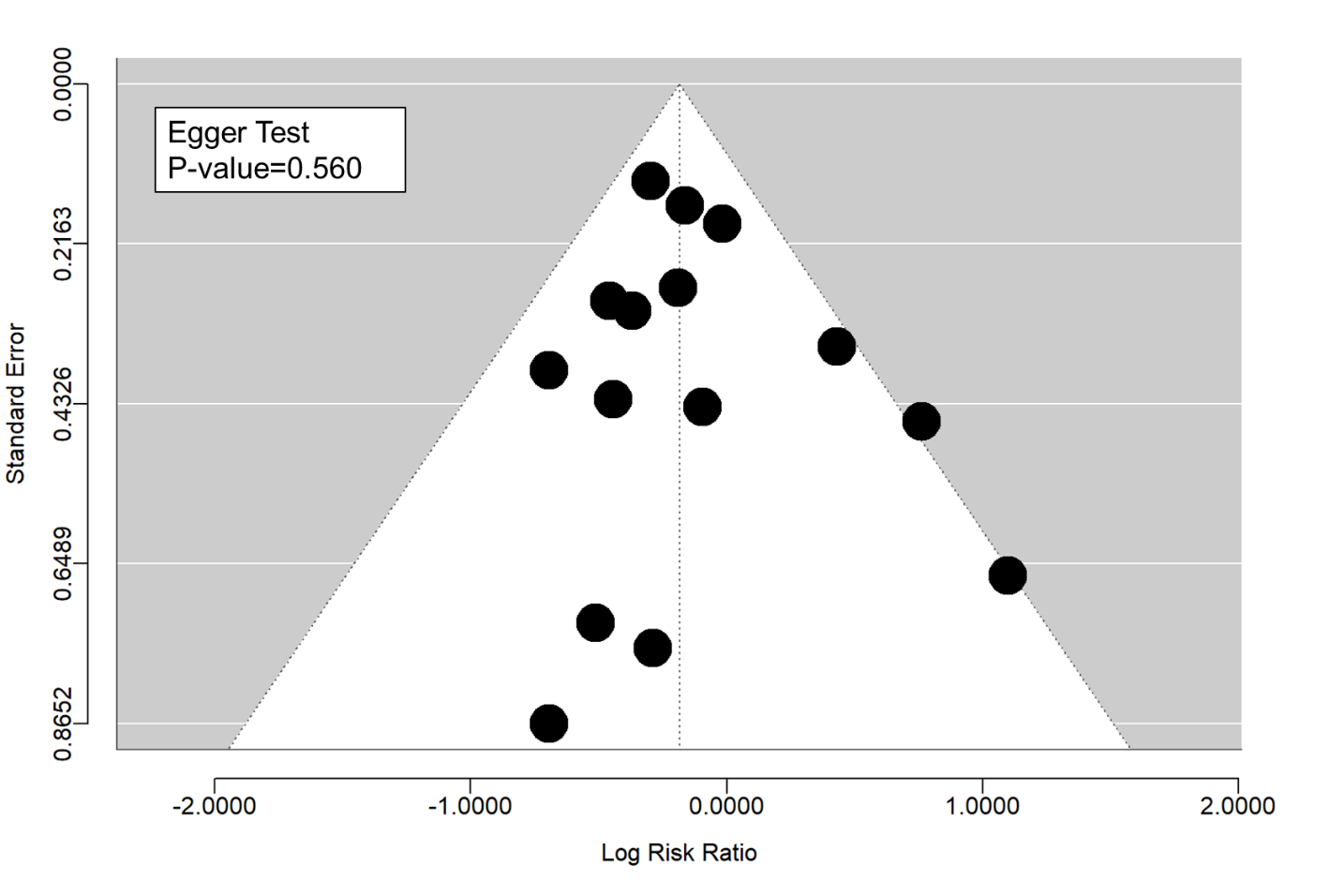


eFigure 28. Funnel Plot with Egger Test for the Risk Ratio of Suicide on New Year’s Day vs Regular Day


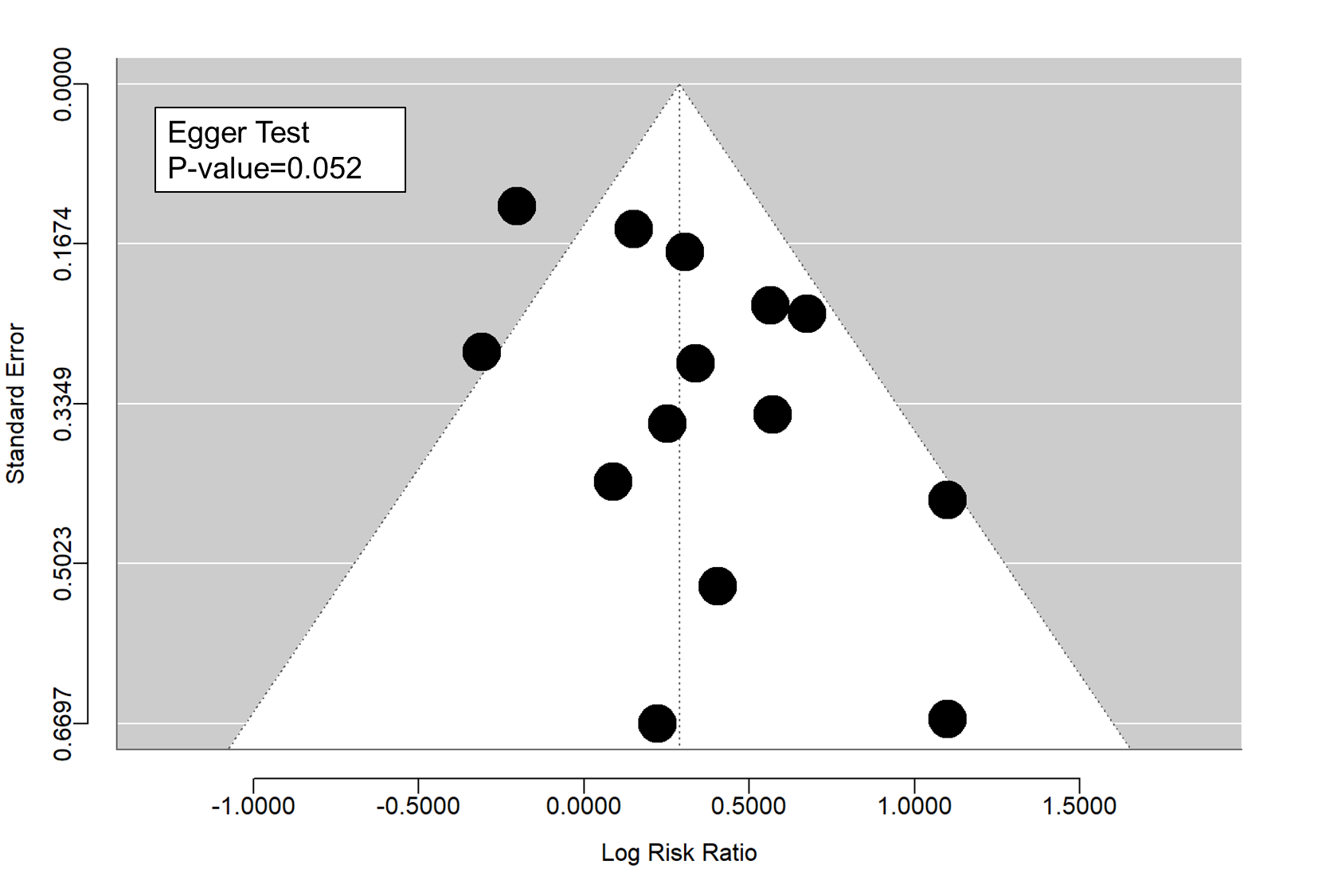


eFigure 29. Funnel Plot with Egger Test for the Risk Ratio of Suicide on Valentine’s Day vs Regular Day


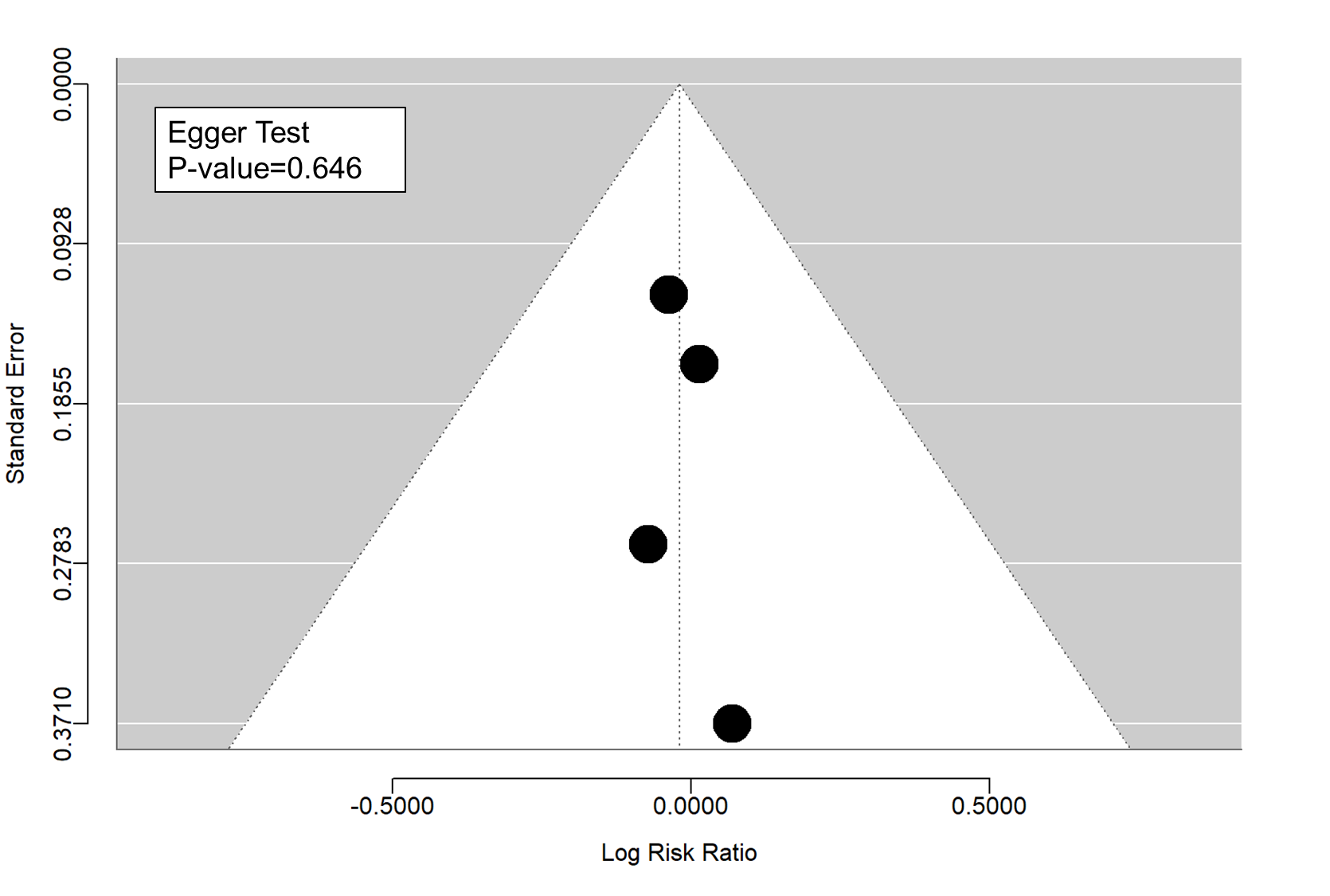


eFigure 30. Trim-and-Fill sensitivity analysis for the Christmas eve


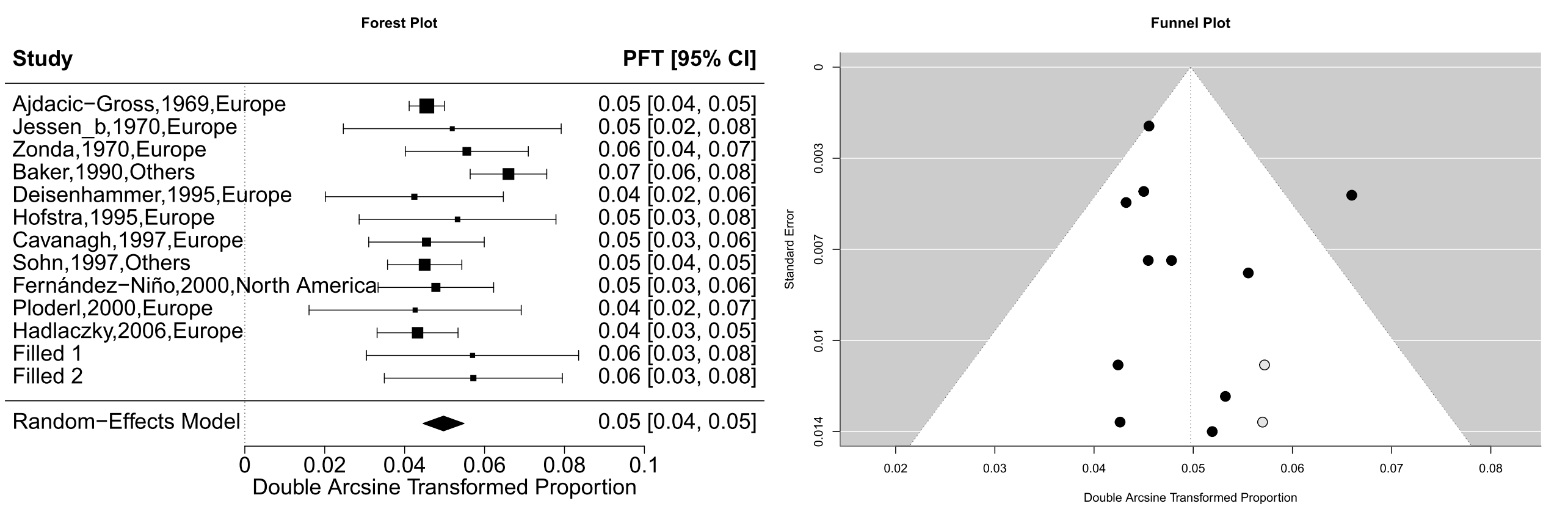


eFigure 31. Trim-and-Fill sensitivity analysis for the Christmas day


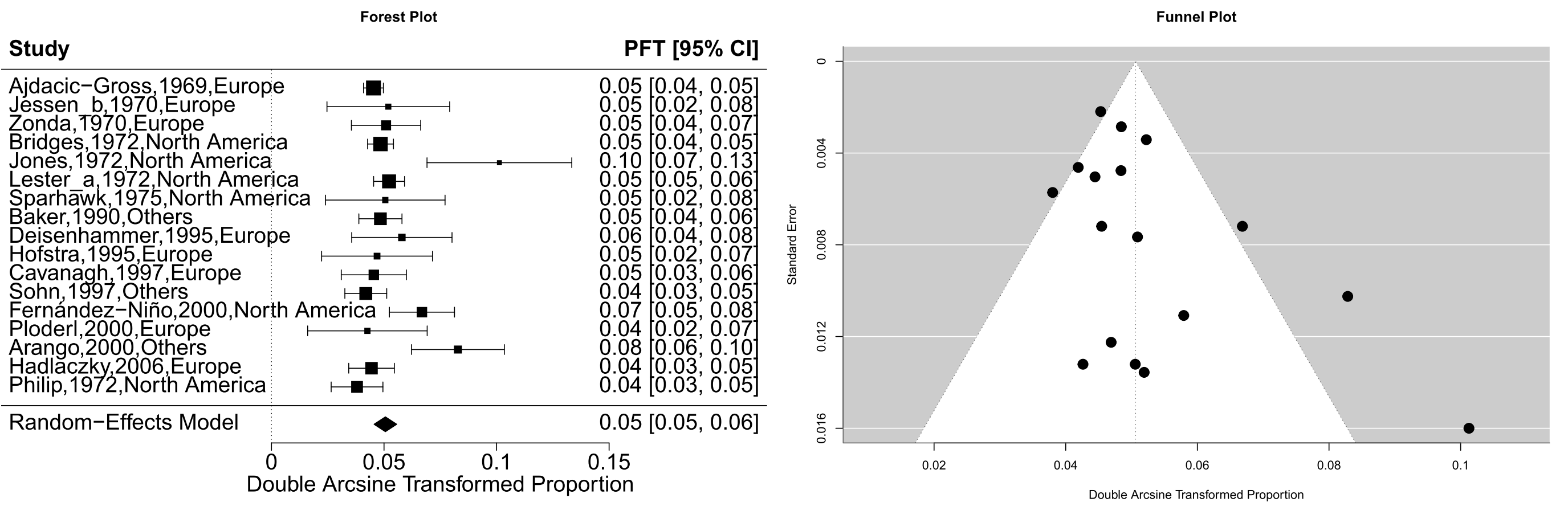


eFigure 32. Trim-and-Fill sensitivity analysis for the New Year


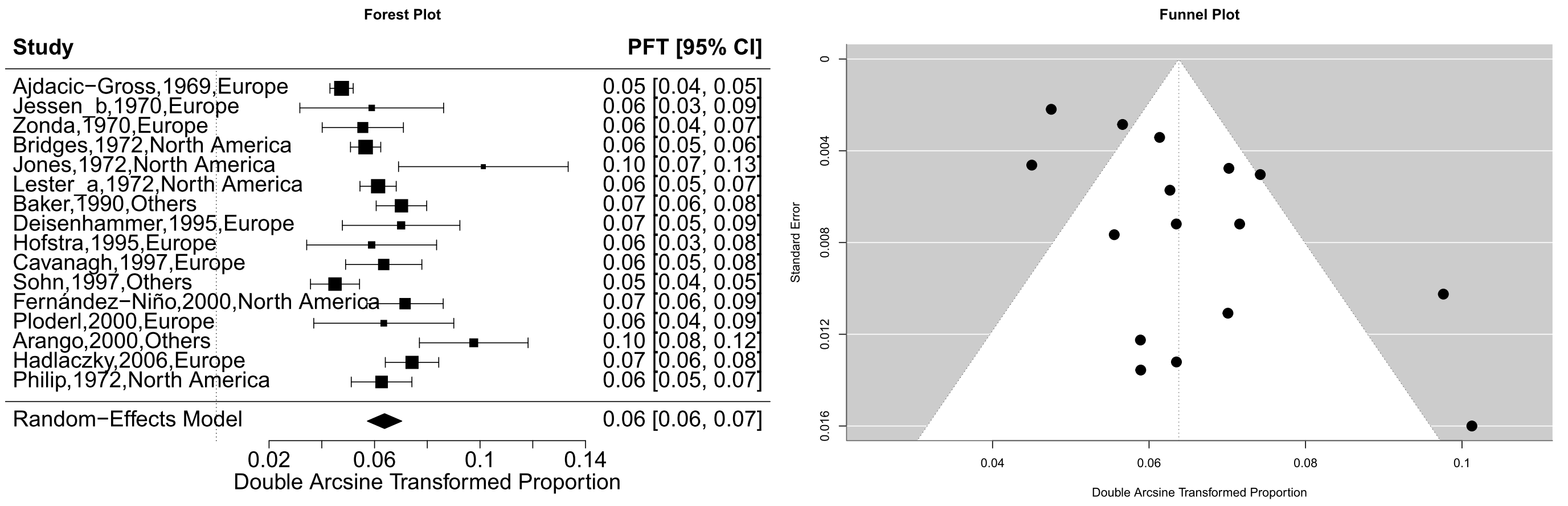


eFigure 33. Trim-and-Fill sensitivity analysis for the Valentine day


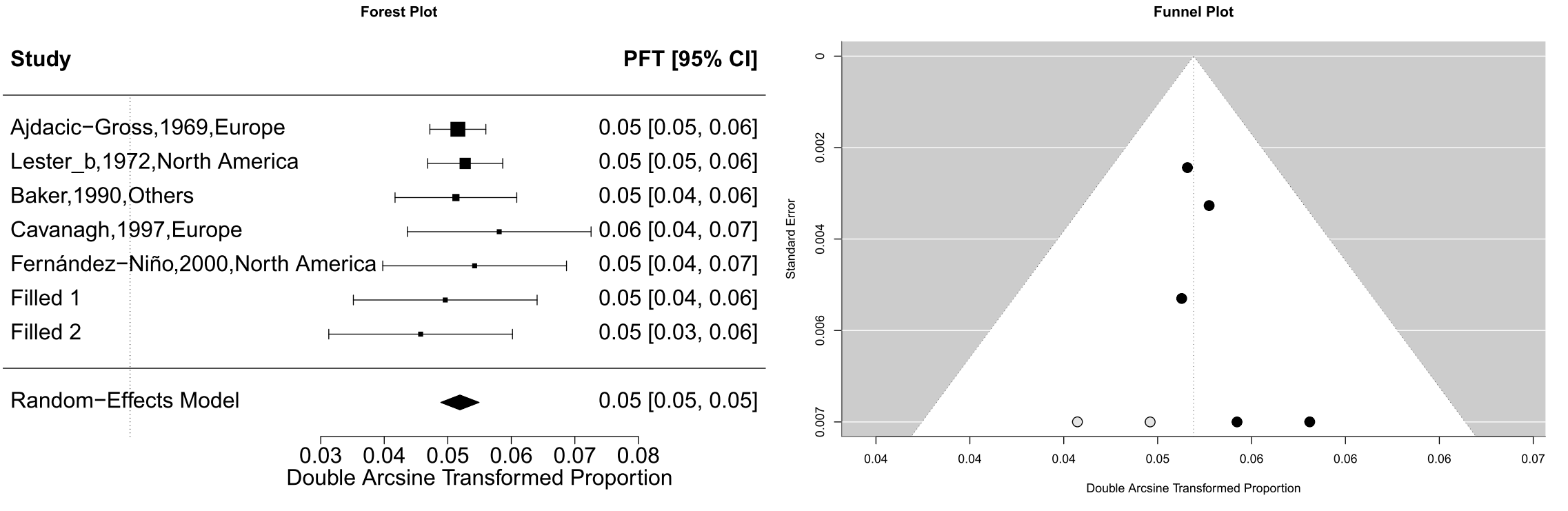


**eTable 1. The characteristics and demographics of the included studies.**

| **Study name** | **Country, Continent** | **Follow up duration** | **Data set (National / Local)** | **Total suicide individuals during follow-up period** | **Holiday** | | | | **Outcome of interest** | |
| --- | --- | --- | --- | --- | --- | --- | --- | --- | --- | --- |
|  |  |  |  |  | **Christmas’s eve** | **Christmas** | **New Year** | **Valentine’s Day** | **Completed Suicide** | **Suicide Attempt** |
| Ajdacic-Gross 2008 | Switzerland  Europe | 1969-2003 | Swiss mortality statistics  (National) | 49763 | V | V | V | V | V |  |
| Akkaya-Kalayci 2015 | Turkey  Asia | 2010 | Health Directorate of Istanbul, Ministry of Health (Local) | 2232 |  |  | V |  |  | V |
| Arango 2016 | Columbia  South America | 2000-2010 | Suicide registered with the National Bureau of statistics (National) | 24882 |  | V | V |  | V |  |
| Baker 2014 | Australia  Pacific | 1990-2009 | Queensland Suicide Register (Local) | 10465 | V | V | V | V | V |  |
| Beauchamp 2014 | United State  North America | 2006-2010 | American Association of Poison Control Centers (National) | 1065067 |  | V | V | V |  | V |
| Bergen 2007 | United Kingdom  Europe | 1976-2003 | Oxford Monitoring System for Attempted Suicide (Local) | 31369 | V | V | V |  |  | V |
| Bridges 2004 | United State  North America | 1972-1993 | National Center for Health Statistics (National) | NR |  | V | V |  | V |  |
| Cavanagh 2016 | United Kingdom  Europe | 1997-2012 | Suicide and Homicide by People with Mental Illness (National) | 73591 | V | V | V | V | V |  |
| Cullum 1993 | United Kingdom  Europe | 1983-1989 | Three hospital in London (Local) | NR |  | V |  | V |  | V |
| Davenport 1990 | United Kingdom  Europe | 1983-1988 | Central, South, and West Birmingham health districts (Local) | NR |  | V |  | V |  | V |
| Deisenhammer 2018 | Austria  Europe | 1995-2001  2004-2014 | Tyrol Suicide Register (Local) | 1980 | V | V | V |  | V |  |
| Fernández-Niño 2016 | Maxico  North America | 2000-2013 | Statistical and Epidemiological System of Deaths (National) | 64298 | V | V | V | V | V |  |
| Griffin 2017 | Ireland  Europe | 2007-2015 | National Self-Harm Registry Ireland (National) | 104371 | V | V | V |  |  | V |
| Hadlaczky 2018 | Sweden  Europe | 2006-2015 | Swedish cause of death registry (National) | 1047 | V | V | V |  | V |  |
| Hofstra 2018 | Netherland  Europe | 1995-2015 | Registered by Statistics Netherlands (National) | 33224 | V | V | V |  | V |  |
| Jessen 1999a | 11 Europe countries Europe | 1989-1996 | WHO/EURO Multicentre Study on Parasuicide (Local) | 24388 | V | V | V |  |  | V |
| Jessen 1999b | Danmark  Europe | 1970-1994 | Centre for Suicidological Research in Odense (National) | 32291 | V | V | V |  | V |  |
| Jones 1977 | United State  North America | 1972-1975 | Cuyahoga County Coroner’s Office (Local) | 928 |  | V | V |  | V |  |
| Lester 1985 | United State  North America | 1972-1978 | Vital Statistics of the United States (National) | NR |  | V | V |  | V |  |
| Lester 1990 | United State  North America | 1972-1987 | Vital Statistics of the United States (National) | NR |  |  |  | V | V |  |
| Liang 2011 | United State  North America | 2002-2007 | Illinois Poison Center (Local) | 43057 |  | V |  |  |  | V |
| Philip 1980 | United State  North America | 1972-1976 | Vital Statistics of the United States (National) | NR |  | V | V |  | V |  |
| Plöderl 2014 | Austria  Europe | 2000-2013 | Statistik Austria who administers the official death statistics (National) | NR | V | V | V |  | V |  |
| Rochoy 2024 | France  Europe | 2010-2019 | OSCOUR data base | 233242 | V | V | V | V |  | V |
| Sohn 2017 | Korea  Asia | 1997-2014 | National Prosecution Office of Korea (National) | 199971 | V | V | V |  | V |  |
| Sparhawk 1987 | United State  North America | 1975-1984 | Pennsylvania Department of Health (Local) | 13651 |  | V |  |  | V |  |
| Su 2020 | United State  North America | 2009-2012 | New York City Poison Control Center (Local) | 42578 | V | V | V | V |  | V |
| Zonda 2009 | Hungary  Europe | 1970-2002 | Hungarian Central Statistical Office (National) | 133699 | V | V | V |  | V |  |

NR, not reported; OSCOUR, Organisation for Coordianted Emergency Surveillance

Reference

1. Ajdacic-Gross V, Lauber C, Bopp M, et al. Reduction in the suicide rate during Advent--a time series analysis. *Psychiatry Res* 2008;157(1-3):139-46. doi: 10.1016/j.psychres.2006.07.014 [published Online First: 20071031]

2. Akkaya-Kalayci T, Popow C, Waldhor T, et al. Impact of religious feast days on youth suicide attempts in Istanbul, Turkey. *Neuropsychiatr* 2015;29(3):120-4. doi: 10.1007/s40211-015-0147-9 [published Online First: 20150616]

3. Barker E, O'Gorman J, De Leo D. Suicide around public holidays. *Australas Psychiatry* 2014;22(2):122-6. doi: 10.1177/1039856213519293 [published Online First: 20140204]

4. Beauchamp GA, Ho ML, Yin S. Variation in suicide occurrence by day and during major American holidays. *J Emerg Med* 2014;46(6):776-81. doi: 10.1016/j.jemermed.2013.09.023 [published Online First: 20140122]

5. Bergen H, Hawton K. Variation in deliberate self-harm around Christmas and New Year. *Soc Sci Med* 2007;65(5):855-67. doi: 10.1016/j.socscimed.2007.04.004 [published Online First: 20070522]

6. Bridges FS. Rates of homicide and suicide on major national holidays. *Psychol Rep* 2004;94(2):723-4. doi: 10.2466/pr0.94.2.723-724

7. Cardona Arango D, Medina-Perez OA, Cardona Duque DV. [Characterisation of Suicide in Colombia, 2000 2010]. *Rev Colomb Psiquiatr* 2016;45(3):170-7. doi: 10.1016/j.rcp.2015.10.002 [published Online First: 20151201]

8. Cavanagh B, Ibrahim S, Roscoe A, et al. The timing of general population and patient suicide in England, 1997-2012. *J Affect Disord* 2016;197:175-81. doi: 10.1016/j.jad.2016.02.055 [published Online First: 20160302]

9. Cullum SJ, Catalan J, Berelowitz K, et al. Deliberate self-harm and public holidays: is there a link? *Crisis* 1993;14(1):39-42.

10. Davenport SM, Birtle J. Association between parasuicide and Saint Valentine's Day. *BMJ* 1990;300(6727):783-4. doi: 10.1136/bmj.300.6727.783

11. Deisenhammer EA, Stiglbauer C, Kemmler G. [Time-related aspects of suicides - suicide frequency related to birthday, major holidays, day of the week, season, month of birth and zodiac signs]. *Neuropsychiatr* 2018;32(2):93-100. doi: 10.1007/s40211-018-0260-7 [published Online First: 20180307]

12. Fernandez-Nino JA, Astudillo-Garcia CI, Bojorquez-Chapela I, et al. The Mexican Cycle of Suicide: A National Analysis of Seasonality, 2000-2013. *PLoS One* 2016;11(1):e0146495. doi: 10.1371/journal.pone.0146495 [published Online First: 20160111]

13. Griffin E, Dillon CB, O'Regan G, et al. The paradox of public holidays: Hospital-treated self-harm and associated factors. *J Affect Disord* 2017;218:30-34. doi: 10.1016/j.jad.2017.04.058 [published Online First: 20170425]

14. Hadlaczky G, Hokby S. Increased suicides during new year, but not during Christmas in Sweden: analysis of cause of death data 2006-2015. *Nord J Psychiatry* 2018;72(1):72-74. doi: 10.1080/08039488.2017.1378716 [published Online First: 20170922]

15. Hofstra E, Elfeddali I, Bakker M, et al. Springtime Peaks and Christmas Troughs: A National Longitudinal Population-Based Study into Suicide Incidence Time Trends in the Netherlands. *Front Psychiatry* 2018;9:45. doi: 10.3389/fpsyt.2018.00045 [published Online First: 20180226]

16. Jessen G, Jensen BF, Arensman E, et al. Attempted suicide and major public holidays in Europe: findings from the WHO/EURO Multicentre Study on Parasuicide. *Acta Psychiatr Scand* 1999;99(6):412-8. doi: 10.1111/j.1600-0447.1999.tb00986.x

17. Jessen G, Jensen BF. Postponed suicide death? Suicides around birthdays and major public holidays. *Suicide Life Threat Behav* 1999;29(3):272-83.

18. Jones PK, Jones SL. Lunar association with suicide. *Suicide Life Threat Behav* 1977;7(1):31-9.

19. Lester D. Suicide at Christmas. *Am J Psychiatry* 1985;142(6):782. doi: 10.1176/ajp.142.6.782a

20. Lester D. Suicide and homicide on St. Valentine's Day. *Percept Mot Skills* 1990;71(3 Pt 1):994. doi: 10.2466/pms.1990.71.3.994

21. Liang S, Friedman LS. Analysis of suspected suicides using poison center data. *Arch Suicide Res* 2011;15(3):185-94. doi: 10.1080/13811118.2011.589674

22. Phillips DP, Wills JS. A drop in suicides around major national holidays. *Suicide Life Threat Behav* 1987;17(1):1-12. doi: 10.1111/j.1943-278x.1987.tb00057.x

23. Ploderl M, Fartacek C, Kunrath S, et al. Nothing like Christmas--suicides during Christmas and other holidays in Austria. *Eur J Public Health* 2015;25(3):410-3. doi: 10.1093/eurpub/cku169 [published Online First: 20140922]

24. Rochoy M, Pontais I, Caserio-Schonemann C, et al. Pattern of encounters to emergency departments for suicidal attempts in France: Identification of high-risk days, months and holiday periods. *Encephale* 2024 doi: 10.1016/j.encep.2023.11.018 [published Online First: 20240204]

25. Sohn K. Suicides around Major Public Holidays in South Korea. *Suicide Life Threat Behav* 2017;47(2):217-27. doi: 10.1111/sltb.12281 [published Online First: 20160723]

26. Sparhawk TG. Traditional holidays and suicide. *Psychol Rep* 1987;60(1):245-6. doi: 10.2466/pr0.1987.60.1.245

27. Su MK, Chan PY, Hoffman RS. The seasonality of suicide attempts: a single poison control center perspective. *Clin Toxicol (Phila)* 2020;58(11):1034-41. doi: 10.1080/15563650.2020.1733591 [published Online First: 20200306]

28. Zonda T, Bozsonyi K, Veres E, et al. The impact of holidays on suicide in Hungary. *Omega (Westport)* 2008;58(2):153-62. doi: 10.2190/om.58.2.e

eTable 2. Risk of bias of included reports from studies as assessed with the Newcastle-Ottawa Scale

| **Study name** | Selection (4) | Comparability (2) | Outcome (3) | Total (9) | Comments |
| --- | --- | --- | --- | --- | --- |
| Ajdacic-Gross 2008 | 4 | 1 | 3 | 8 |  |
| Akkaya-Kalayci 2015 | 3 | 2 | 2 | 7 | Hospital record (S-1), Non-holidays as reference (C+1), Short follow-up period (O-1) |
| Arango 2016 | 4 | 1 | 3 | 8 |  |
| Baker 2014 | 4 | 1 | 3 | 8 |  |
| Beauchamp 2014 | 4 | 0 | 2 | 6 | Use selected dates as reference (C-1), Short follow-up period (O-1) |
| Bergen 2007 | 3 | 0 | 3 | 6 | Hospital record (S-1), Use average of selected dates as reference (C-1) |
| Bridges 2004 | 4 | 1 | 3 | 8 |  |
| Cavanagh 2016 | 4 | 1 | 3 | 8 |  |
| Cullum 1993 | 3 | 0 | 3 | 6 | Hospital record (S-1), Use selected dates as reference (C-1) |
| Davenport 1990 | 3 | 0 | 3 | 6 | Hospital record (S-1), Use selected dates as reference (C-1) |
| Deisenhammer 2018 | 4 | 2 | 3 | 9 | Non-holidays as reference (C+1) |
| Fernández-Niño 2016 | 4 | 0 | 3 | 7 | Use selected dates as reference (C-1) |
| Griffin 2017 | 4 | 2 | 3 | 9 | Non-holidays as reference (C+1) |
| Hadlaczky 2018 | 4 | 0 | 2 | 6 | Use average of selected dates as reference (C-1), Short follow-up period (O-1) |
| Hofstra 2018 | 4 | 0 | 3 | 7 | Use average of selected dates as reference (C-1) |
| Jessen 1999a | 3 | 1 | 3 | 7 | Hospital record (S-1) |
| Jessen 1999b | 4 | 1 | 3 | 8 |  |
| Jones 1977 | 4 | 1 | 2 | 7 | Short follow-up period (O-1) |
| Lester 1985 | 4 | 0 | 3 | 7 | Use selected dates as reference (C-1) |
| Lester 1990 | 4 | 0 | 3 | 7 | Use selected dates as reference (C-1) |
| Liang 2011 | 4 | 1 | 3 | 8 |  |
| Philip 1980 | 4 | 1 | 2 | 7 | Short follow-up period (O-1) |
| Plöderl 2014 | 4 | 1 | 3 | 8 |  |
| Rochoy 2024 | 4 | 1 | 3 | 8 |  |
| Sohn 2017 | 4 | 1 | 3 | 8 |  |
| Sparhawk 1987 | 4 | 1 | 3 | 8 |  |
| Su 2020 | 4 | 0 | 2 | 6 | Use selected dates as reference (C-1), Short follow-up period (O-1) |
| Zonda 2009 | 4 | 1 | 3 | 8 |  |

eTable 3. Meta-regression of Proportion of Annual Suicides Occurring on Christmas Eve, Christmas Day, and NewYear's Day, and Valentine’s Day Compared with Regular Days

| Group | Estimates | P value |
| --- | --- | --- |
| Intercept | 0.0531 (0.0508, 0.0553) | <0.001 |
| Christmas Eve vs regular day | -0.0049 (-0.0082, -0.0016) | 0.0039 |
| Christmas Day vs regular day | -0.0051 (-0.0089, -0.0012) | 0.0095 |
| New Year’s Day vs regular day | 0.0044 (0.0011, 0.0077) | 0.0097 |
| Valentine’s Day vs regular day | -0.0008 (-0.0047, 0.0031) | 0.6947 |

^a^ The proportion of annual suicide occurring on regular days is the average daily number of suicides over 365 days

eTable 4. Meta-regression of Proportion of Annual Suicides Occurring on Holiday

| **Outcome** | **Covariate** | **Coefficient (95% CI)** | **QM** | **P-value** | **I^2** |
| --- | --- | --- | --- | --- | --- |
| Christmas Eve | **Design** | -0.0163 (-0.0257 to -0.0069) | 11.6 | **<0.0001*** | **0%** |
| (k=11) | Continent | NA | 1.8 | 0.389 | 45.1% |
|  | **Country** | NA | 17.7 | **0.039*** | **0%** |
|  | Duration | 0.0001 (-0.0006 to 0.0008) | 0.1 | 0.735 | 46.7% |
|  | Year^a^ | -0.0001 (-0.0006 to 0.0003) | 0.3 | 0.590 | 50.4% |
| Christmas Day | Design | -0.0082 (-0.0221 to 0.0057) | 1.3 | 0.250 | 67.9% |
| (k=17) | Continent | NA | 1.9 | 0.389 | 45.1% |
|  | Country | NA | 5.8 | 0.889 | 84.1% |
|  | Duration | -0.0003 (-0.0009 to 0.0003) | 0.8 | 0.347 | 65.9% |
|  | Year | 0.0001 (-0.0003 to 0.0005) | 0.2 | 0.677 | 68.1% |
| New Year’s Day | Design | -0.0156 (-0.0330 to 00018) | 2.5 | 0.111 | 78.9% |
| (k=16) | Continent | NA | 0.7 | 0.733 | 80.7% |
|  | **Country** | NA | 47.1 | **<0.001*** | **18.0%** |
|  | **Duration** | -0.0008 (-0.0014 to -0.0002) | 6.8 | **0.009*** | **68.4%** |
|  | Year | 0.0004 (-0.0001 to 0.0008) | 2.5 | 0.112 | 75.8% |

Abbreviation: CI=confidence interval; NA=not applicable; RR=risk ratio; QM=Q value of test of moderators

^a^ Year represents the starting year of each study.

^b^ I^2: Estimates how much of the residual heterogeneity is attributable to unaccounted variability in the meta-regression model. It is expressed as a percentage.

eTable 5. Meta-regression of Risk Ratio (Compared with Regular Days)

| **Outcome** | **Covariate** | **Coefficient (95% CI)** | **QM** | **P-value** | **I^2** |
| --- | --- | --- | --- | --- | --- |
| Christmas Eve | **Design** | -0.618 (-1.0998 to -0.1363) | 6.3 | **0.012*** | **0%** |
| (k=11) | Continent | NA | 3.3 | 0.193 | 8.3% |
|  | Country | NA | 10.0 | 0.351 | 0% |
|  | Duration | 0.0047 (-0.0254 to 0.0348) | 0.1 | 0.760 | 29.7% |
|  | Year | -0.0040 (-0.0239 to 0.0160) | 0.2 | 0.696 | 33.3% |
| Christmas Day | Design | -0.1700 (-0.6654 to 0.3254) | 0.5 | 0.501 | 0% |
| (k=17) | Continent | NA | 2.6 | 0.274 | 0% |
|  | Country | NA | 10.5 | 0.484 | 0% |
|  | Duration | -0.0079 (-0.0209 to 0.0051) | 1.4 | 0.232 | 0% |
|  | Year | 0.0021 (-0.0089 to 0.0131) | 0.1 | 0.708 | 0% |
| New Year’s Day | Design | -0.3808 (-0.9601 to 0.1985) | 1.7 | 0.198 | 49.0% |
| (K=16) | Continent | NA | 0.5 | 0.781 | 54.3% |
|  | **Country** | NA | 25.4 | **0.003*** | **0%** |
|  | **Duration** | -0.0237 (-0.0382 to -0.0092) | 10.3 | **0.001*** | **13.1%** |
|  | Year | 0.0118 (-0.0017 to 0.0254) | 2.9 | 0.087 | 39.5% |

Abbreviation: CI=confidence interval; NA=not applicable; RR=risk ratio; QM=Q value of test of moderators

^a^ Year represents the starting year of each study.

^b^ I^2: Estimates how much of the residual heterogeneity is attributable to unaccounted variability in the meta-regression model. It is expressed as a percentage.

eTable 6. Meta-regression of Risk Difference (Compared with Regular Days)

| **Outcome** | **Covariate** | **Coefficient (95% CI)** | **QM** | **P-value** | **I^2** |
| --- | --- | --- | --- | --- | --- |
| Christmas Eve | **Design** | -0.0016 (-0.0031 to -0.0001) | 4.3 | **0.039*** | **0%** |
| (k=10) | Continent | NA | 2.4 | 0.296 | 0.3% |
|  | Country | NA | 8.4 | 0.490 | 0% |
|  | Duration | 0.0000 (-0.0001, 0.0001) | 0.1 | 0.775 | 22.8% |
|  | Year | 0.0000 (-0.0001, 0.0000) | 0.2 | 0.669 | 24.9% |
| Christmas Day | Design | -0.0002 (-0.0012, 0.0008) | 0.2 | 0.682 | 0.6% |
| (k=17) | Continent | NA | 1.3 | 0.517 | 1.4% |
|  | Country | NA | 5.3 | 0.914 | 67.5% |
|  | Duration | 0.0000 (0.0000, 0.0000) | 0.1 | 0.708 | 55.6% |
|  | Year | 0.0000 (0.0000, 0.0000) | 0 | 0.968 | 5.4% |
| New Year’s Day | Design | -0.0016 (-0.0037, 0.0005) | 2.0 | 0.158 | 56.8% |
| (K=16) | Continent | NA | 0.3 | 0.856 | 61.9% |
|  | Country | NA | 11.8 | 0.224 | 56.2% |
|  | **Duration** | -0.0001 (-0.0001, 0.0000) | 5.7 | **0.017*** | **38.1%** |
|  | Year | 0.0000 (0.0001, 0.0001) | 2.7 | 0.104 | 51.3% |

Abbreviation: CI=confidence interval; NA=not applicable; RR=risk ratio; QM=Q value of test of moderators

^a^ Year represents the starting year of each study.

^b^ I^2: Estimates how much of the residual heterogeneity is attributable to unaccounted variability in the meta-regression model. It is expressed as a percentage.

eTable 7. Meta-analysis of Non-affirmative Studies

| Risk ratio | Original | Meta-analysis of non-affirmative studies | Direction |
| --- | --- | --- | --- |
| Christmas Eve | 0.82 (0.64, 1.06) | 0.89 (0.55, 1.46); p=0.583 | consistent |
| Christmas Day | 0.83 (0.73, 0.95) | 0.87 (0.72, 1.05); p=0.130 | consistent |
| New Year | 1.34 (1.08, 1.66) | 1.14 (0.88, 1.48); p=0.255 | consistent |
| Valentine’s Day | 0.98 (0.82, 1.16) | 0.98 (0.89, 1.08); p=0.433 | consistent |
| Risk difference | Original | Meta-analysis of non-affirmative studies | Direction |
| Christmas Eve | -0.06% (-0.10%, -0.01%) | -0.04% (-0.13%, 0.04%); p=0.285 | consistent |
| Christmas Day | -0.04% (-0.11%, 0.03%) | -0.04% (-0.08%, -0.009%); p=0.024* | consistent |
| New Year | 0.10% (0.02%, 0.17%) | 0.04% (-0.04%, 0.12%); p=0.295 | consistent |
| Valentine’s Day | -0.01% (-0.05%, 0.04%) | -0.01% (-0.03%, 0.02%); p=0.410 | consistent |

eTable 8. Meta-analysis using Generalised Linear Mixed Model with Logit Transformation

| Outcome | Original | Generalised linear mixed models | Difference |
| --- | --- | --- | --- |
| Christmas Eve | 0.23% (0.17%, 0.28%) | 0.23% (0.18%, 0.29%) | 0 |
| Christmas Day | 0.23% (0.19%, 0.28%) | 0.25% (0.20%, 0.31%) | -0.02% |
| New Year | 0.39% (0.31%, 0.47%) | 0.39% (0.32%, 0.48%) | 0 |
| Valentine’s Day | 0.27% (0.24%, 0.30%) | 0.27% (0.24%, 0.31%) | 0 |

eTable 9. Leave-one-out sensitivity analysis for the Christmas eve

eTable 10. Leave-one-out sensitivity analysis for the Christmas day

eTable 11. Leave-one-out sensitivity analysis for the New year

eTable 12. Leave-one-out sensitivity analysis for the Valentine day

**Appendix 1. Checklist of PRISMA guideline**

| **Section and Topic** | **Item #** | **Checklist item** | **Location where item is reported** |
| --- | --- | --- | --- |
| **TITLE** | | |  |
| Title | 1 | Identify the report as a systematic review. | title |
| **ABSTRACT** | | |  |
| Abstract | 2 | See the PRISMA 2020 for Abstracts checklist. | Abstract section |
| **INTRODUCTION** | | |  |
| Rationale | 3 | Describe the rationale for the review in the context of existing knowledge. | 1st -2nd paragraph of introduction |
| Objectives | 4 | Provide an explicit statement of the objective(s) or question(s) the review addresses. | 3rd paragraph of introduction |
| **METHODS** | | |  |
| Eligibility criteria | 5 | Specify the inclusion and exclusion criteria for the review and how studies were grouped for the syntheses. | paragraph of " Study selection and outcomes " of methods |
| Information sources | 6 | Specify all databases, registers, websites, organisations, reference lists and other sources searched or consulted to identify studies. Specify the date when each source was last searched or consulted. | paragraph of " Literature search " of methods |
| Search strategy | 7 | Present the full search strategies for all databases, registers and websites, including any filters and limits used. | Appendix 2 |
| Selection process | 8 | Specify the methods used to decide whether a study met the inclusion criteria of the review, including how many reviewers screened each record and each report retrieved, whether they worked independently, and if applicable, details of automation tools used in the process. | paragraph of " Study selection and outcomes " of methods |
| Data collection process | 9 | Specify the methods used to collect data from reports, including how many reviewers collected data from each report, whether they worked independently, any processes for obtaining or confirming data from study investigators, and if applicable, details of automation tools used in the process. | paragraph of " Data Extraction and quality assessment" of methods |
| Data items | 10a | List and define all outcomes for which data were sought. Specify whether all results that were compatible with each outcome domain in each study were sought (e.g. for all measures, time points, analyses), and if not, the methods used to decide which results to collect. | paragraph of " Data Extraction and quality assessment " of methods |
|  | 10b | List and define all other variables for which data were sought (e.g. participant and intervention characteristics, funding sources). Describe any assumptions made about any missing or unclear information. | paragraph of "Data Extraction and quality assessment" of methods |
| Study risk of bias assessment | 11 | Specify the methods used to assess risk of bias in the included studies, including details of the tool(s) used, how many reviewers assessed each study and whether they worked independently, and if applicable, details of automation tools used in the process. | paragraph of "Data Extraction and quality assessment" of methods |
| Effect measures | 12 | Specify for each outcome the effect measure(s) (e.g. risk ratio, mean difference) used in the synthesis or presentation of results. | paragraph of " Statistical Analysis" of methods |
| Synthesis methods | 13a | Describe the processes used to decide which studies were eligible for each synthesis (e.g. tabulating the study intervention characteristics and comparing against the planned groups for each synthesis (item #5)). | paragraph of " Statistical Analysis" of methods |
|  | 13b | Describe any methods required to prepare the data for presentation or synthesis, such as handling of missing summary statistics, or data conversions. | paragraph of " Statistical Analysis" of methods |
|  | 13c | Describe any methods used to tabulate or visually display results of individual studies and syntheses. | paragraph of " Statistical Analysis" of methods |
|  | 13d | Describe any methods used to synthesize results and provide a rationale for the choice(s). If meta-analysis was performed, describe the model(s), method(s) to identify the presence and extent of statistical heterogeneity, and software package(s) used. | paragraph of " Statistical Analysis" of methods |
|  | 13e | Describe any methods used to explore possible causes of heterogeneity among study results (e.g. subgroup analysis, meta-regression). | paragraph of "Meta-regression and subgroup analysis" and "Publication bias and sensitivity analyses" of methods |
|  | 13f | Describe any sensitivity analyses conducted to assess robustness of the synthesized results. | paragraph of "Meta-regression and subgroup analysis" and "Publication bias and sensitivity analyses" of methods |
| Reporting bias assessment | 14 | Describe any methods used to assess risk of bias due to missing results in a synthesis (arising from reporting biases). | Not applicable |
| Certainty assessment | 15 | Describe any methods used to assess certainty (or confidence) in the body of evidence for an outcome. | Not applicable |
| **RESULTS** | | |  |
| Study selection | 16a | Describe the results of the search and selection process, from the number of records identified in the search to the number of studies included in the review, ideally using a flow diagram. | eFigure 1 (PRISMA 2020 flow diagram) |
|  | 16b | Cite studies that might appear to meet the inclusion criteria, but which were excluded, and explain why they were excluded. | Appendix 3 |
| Study characteristics | 17 | Cite each included study and present its characteristics. | eTable 1, 1^st^ paragraph of results |
| Risk of bias in studies | 18 | Present assessments of risk of bias for each included study. | paragraph of " Methodological Quality of Included Studies" of result  eFigure 2 |
| Results of individual studies | 19 | For all outcomes, present, for each study: (a) summary statistics for each group (where appropriate) and (b) an effect estimate and its precision (e.g. confidence/credible interval), ideally using structured tables or plots. | paragraph of “Suicide Risk and Proportion on Christmas Eve”, Christmas Day, and New Year’s Day”, “Proportion of Suicide, Risk Ratio, and Risk Difference on Valentine’s Day”, “Proportion of suicide on Christmas Eve, Christmas Day, and New Year’s Day by Country”, and “Proportion of Suicide, Risk Ratio, and Risk Difference of SHSB on the four holidays” of results  Figure 1-2 |
| Results of syntheses | 20a | For each synthesis, briefly summarise the characteristics and risk of bias among contributing studies. | eTable 2  paragraph of “Study selection” and “Methodological Quality of Included Studies” of result. |
|  | 20b | Present results of all statistical syntheses conducted. If meta-analysis was done, present for each the summary estimate and its precision (e.g. confidence/credible interval) and measures of statistical heterogeneity. If comparing groups, describe the direction of the effect. | paragraph of “Suicide Risk and Proportion on Christmas Eve”, Christmas Day, and New Year’s Day”, “Proportion of Suicide, Risk Ratio, and Risk Difference on Valentine’s Day”, “Proportion of suicide on Christmas Eve, Christmas Day, and New Year’s Day by Country”, and “Proportion of Suicide, Risk Ratio, and Risk Difference of SHSB on the four holidays” of results  Figure 1-2 |
|  | 20c | Present results of all investigations of possible causes of heterogeneity among study results. | paragraph of “Meta-analysis of non-affirmative studies and sensitivity analysis” of Results |
|  | 20d | Present results of all sensitivity analyses conducted to assess the robustness of the synthesized results. | paragraph of “Meta-analysis of non-affirmative studies and sensitivity analysis” |
| Reporting biases | 21 | Present assessments of risk of bias due to missing results (arising from reporting biases) for each synthesis assessed. | Not applicable |
| Certainty of evidence | 22 | Present assessments of certainty (or confidence) in the body of evidence for each outcome assessed. | Not applicable |
| **DISCUSSION** | | |  |
| Discussion | 23a | Provide a general interpretation of the results in the context of other evidence. | 1^st^ paragraph of discussion |
|  | 23b | Discuss any limitations of the evidence included in the review. | Paragraph of “Strengths and limitations of this study” of discussion |
|  | 23c | Discuss any limitations of the review processes used. | Paragraph of “Strengths and limitations of this study” of discussion |
|  | 23d | Discuss implications of the results for practice, policy, and future research. | Implications and conclusions section |
| **OTHER INFORMATION** | | |  |
| Registration and protocol | 24a | Provide registration information for the review, including register name and registration number, or state that the review was not registered. | Open Science Framework (osf.io/7zx3d) |
|  | 24b | Indicate where the review protocol can be accessed, or state that a protocol was not prepared. | Open Science Framework (osf.io/7zx3d) |
|  | 24c | Describe and explain any amendments to information provided at registration or in the protocol. | None |
| Support | 25 | Describe sources of financial or non-financial support for the review, and the role of the funders or sponsors in the review. | None |
| Competing interests | 26 | Declare any competing interests of review authors. | None |
| Availability of data, code and other materials | 27 | Report which of the following are publicly available and where they can be found: template data collection forms; data extracted from included studies; data used for all analyses; analytic code; any other materials used in the review. | Not applicable |

**Appendix 2. Search Strategy**

| Database |
| --- |
| PubMed search strategy |
| #1 "holidaying"[All Fields] OR "holidays"[MeSH Terms] OR "holidays"[All Fields] OR  "holiday"[All Fields] OR "vacation"[All Fields] OR "vacationed"[All Fields] OR  "vacationing"[All Fields] OR "vacations"[All Fields] OR "Christmas"[All Fields] OR  "New year"[All Fields] OR ("Valentine's"[All Fields] AND "Day"[All Fields]) OR  (("festive"[All Fields] OR "festivities"[All Fields] OR "festivity"[All Fields] OR  "holidays"[MeSH Terms] OR "holidays"[All Fields] OR "festival"[All Fields] OR  "festivals"[All Fields]) AND ("seasons"[All Fields] OR "seasonability"[All Fields] OR  "seasonable"[All Fields] OR "seasonably"[All Fields] OR "seasonal"[All Fields] OR  "seasonalities"[All Fields] OR "seasonality"[All Fields] OR "seasonally"[All Fields] OR  "seasonals"[All Fields] OR "seasons"[MeSH Terms] OR "seasons"[All Fields] OR  "season"[All Fields])) OR "special days"[All Fields] (16,100)  #2 "suicid"[All Fields] OR "suicidal ideation"[MeSH Terms] OR ("suicidal"[All Fields] AND  "ideation"[All Fields]) OR "suicidal ideation"[All Fields] OR "suicidality"[All Fields] OR  "suicidal"[All Fields] OR "suicidally"[All Fields] OR "suicidals"[All Fields] OR  "suicide"[MeSH Terms] OR "suicide"[All Fields] OR "suicides"[All Fields] OR "suicides" [All Fields] OR "suicided"[All Fields] OR "suiciders"[All Fields] OR (("suicid"[All Fields] OR "suicidal ideation"[MeSH Terms] OR ("suicidal"[All Fields] AND "ideation"[All Fields]) OR "suicidal ideation"[All Fields] OR "suicidality"[All Fields] OR "suicidal"[All Fields] OR "suicidally"[All Fields] OR "suicidals"[All Fields] OR "suicide"[MeSH Terms] OR "suicide"[All Fields] OR "suicides"[All Fields] OR "suicide s"[All Fields] OR "suicided"[All Fields] OR "suiciders"[All Fields]) AND ("risk"[MeSH Terms] OR "risk"[All Fields])) OR (("suicid"[All Fields] OR "suicidal ideation"[MeSH Terms] OR ("suicidal"[All Fields] AND "ideation"[All Fields]) OR "suicidal ideation"[All Fields] OR "suicidality"[All Fields] OR "suicidal"[All Fields] OR "suicidally"[All Fields] OR "suicidals"[All Fields] OR "suicide"[MeSH Terms] OR "suicide"[All Fields] OR "suicides"[All Fields] OR "suicide s"[All Fields] OR "suicided"[All Fields] OR "suiciders"[All Fields]) AND ("timely"[All Fields] OR "timing"[All Fields] OR "timings"[All Fields])) OR ("suicide, attempted" [MeSH Terms] OR ("suicide"[All Fields] AND "attempted"[All Fields]) OR "attempted suicide"[All Fields] OR ("suicide"[All Fields] AND "attempt"[All Fields]) OR "suicide attempt"[All Fields]) (122,541)  #3 #1 AND #2 (163) |
| Cochrane Central Register of Controlled Trials (CENTRAL) search strategy |
| #1 MeSH descriptor: [Holidays] explode all trees (44)  #2 holiday (414)  #3 (Christmas):ti,ab,kw (Word variations have been searched) (72)  #4 (Valentine's Day):ti,ab,kw (Word variations have been searched) (16)  #5 (festive season):ti,ab,kw (Word variations have been searched) (5)  #6 (special days):ti,ab,kw (Word variations have been searched) (7,969)  #7 #1 OR #2 OR #3 OR #4 OR #5 OR #6 (8,824)  #8 MeSH descriptor: [Suicide] explode all trees (2,151)  #9 suicide (6,353)  #10 (suicide risk):ti,ab,kw (Word variations have been searched) (3,463)  #11 suicide timing (296)  #12 MeSH descriptor: [Suicide, Attempted] explode all trees (664)  #13 suicide attempt (1,700)  #14 #8 OR #9 OR #10 OR #11 OR #12 OR #13 (9,440)  #15 #7 AND #14 (110) |
| Embase search strategy |
| #1. 'holiday'/exp OR holiday (53,232)  #2. 'christmas'/exp OR christmas (4,092)  #3. 'new year' (2,303)  #4. 'valentine day' (2)  #5. 'festive season' OR (festive AND ('season'/exp OR season)) (93)  #6. 'special days' (43)  #7. #1 OR #2 OR #3 OR #4 OR #5 OR #6 (58,847)  #8. 'suicide'/exp OR suicide (144,155)  #9. 'suicide risk'/exp OR 'suicide risk' (10,893)  #10. 'suicide timing' OR (('suicide'/exp OR suicide) AND ('timing'/exp OR timing)) (616)  #11. 'suicide attempt'/exp OR 'suicide attempt' (43,344)  #12. #8 OR #9 OR #10 OR #11 (144,155)  #13. #7 AND #12 (345) |
| PsycInfo search strategy |
| S1 holiday OR Christmas OR "New year" OR Valentine's Day OR festive season OR  "special days" (3,718)  S2 suicide OR suicide risk OR suicide timing OR suicide attempt (74,443)  S3 S1 AND S2 (94) |

CENTRAL: Cochrane Central Register of Controlled Trials

**Appendix 3. Reasons for Exclusion**

**No outcome of interest (n=74)**

1. Adams D, Taiwo F, Miller J, Adams V, Neil K. Dying one way or another: An analysis of combat and non-combat deaths among U.S. troops in Vietnam, 1960-1975. American Journal of Tropical Medicine and Hygiene. 2017;97(5):157.

2. Ajdacic-Gross V, Tran US, Bopp M, Sonneck G, Niederkrotenthaler T, Kapusta ND, et al. Understanding weekly cycles in suicide: An analysis of Austrian and Swiss data over 40 years. Epidemiology and Psychiatric Sciences. 2015;24(4):315-21.

3. Arendt F, Scherr S. Optimizing Online Suicide Prevention: A Search Engine-Based Tailored Approach. Health communication. 2017;32(11):1403-8.

4. Bierton C, Cashman K, Langlois NE. Is sudden death random or is it in the weather? Forensic Sci Med Pathol. 2013;9(1):31-5.

5. Blenkiron P. The timing of deliberate self harm behaviour. Irish Journal of Psychological Medicine. 2003;20(4):126-31.

6. Bollen KA. Temporal variations in mortality: a comparison of U.S. suicides and motor vehicle fatalities, 1972-1976. Demography. 1983;20(1):45-59.

7. Boyes AP. Repetition of overdose: a retrospective 5-year study. Journal of advanced nursing. 1994;20(3):462-8.

8. Brådvik L, Berglund M. A suicide peak after weekends and holidays in patients with alcohol dependence. Suicide and Life-Threatening Behavior. 2003;33(2):186-91.

9. Brown E. Physician-assisted suicide: reflections of a Maine family physician during the Jewish high holidays. J Am Acad Psychiatry Law. 2001;29(2):225-31.

10. Carnes JW, Brownlee Jr HJ, Aucremann CE. Are the holidays hazardous to your patients' health? Postgraduate Medicine. 1986;80(8):21-3.

11. Caudill CC. Physician assisted suicide, or be careful in which state you vacation in the next 12 months. Nebr Med J. 1996;81(6):157-8.

12. Chitty KM, Raubenheimer J, Cairns R, Kirby KA, Buckley NA. Deliberate self-poisoning in Australian adolescents is increased on school days. J Psychiatr Res. 2022;148:103-9.

13. Christensen R, Dowrick PW. Myths of mid-winter depression. Community Mental Health Journal. 1983;19(3):177-86.

14. Christoffel KK, Marcus D, Sagerman S, Bennett S. Adolescent suicide and suicide attempts: a population study. Pediatr Emerg Care. 1988;4(1):32-40.

15. Corcoran P, Reilly M, Salim A, Brennan A, Keeley HS, Perry IJ. Temporal Variation in Irish Suicide Rates. Suicide and Life-Threatening Behavior. 2004;34(4):429-38.

16. Daradkeh TK. Parasuicide during Ramadan in Jordan. Acta Psychiatrica Scandinavica. 1992;86(3):253-4.

17. Ettlinger R, Flordh P. Attempted suicide Experience of five hundred cases at a general hospital. Acta Psychiatrica et Neurologica (KjøBenhavn). 1956;Suppl. 106:300-1.

18. Fernández-Niño JA, Flórez-García VA, Astudillo-García CI, Rodríguez-Villamizar LA. Weather and suicide: A decade analysis in the five largest capital cities of Colombia. International Journal of Environmental Research and Public Health. 2018;15(7).

19. Flisher AJ, Joubert G, Yach D. Mortality from external causes in South African adolescents, 1984-1986. South African Medical Journal. 1992;81(2):77-80.

20. Franckenberg S, Sieberth T, Fliss B, Ebert L, Thali MJ, Dobay A. Thank God it’s Friday?—Correlation of the beginning and end of the week in general and Christmas holidays in particular with manner of death: A retrospective cohort study. Rechtsmedizin. 2022;32(3):179-83.

21. Frank ML, Lester D. Geophysical variables and behavior: LI Temporal variation of suicide in teens and young adults. Perceptual and Motor Skills. 1988;67(2):586-.

22. González-Manrique MA, Rodríguez-Llauger A. Epidemiological trends of suicide in Puerto Rico: 1931 to 1985. Puerto Rico health sciences journal. 1988;7(3):245-50.

23. Greiner T, Pokorny AD. Can death be postponed? The death-dip phenomenon in psychiatric patients. Omega: Journal of Death and Dying. 1989;20(2):117-26.

24. Grjibovski A, Kozhakhmetova G, Nurgaliyeva N, Adilbekova B, Kosbayeva A, Menne B. Daily suicide counts and ambient air temperature in Astana, Kazakhstan in 2006-2010. European Journal of Epidemiology. 2013;28(1):S123.

25. Grjibovski AM, Kozhakhmetova G, Kosbayeva A, Menne B. Associations between air temperature and daily suicide counts in Astana, Kazakhstan. Medicina (Kaunas). 2013;49(8):379-85.

26. Gunnell D, Caul S, Appleby L, John A, Hawton K. The incidence of suicide in University students in England and Wales 2000/2001–2016/2017: Record linkage study. Journal of Affective Disorders. 2020;261:113-20.

27. Haberhauer G, Fries W. [Epidemiology of attempted suicide]. Z Gesamte Inn Med. 1991;46(17):654-6.

28. Hall M, Fullerton L, Green D, Fitzgerald CA. Positive relationships with adults and resilience to suicide attempt among new mexico hispanic adolescents. International Journal of Environmental Research and Public Health. 2021;18(19).

29. Herea SG, Scripcaru C. Statistical analysis of suicide characteristics in Iaşi County. Revista medico-chirurgicalǎ̌ a Societǎ̌ţii de Medici ş̧i Naturaliş̧ti din Iaş̧i. 2012;116(3):674-80.

30. Hillard JR, Holland JM, Ramm D. Christmas and psychopathology. Data from a psychiatric emergency room population. Arch Gen Psychiatry. 1981;38(12):1377-81.

31. Ho BKW, Kua EH, Hong C. Temporal variation in parasuicide among Singaporean Chinese. Australian and New Zealand Journal of Psychiatry. 1998;32(4):500-3.

32. Hökby S, Westerlund J, Blazevska B, Hadlaczky G. Suicides during the Swedish midsummer holiday: analysis of cause of death data 1980-2018. Nord J Psychiatry. 2021;75(7):487-93.

33. Jenner B. Trends in ethanol-poisonings in the period December 2001 - August 2004, in the material of toxicological laboratory. Przegla̧d lekarski. 2005;62(6):602-7.

34. Jiménez-Hernández M, Castro-Zamudio S, Guzmán Parra J, Martínez-García AI, Guillén-Benítez C, Moreno-Küstner B. [Calls due to suicidal behaviour made to the prehospital, emergency department in Málaga: characteristics and associated factors]. An Sist Sanit Navar. 2017;40(3):379-89.

35. Joukamaa M. Prison suicide in Finland, 1969-1992. Forensic Sci Int. 1997;89(3):167-74.

36. Kayipmaz S, San I, Usul E, Korkut S. The effect of meteorological variables on suicide. International journal of biometeorology. 2020;64(9):1593-8.

37. Kim Y, Kim H, Kim D-S. Association between daily environmental temperature and suicide mortality in Korea (2001–2005). Psychiatry Research. 2011;186(2-3):390-6.

38. Lei C, Qu D, Liu K, Chen R. Ecological Momentary Assessment and Machine Learning for Predicting Suicidal Ideation Among Sexual and Gender Minority Individuals. JAMA network open. 2023;6(9):e2333164.

39. Lester D. Temporal variation in suicide and homicide. Am J Epidemiol. 1979;109(5):517-20.

40. Lester D. Suicide and homicide at Easter. Psychological Reports. 1987;61(1):224-.

41. Lester D. Suicide and homicide rates on national holidays. Psychological Reports. 1987;60(2):414-.

42. Lester D, Beck AT. Suicide and national holidays. Psychological Reports. 1975;36(1):52-.

43. Likhvar V, Honda Y, Ono M. Relation between temperature and suicide mortality in Japan in the presence of other confounding factors using time-series analysis with a semiparametric approach. Environmental Health and Preventive Medicine. 2011;16(1):36-43.

44. Lloyd B, Matthews S, Livingston M, Jayasekara H, Smith K. Alcohol intoxication in the context of major public holidays, sporting and social events: A time–series analysis in Melbourne, Australia, 2000–2009. Addiction. 2013;108(4):701-9.

45. Luo C. Associations between mortality, morbidity from injuries and meteorological factors in Hong Kong: A time-series study: ProQuest Information & Learning; 2020.

46. Mansbach JM, Wharff E, Austin SB, Ginnis K, Woods ER. Which psychiatric patients board on the medical service? Pediatrics. 2003;111(6 Pt 1):e693-8.

47. Marriott C, Harshbarger D. The hollow holiday: Christmas, a time of death in Appalachia. Omega: Journal of Death and Dying. 1973;4(4):259-66.

48. Masterton G. Monthly and seasonal variation in parasuicide: A sex difference. The British Journal of Psychiatry. 1991;158:155-7.

49. Mathew VM, Lindesay J, Shanmuganathan N, Eapen V. Attempted suicide and the lunar cycle. Psychol Rep. 1991;68(3 Pt 1):927-30.

50. Merrill RM. Injury-Related Deaths according to Environmental, Demographic, and Lifestyle Factors. Journal of Environmental and Public Health. 2019;2019.

51. Moreno-Küstner B, del Campo-Ávila J, Ruíz-Ibáñez A, Martínez-García AI, Castro-Zamudio S, Ramos-Jiménez G, et al. Epidemiology of suicidal behavior in Malaga (Spain): An approach from the prehospital emergency service. Frontiers in Psychiatry. 2019;10.

52. Nakamura JK, McLeod CR, McDermott JF. Temporal variation in adolescent suicide attempts. Suicide and Life-Threatening Behavior. 1994;24(4):343-9.

53. Neuner T, Hübner-Liebermann B, Wolfersdorf M, Felber W, Hajak G, Spiessl H. Time patterns of inpatient suicides. International Journal of Psychiatry in Clinical Practice. 2010;14(2):95-101.

54. Nguyen AM, Malig BJ, Basu R. The association between ozone and fine particles and mental health-related emergency department visits in California, 2005-2013. PLoS One. 2021;16(4):e0249675.

55. Nieto-Betancurt L, Fandiño-Losada A, Ponce de Leon A, Pacichana-Quinayaz SG, Gutiérrez-Martínez MI. Seasonal and Temporal Patterns of Homicides and Suicides in Cali and Manizales, Colombia: A Times-Series Analysis 2008-2015. Archives of suicide research : official journal of the International Academy for Suicide Research. 2023;27(1):43-62.

56. Nishi M, Miyake H, Okamoto H, Goto Y, Sakai T. Relationship between suicide and holidays. Journal of epidemiology / Japan Epidemiological Association. 2000;10(5):317-20.

57. Ohtsu T, Kokaze A, Osaki Y, Kaneita Y, Shirasawa T, Ito T, et al. Blue monday phenomenon among men: Suicide deaths in Japan. Acta Medica Okayama. 2009;63(5):231-6.

58. Peterson BS, Zhang H, Santa Lucia R, King RA, Lewis M. Risk factors for presenting problems in child psychiatric emergencies. J Am Acad Child Adolesc Psychiatry. 1996;35(9):1162-73.

59. Phillips DP, Carstensen LL. Clustering of teenage suicides after television news stories about suicide. The New England Journal of Medicine. 1986;315(11):685-9.

60. Phillips DP, Sanzone AG. A comparison of injury date and death date in 42,698 suicides. Am J Public Health. 1988;78(5):541-3.

61. Phillips DP, Wills JS. A drop in suicides around major national holidays. Suicide and Life-Threatening Behavior. 1987;17(1):1-12.

62. Phillips PD, Christenfeld N, Ryan NM. An increase in the number of deaths in the United States in the first week of the month: An association with substance abuse and other causes of death. New England Journal of Medicine. 1999;341(2):93-8.

63. Sapozhnikov S, Golenkov A, Rihmer Z, Ungvari GS, Gazdag G. WEEKLY PATTERNS of SUICIDE and the INFLUENCE of ALCOHOL CONSUMPTION in AN URBAN SAMPLE. Ideggyogyaszati Szemle. 2022;75(3-4):99-104.

64. Schwartz JG, Stuckey JH, Prihoda TJ, Kazen CM, Carnahan JJ. Hospital-based toxicology: patterns of use and abuse. Tex Med. 1990;86(6):44-51.

65. Stickley A, Sheng Ng CF, Inoue Y, Yazawa A, Koyanagi A, Kodaka M, et al. Birthdays are associated with an increased risk of suicide in Japan: Evidence from 27,007 deaths in Tokyo in 2001-2010. J Affect Disord. 2016;200:259-65.

66. Villeneuve PJ, Huynh D, Lavigne É, Colman I, Anisman H, Peters C, et al. Daily changes in ambient air pollution concentrations and temperature and suicide mortality in Canada: Findings from a national time-stratified case-crossover study. Environ Res. 2023;223:115477.

67. Wenz FV. Seasonal suicide attempts and forms of loneliness. Psychological reports. 1977;40(3 Pt 2):807-10.

68. Williams A, While D, Windfuhr K, Bickley H, Hunt IM, Shaw J, et al. Birthday blues: Examining the association between birthday and suicide in a national sample. Crisis: The Journal of Crisis Intervention and Suicide Prevention. 2011;32(3):134-42.

69. Wong MC. Case series of all completed suicide cases under the elderly suicide prevention programme of a mental hospital in Hong Kong from 2002-2010. Asia-Pacific Psychiatry. 2012;4:97-8.

70. Wu Y-W, Chen C-K, Wang L-J. Is suicide mortality associated with meteorological and socio-economic factors? An ecological study in a city in Taiwan with a high suicide rate. Psychiatria Danubina. 2014;26(2):152-8.

71. Yarza S, Vodonos A, Hassan L, Shalev H, Novack V, Novack L. Suicide behavior and meteorological characteristics in hot and arid climate. Environmental Research. 2020;184.

72. Yip PS, Chi I, Yu KK. An epidemiological profile of elderly suicides in Hong Kong. Int J Geriatr Psychiatry. 1998;13(9):631-7.

73. Yoshida L, Griffin E, Arensman E, Corcoran P. Ireland's alcohol problem: A public health issue. Alcoholism: Clinical and Experimental Research. 2018;42:97A.

74. Zung WW, Green RL. Seasonal variation of suicide and depression. Archives of General Psychiatry. 1974;30(1):89-91.

**Conference Abstract and Review (n=11)**

1. De Vries I, Koppen A, Hunault CC. Seasonality in intentional drug intake by adolescents. Clinical Toxicology. 2017;55(5):476-7.

2. Su M, Lane K, Ito K, Hoffman RS. Are intentional suicidal overdoses temporally associated with season of the year? Clinical Toxicology. 2015;53(7):757.

3. Vimmer M, Cihal L, Bartakova M, Laufíková M, Domluvilova D, Poradovska J, et al. Environmental correlations of acute psychiatric admissions and suicides in Prague, Czech Republic. Results of pilot study. European Psychiatry. 2009;24:S817.

4. Beauchamp G, Ho M, Yin S. Variation in suicide occurrence by day and during major American holidays. Clinical Toxicology. 2012;50(7):642-3.

5. Buckley NA, Dawson AH, Whyte IM. There are days ... and moons ... and public holidays. Self-poisoning is not lunacy. Med J Aust. 1994;161(11-12):728.

6. Carley S, Hamilton M. Best evidence topic report. Suicide at christmas. Emerg Med J. 2004;21(6):716-7.

7. Martin SJ, Kelly IW, Saklofske DH. Suicide and lunar cycles: A critical review over 28 years. Psychological Reports. 1992;71(3, Pt 1):787-95.

8. Ploderl M. Suicide risk over the course of the day, week, and life. Psychiatria Danubina. 2021;33(3):438-45.

9. Rosen Y, Iancu I. The yearly distribution of suicide and parasuicide. Harefuah. 2002;141(11):979-82, 1009.

10. Sansone RA, Sansone LA. The Christmas effect on psychopathology. Innovations in Clinical Neuroscience. 2011;8(12):10-3.

11. Schneider E, Liwinski T, Imfeld L, Lang UE, Brühl AB. Who is afraid of Christmas? The effect of Christmas and Easter holidays on psychiatric hospitalizations and emergencies-Systematic review and single center experience from 2012 to 2021. Front Psychiatry. 2022;13:1049935.

**Duplicated Data (n=2)**

1. Ajdacic-Gross V, Wang J, Bopp M, Eich D, Rössler W, Gutzwiller F. Are seasonalities in suicide dependent on suicide methods? A reappraisal. Social Science & Medicine. 2003;57(7):1173-81.

2. Bozsonyi K, Veres E, Zonda T. The effect of public holidays on the suicide drive (frequency) in Hungary (1970-2002). Psychiatria Hungarica : A Magyar Pszichiátriai Társaság tudományos folyóirata. 2005;20(6):463-71.

**Full text not available (n=6)**

1. Aveline F, Baudelot C, Beverraggi M, Lahlou S. [Suicide and social rhythms]. Econ Stat. 1984(168):71-6, 117, 20.

2. Blachly PH, Fairley N. Market analysis for suicide prevention. Relationship of age to suicide on holidays, day of the week and month. Northwest medicine. 1969;68(3):232-8.

3. Bostwick JE. The effects of holidays on rates of suicide attempts: ProQuest Information & Learning; 1992.

4. Károly B, Előd V, Tamás Z. Az ünnepek hatása az öngyilkossági hajlandóságra Magyarországon (1970-2002) = The effect of public holidays on the suicide drive (frequency) in Hungary (1970-2002). Psychiatria Hungarica. 2005;20(6):463-71.

5. Olsson L, Wasserman D. Increased risk of suicide among young people on New Year's day. Läkartidningen. 1999;96(50):5677-8.

6. Stefanini P, Biggeri A, Geddes M, Comodo N. [Analysis of temporal distribution of suicides. Methodological notes on comparison of dates of death and dates of suicidal act]. Epidemiol Prev. 1990;12(42):23-9.

**Appendix 4. Details of Data Extraction on the Number of Suicides During Holidays and Regular Days**

| **Study name** | **Holiday** | **Regular day** |
| --- | --- | --- |
| Ajdacic-Gross 2008 | Mean suicide on holidays | Mean daily suicide frequency |
| Akkaya-Kalayci 2015 | Total suicide attempts on holidays | Mean daily suicide attempt |
| Arango 2016 | Mean suicide on holidays | Mean daily suicide frequency |
| Baker 2014 | Mean suicide on holidays | Overall daily average suicides |
| Beauchamp 2014 | Mean suicide attempt on holidays | Mean suicide attempt on control days |
| Bergen 2007 | By calculating difference in suicide attempts compared with regular days | Total suicide attempts divided by follow-up days |
| Bridges 2004 | Mean suicide on holidays | Mean daily suicide frequency |
| Cavanagh 2016 | Reverse-engineering the mean suicide on holidays from the risk ratio | Total suicide attempts divided by follow-up days |
| Cullum 1993 | Total suicide attempts on holidays | Total suicide attempts on control days |
| Davenport 1990 | Total suicide attempts on holidays | Total suicide attempts on control days |
| Deisenhammer 2018 | Mean suicide on holidays | Reverse-engineering total suicide on regular day from suicide on non-holiday |
| Fernández-Niño 2016 | Mean suicide on holidays | Total suicide divided by follow-up days |
| Griffin 2017 | Mean suicide attempt on holidays | Mean daily suicide attempt frequency |
| Hadlaczky 2018 | Mean suicide on holidays | Mean daily suicide frequency |
| Hofstra 2018 | Mean suicide on holidays | Total suicide divided by follow-up days |
| Jessen 1999a | Observed number of suicide on holidays | Total suicide divided by follow-up days |
| Jessen 1999b | Calculating changes in the percentage of suicides (reference: mean daily suicide) | Total suicide divided by follow-up days |
| Jones 1977 | Total suicide on holidays | Reverse-engineering total suicide on regular day from suicide on non-holiday |
| Lester 1985 | Total suicide attempts on holidays | Total suicide attempts on control days |
| Lester 1990 | Mean suicides on holidays | Reverse-engineer the mean suicides on control days from the t-value of the t-test comparing suicides during holidays and control days. |
| Liang 2011 | Changes in percentage of suicide attempts on holidays vs those on regular days | Total suicide attempts divided by follow-up days. |
| Philip 1980 | Calculating changes in the mean observed suicides on holidays | Mean daily suicide frequency on control days |
| Plöderl 2014 | Mean suicide on holidays | Mean daily suicide frequency |
| Rochoy 2024 | Total suicide attempts on holidays | Total suicide attempts divided by follow-up days |
| Sohn 2017 | Mean suicide on holidays | Total suicide divided by follow-up days |
| Sparhawk 1987 | Observed total suicides on holidays | Total suicide divided by follow-up days |
| Su 2020 | Mean suicide on holidays | Mean suicide on control days |
| Zonda 2009 | Calculating changes in the mean observed suicides on holidays | Total suicide divided by follow-up days |

^a^ All calculated values have been rounded to the nearest integer.

^b^ If the number of suicides over a one-year period was not available, it was calculated by multiplying the mean daily suicide frequency by 365 and rounding up to the nearest integer. Similarly, if the mean number of daily suicide frequency was not available, it was calculated by divided the total number of suicide over a one-year period by 365 an rounding up to the nearest integer.
